# Synchrotron cryo-soft X-ray microscopy reveals bacterial interactions with nanostructured calcium phosphate substrates

**DOI:** 10.1038/s41467-026-75323-y

**Published:** 2026-07-13

**Authors:** Marc Iglesias-Fernandez, Ana Joaquina Pérez-Berná, Eva Pereiro, Maria-Pau Ginebra, Montserrat Espanol

**Affiliations:** 1 Biomaterials, Biomechanics and Tissue Engineering Group, Department of Materials Science and Engineering, Av. Eduard Maristany, Universitat Politècnica de Catalunya - BarcelonaTech (UPC), Barcelona, Spain; 2 Barcelona Research Centre in Multiscale Science and Engineering, Av. Eduard Maristany, Universitat Politècnica de Catalunya - BarcelonaTech (UPC), Barcelona, Spain; 3 Experiments Division, MISTRAL Beamline, ALBA Synchrotron Light Source, Barcelona, Spain; 4 CIBER de Bioingeniería, Biomateriales y Nanomedicina (CIBER BBN), Instituto de Salud Carlos III, Madrid, Spain

**Keywords:** Bacterial techniques and applications, Biomaterials - cells, Imaging techniques, X-ray tomography, Biophysics

## Abstract

Nanostructured synthetic bone grafts offer a promising strategy to prevent bacterial colonisation while supporting bone regeneration. Here, calcium-deficient hydroxyapatite (CDHA) nanopillars synthesized from α-tricalcium phosphate exhibit contact-killing activity against Gram-positive *Bacillus subtilis*. Unlike inert bactericidal surfaces, CDHA exchanges ions with the surrounding environment, introducing chemical interactions alongside mechanical damage. Using synchrotron cryo-soft X-ray tomography and spectromicroscopy, we visualise bacterial ultrastructure and intracellular ionic changes in fully hydrated cells at subcellular resolution. Some bacteria exposed to the nanotopographies display membrane rupture, cytosolic leakage, and multivesicular body formation, indicating severe stress responses. XANES cryo-spectromicroscopy and linear absorption coefficient analysis identify a bacterial subpopulation with increased intracellular calcium, correlating with calcium release from the substrate and confirmed by confocal microscopy. However, this calcium accumulation does not affect viability, consistent with the halotolerant nature of *B. subtilis*. These findings provide insights into the combined effects of nanotopography and surface chemistry on bacterial responses.

## Abstract (web summary)

Nanotopographies help combat bacterial colonization. Here, authors use synchrotron cryo-soft X-ray microscopy to visualize the 3D ultrastructure of damaged bacteria and quantify single-cell calcium uptake on nanostructured calcium phosphate surfaces.

## Introduction

Calcium phosphates (CaPs) are the most widely used synthetic bone grafts to treat bone defects caused by trauma, disease, surgery, or congenital malformations. Among them, hydroxyapatite and calcium-deficient hydroxyapatite (CDHA) are particularly favored for bone tissue regeneration due to their biocompatibility, osseointegration, and osteoconductive properties^1^. These synthetic grafts can be engineered in various forms, such as granules, blocks, or porous scaffolds, tailored for applications in bone filling or bone augmentation. However, clinical procedures involving their application are not without risks. Postoperative infections can pose a challenge, compromising the success of bone grafts^2^. The current standard treatment for preventing or treating such infections is antibiotic administration. However, the increasing antibiotic resistance calls for alternatives to combat biomaterial-associated infections^3^.

A potential solution to the challenges associated with antibiotic resistance is the development of antimicrobial surfaces and, among them, the recently discovered antimicrobial nanostructured topographies^4^. These surfaces consist of nano-sized “protrusions,” such as nanopillars or nanoneedles^5^, engineered to disrupt the cell wall upon attachment. CaPs, when synthesized at low temperature, such as those involved in the processing of CaP cements and biomimetic precipitation, can efficiently lead to nanotopographies consisting of nano-size needles, rods, and plates^6^. Our research group has pioneered the development of synthetic CaPs featuring nanostructured bactericidal topographies capable of mechanically killing Gram-negative bacteria^7^.

The primary cause of bacterial death on antimicrobial topographies is believed to be the mechanical lysis of the cell wall caused by the high strain localized between the contact points with pillars^5,8^. However, the precise mechanisms governing this antimicrobial process remain elusive. It should be noted that in most of the published work on antimicrobial topographies, the generation of nanostructures has been carried out on chemically inert substrates, such as black silicon, gold, diamond, or titanium^9^. However, when these are developed on bioactive substrates, such as calcium phosphates^10^, the situation becomes more complex. The question arises to what extent chemistry, ionic exchanges with the environment, can influence the microbial response.

In this work, we elucidate the interaction of a Gram-positive bacterium, *B. subtilis*, with nanostructured calcium phosphate topographies, considering the effects of both the substrate topography and chemistry, by exploiting the versatility of synchrotron-based cryo-soft-X-rays (cryo-SXR) transmission microscopy. Although a limitation of this technique is that it does not allow imaging cells directly attached to solid substrates and, therefore, the spatial relationship between bacteria and the nanopillars cannot be directly visualized, this approach enables evaluating how exposure to different nanotopographies influences bacterial morphology, subcellular organization, and calcium internalization. Cryo soft X-ray tomography (cryo-SXT) enables three-dimensional visualization of whole hydrated, unstained cells at the nano-scale resolution^11^. Soft X-rays offer opportunities to study biological samples due to their specific interaction with matter in the so-called “water window” energy range (284–543 eV) between the carbon and the oxygen K absorption edges. In this range, X-rays have high penetration depth in water (up to ∼10 µm)^12^, while at 520 eV, the absorption by carbon-rich molecules—such as lipids and nucleic acids—is maximized. Cryo-SXT consists in obtaining 2D transmission projections at multiple angles with a specific lens on a CCD detector, which are then 3D reconstructed. Segmentation of the reconstructions allows the quantification of the linear absorption coefficients (LACs) at the corresponding energy.

In addition to tomography, cryo-SXR enables characterization by spectroscopy of different elements through their absorption edges within the water window via cryo-XANES microscopy (X-ray absorption near-edge structure). By acquiring images at sequential energies across the absorption edge of a given element, cryo-XANES provides pixel-by-pixel absorption spectra, allowing both the identification and its spatial location through 2D elemental mappings. Notably, the Ca L_2,3_ absorption edge (∼346 and 356 eV) lies within the water window. Therefore, imaging through this absorption edge makes it possible to characterize the presence of Ca in hydrated whole cells. This approach has proven very valuable for characterizing biomineralization processes, for example, in algae^13^, sea urchin larval cells^14^, or eukaryotic cells^15^, and may provide information to better understand the interaction of bacteria with bioactive calcium phosphate substrates.

## Results

### Topographies characterization

**Fig. 1:**
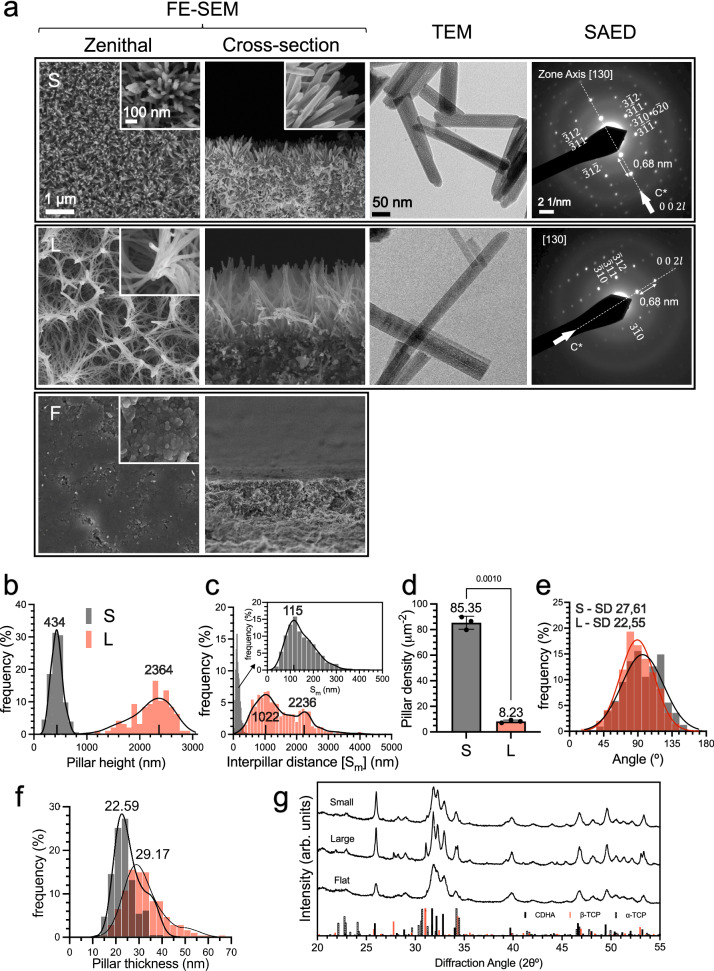
Physico-chemical characterization of the calcium phosphate substrates with different nanotopographies: small pillars (S), large pillars (L), and flat (F). **a** First two columns: FE-SEM images of the surface and the cross-section of the discs, respectively; third and fourth columns: TEM images and SAED pattern of a single pillar, respectively. **b** Pillar height; **c** interpillar distance; **d** pillar density; **e** pillar orientation, and **f** pillar diameter, all of them quantified from the FE-SEM images. Numbers written on top of the histograms refer to the peak value. Values given for (**d**) pillar density are mean ± standard deviation, and differences were determined by a two-sided Welch’s t-test (*P* < 0.05); **g** XRD of the different substrates. The bottom bars indicate the pattern of each phase according to JCPDS cards. FE-SEM micrographs and physico-chemical characterization are representative of three surfaces (*n* = 3), with each surface visualized in at least three different areas. Source data are provided in the Source data file.

Two nanostructured topographies, with small and large pillars (coded as S and L, respectively), were obtained by hydrolysis of α-tricalcium phosphate (α-TCP) discs to CDHA under different reaction conditions (Fig. 1a, b, and Table 1). Hydrolysis in water at 37 °C for 1 week produced a densely packed topography with small pillars. In contrast, hydrolysis in boiling water (100 °C) for 1 h generated large pillars clustered in pocket-like structures, resulting in a lower packing density and a bimodal distribution of interpillar distances (Fig. 1c, d). Both topographies exhibited predominantly vertical pillar orientation, with deviations up to ±45° (Fig. 1e). Pillar thickness, as determined by transmission electron microscopy (TEM) and field emission scanning electron microscopy (FE-SEM), was similar across both groups, ranging from 22.2 to 29.6 nm (Fig. 1f). A flat control surface was obtained compacting CDHA powder obtained by α-TCP powder hydrolysis at 37 °C for 1 week (Fig. 1a, bottom row).

**Table 1:** Topographical parameters, together with phase quantification and crystallite size obtained from XRD of the different crystalline phases present on the disks

		Small pillars (S)	Large pillars (L)	Flat (F)
Surface nanoarchitecture				
Pillar density [μm^−2^]		85.35 ± 5.08	8.23 ± 1.04	
Pillar interspacing [nm]		115	1022 and 2236	
Pillar height [nm]		434	2364	
Pillar thickness [nm]		29.6	22.2	
Phase composition (XRD)				
CDHA	Phase ± ESD (wt%)	94.1 ± 0.4	81.8 ± 0.5	95.4 ± 0.4
β-TCP	Phase ± ESD (wt%)	3.7 ± 0.4	14.8 ± 0.4	2.5 ± 0.3
α-TCP	Phase ± ESD (wt%)	2.2 ± 0.3	3.5 ± 0.3	2.1 ± 0.4
* χ*^2^		1.2906	1.6017	1.2665
Crystallite size (nm)		36.5 ± 1.7	48.6 ± 2.4	25.7 ± 1.7

For surface nanoarchitecture, pillar density is given as the mean value ± standard deviation; all the other parameters correspond to the peak or peaks of the distribution. Average phase composition and estimated standard deviation (ESD) is given in wt%, and crystallite size is estimated by the Scherrer formula (Eq. 1) using the (211) peak (*n* = 3). Source data is provided in the Source data file.

X-ray diffraction (XRD) confirmed CDHA as the main phase in all samples (Fig. 1g). Phase composition analysis by Rietveld (Table 1) confirmed that hydrolysis at 37 °C (small pillars and flat samples) resulted in CDHA with minor traces of unreacted α-TCP and β-TCP, the latter likely stabilized during the quenching process. The hydrolysis in boiling water (large pillars) resulted in a higher proportion of β-TCP, with similar levels of unreacted α-TCP. As expected, the crystallite size was larger for the L than for the S substrates, while F had the smallest crystalline domains. Complete data of the XRD and crystallite size analysis is displayed in Fig. S1 and Table S1 of the Supplementary Information.

Selected area electron diffraction (SAED) patterns for small and large pillars displayed monocrystalline diffraction patterns (Fig. 1a), with interplanar distances consistent with the CDHA monoclinic phase and a P2_1_/b space group (Fig. 1a). The brighter central line of spots in the SAED patterns is characteristic of the crystal C-axis. Along with Raman characterization results in a previous study^7^, these results confirm that CDHA is the only phase exposed on the surface, while α- and β-TCP phases are likely confined in the interior of the samples.

### Antibacterial activity

**Fig. 2:**
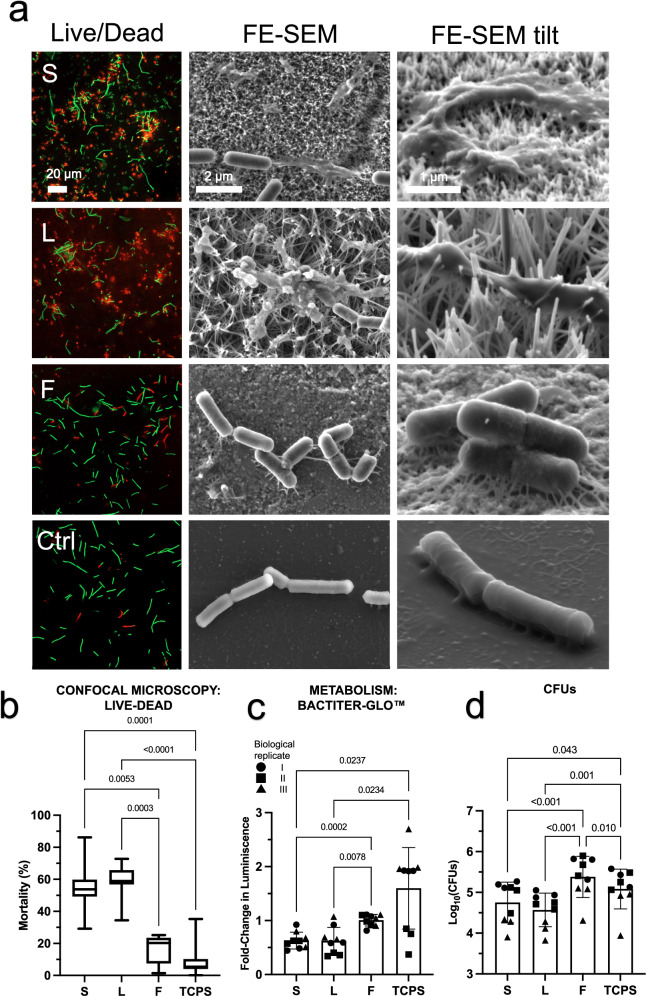
*B. subtilis* mortality on the nanostructured CDHA topographies. Bacteria were incubated on the different substrates for 3 h: nanostructured CDHA substrates with small and large nanopillars (S and L, respectively), flat CDHA (F), and glass coverslips (Ctrl). **a** First column: CSLM images of bacteria after live/dead BacLight staining. Red identifies dead bacteria, while green denotes viable cells. Second and third columns: top-view and tilted FE-SEM micrographs showing bacteria adhesion on different substrates. **b** Boxplot displaying bacteria mortality, expressed as the percentage of dead cells relative to the total population adhered, obtained from CSLM images. Box plots (*n* = 12) indicate median (middle line), 25th, 75th percentile (box), and 5th and 95th percentile (whiskers). **c** Bacterial viability assessment by metabolic activity (Bactiter-Glo) normalized to the F control (*n* = 9). **d** Bacterial viability assessment by CFUs counting of the total viable bacteria adhered to the topographies (*n* = 9). For **c**, **d**, data are presented as mean ± standard deviation, and the dot shape indicates the corresponding biological replicate. For live/dead mortality (**b**) and Bactiter-Glo™ (**c**), statistically significant differences were determined using one-way ANOVA. For CFU counting (**d**), statistically significant differences were determined using a mixed-effects model with biological replicates treated as random effects, followed by a Kenward–Roger test for multiple comparisons between topographies as a fixed effect (two-sided, *P* < 0.05). Source data is provided in the Source data file, and microscopy files are available on the Zenodo public repository^59^. FE_SEM micrographs are representative of three independent surfaces (*n* = 3).

The antimicrobial activity of the CDHA surfaces against the Gram-positive bacterium *B. subtilis* was evaluated after 3 h of incubation by a static adhesion assay, which is typically suited for evaluating contact-killing surfaces^16^. Figure 2 shows confocal scanning laser microscopy images of live/dead-stained samples, where cells are differentiated based on the absence or presence of cell wall damage. The bactericidal effect was significantly higher on both the small and large nanopillars than on the flat surface or glass coverslip controls (Ctrl), as evidenced by the increased presence of red-stained bacteria. No statistically significant differences in mortality were found between the two nanostructured topographies (L and S), nor between the two flat surfaces (F and Ctrl) (Fig. 2b). In agreement with these findings, Bactiter-Glo assays indicated a significant reduction in metabolic activity on the nanotopographies compared with the flat substrate (Fig. 2c). CFU analysis further supported this trend, confirming a reduction in bacterial viability on the nanostructured surfaces relative to the flat control (Fig. 2d).

The FE-SEM micrographs (Fig. 2a) revealed deflated or structurally compromised cells on the nanostructured topographies, in contrast to the intact bacteria adhered to the flat CDHA and glass coverslip. Tilted FE-SEM views further supported the presence of bacteria exhibiting loss of structural integrity on nanostructured topographies, consistent with the mechanobactericidal effect.

### Substrate reactivity and chemical interactions

**Fig. 3:**
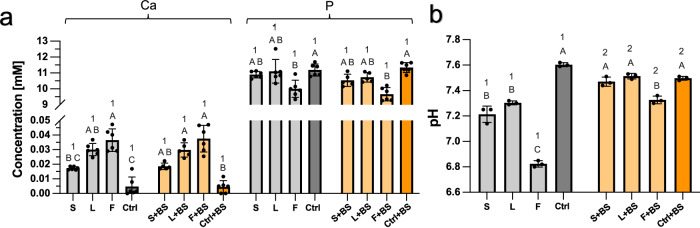
Calcium, phosphate, and pH levels in the medium surrounding the different CDHA nanotopographies, in the presence or absence of *B. subtilis*. **a** Ca and P concentrations in the supernatant after 3 h incubation in PBS in the absence (grey) or in the presence of *B. subtilis* (orange), assessed by ICP (*n* = 6, two biological replicates). **b** pH registered in the supernatant in the same conditions (*n* = 3). The controls were PBS (Ctrl) and PBS with bacteria (Ctrl + BS), without CDHA substrates. Bars represent mean ± s.d. Statistical differences were determined using a Kruskal–Wallis followed by Dunn’s post hoc test (two-sided, *P* < 0.05) for ion release, and one-way ANOVA followed by Tukey’s post hoc test (two-sided, *P* < 0.05) for pH. Groups sharing the same superscript are not statistically different. Capital letters indicate differences between topographies under the same incubation condition; Numbers indicate differences between PBS or bacteria inoculum for a given topography. Exact *P*-values are provided in the Source data file.

The Ca and P concentrations in the surrounding medium (phosphate buffer solution, PBS) after incubation for 3 h in the absence or in the presence of bacteria were measured by inductively coupled plasma (ICP) spectroscopy (Fig. 3a). All CDHA materials exhibited a mild release of Ca into PBS, more pronounced for the F and L substrates, regardless of bacteria presence. P levels remained stable across most conditions, except for the F substrate, which showed a statistically significant decrease both with and without bacteria.

The pH evolution after 3 h incubation is shown in Fig. 3b. All CDHA samples induced a slight acidification of the PBS, more pronounced for the F substrate. The presence of bacteria slightly acidified the PBS control group but, in contrast, mitigated the acidification produced by the CDHA substrates.

**Fig. 4:**
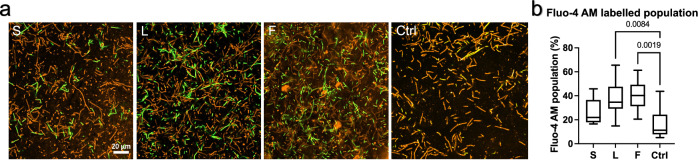
Quantification of intracellular calcium in the bacterial population adhered to the substrates. **a** Confocal microscopy images of *B. subtilis* incubated on CDHA substrates (S, L, and F) or the culture well (Ctrl). Intracellular calcium was detected using Fluo-4 AM (green), while the total population was labeled with CellBrite™ Fix 555 (orange). **b** Quantification of bacteria stained with Fluo-4 AM relative to the total CellBrite-labelled bacteria, expressed as area percentage (*n* = 12). Box plots indicate the median (middle line), 25th and 75th percentile (box), and 5th and 95th percentile (whiskers). No outliers were observed. Statistical differences were determined using a Kruskal–Wallis test followed by Dunn’s post hoc test (two-sided, *P* < 0.05). Source data is provided in the Source data file, and microscopy files are available on the Zenodo public repository^60^.

Fluo-4 AM is a cell-permeable Ca indicator that is metabolized by intracellular esterase, leading to a bright green fluorescent signal upon Ca^2+^ binding, providing sensitive information on intracellular calcium. Bacteria adhered on the different substrates were stained with Fluo-4 AM and visualized by confocal microscopy. The stained population was normalized by the total population adhered using the membrane marker CellBrite™ Fix 555 (Fig. 4). The control exhibited the lowest proportion of Fluo-4 AM-positive bacteria. Small pillars showed no statistically significant differences from the control. In contrast, both the large pillars and the flat substrate induced significantly higher proportions of Fluo-4 AM-positive bacteria compared to the control group.

### Investigation of the bacteria–substrate interaction by synchrotron-based cryo-soft X-ray microscopy

#### Cryo-soft X-ray tomography

Bacteria were imaged by cryo-soft X-ray tomography (cryo-SXT). Tomograms of vitrified cells were acquired at 520 eV where carbon-rich elements like proteins, lipids, and nucleic acids exhibit a strong absorption contrast^17^. Imaged bacteria were detached from the nanostructured CDHA surfaces, the flat CDHA surface, or the culture well after 3 h of incubation.

**Fig. 5:**
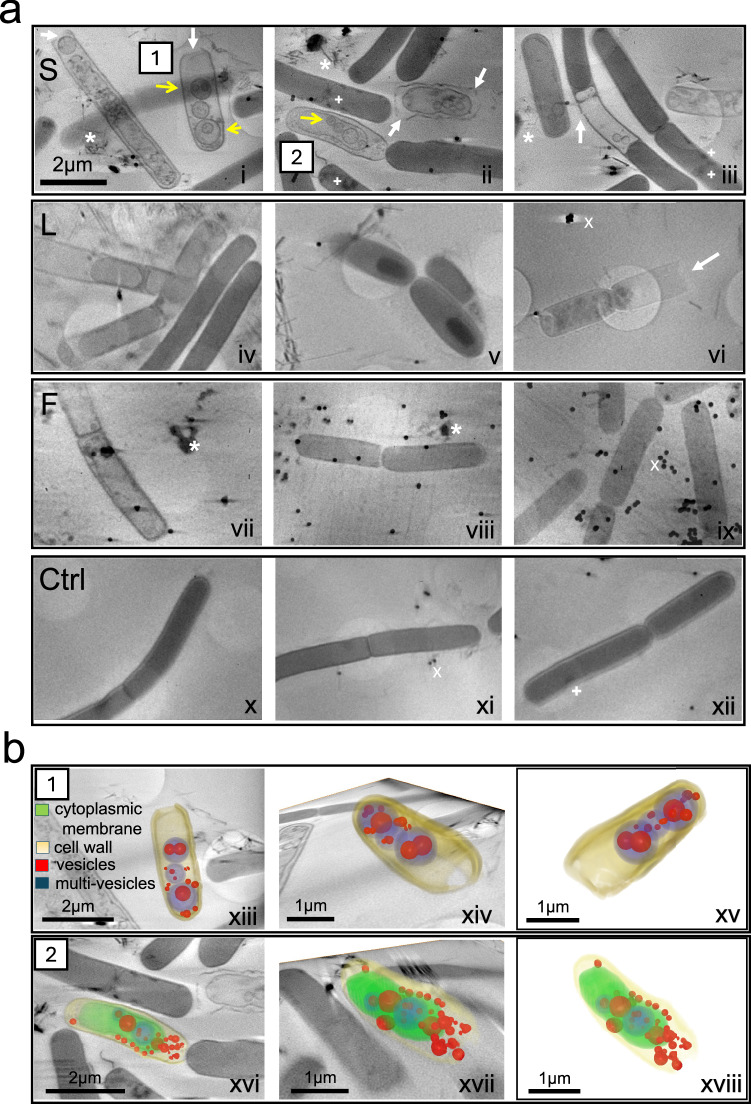
Bacteria ultrastructure and 3D segmentation of *B. subtilis* incubated on CDHA substrates or in the culture well. **a** Virtual slices of reconstructed tomographies acquired at 520 eV of vitrified bacteria after 3 h incubation on CDHA substrates with small pillars (i–iii), large pillars (iv–vi), flat CDHA (vii–ix), and without substrate (x–xii). Images show bacterial cells, CDHA crystals (*), gold fiducials (x), and some circular holes in the background from the support film. White arrows indicate disrupted cell walls. Bacteria incubated on small pillars (i–iii) exhibited multi-vesicular body formation (yellow arrows) and localized high-absorption regions (+). Endospore formation was observed in bacteria on large pillars (iv–vi), whereas fewer signs of membrane rupture were found in bacteria incubated on flat (vii–ix) and without control (x–xii). Images are representative of multiple independent tilt series acquired at 520 eV (small pillars, *n* = 14; large pillars, *n* = 9; flat CDHA, *n* = 15; control, *n* = 20. **b** 3D segmentation of bacteria incubated on small pillars (1-i, and 2-ii). Segmented volumes display the cytoplasmic membrane (green), multivesicular bodies (blue), cell wall (yellow), and vesicles (red). Segmentation was performed on two bacteria interacting with small pillars. The displayed tilt series and tomograms are publicly available in the EMPIAR repository^61^.

Cryo-soft X-ray tomography sections are shown in Fig. 5. In addition to bacteria, other features can be identified: the holes of the C support film used for vitrification, some highly absorbing gold fiducials of 100 nm used for tomogram alignment, and, in the samples incubated on CDHA substrates, detached CDHA crystals that were transferred during sample preparation. Notably, CDHA crystals can be distinguished from gold fiducial markers at 520 eV by their lower X-ray absorption contrast, far from the calcium absorption L-edges (∼350 eV). In addition, gold fiducials display a characteristic spherical morphology, in contrast to the elongated high-aspect ratio nanopillars observed for CDHA crystals in S and L topographies, or the clustered crystals irregular morphologies present on the flat surface (Fig. 5a). Tomographies revealed distinct features in the bacteria incubated on nanostructured topographies (Fig. 5), including damaged cell walls (white arrows), highly absorbing intracellular spots (+), and damaged bacteria showing a lighter absorbance, consistent with cytosol leakage. Bacterial ultrastructure in cells incubated on small pillars (Fig. 5a-i–iii) revealed the formation of vesicles and multi-vesicular bodies (yellow arrows), indicative of a stress response. In contrast, bacteria incubated on large pillars (Fig. 5a-iv–vi) did not display multivesicular structures but still exhibited signs of extensive damage, including disrupted cell walls (Fig. 5a-vi) and the formation of endospores (Fig. 5a-v), suggesting a stress response. The tomographic data allowed a 3D representation of the bacteria ultrastructure, including cell wall disruptions and segmentation of intracellular organelles, such as vesicles and multivesicular bodies, as shown in Fig. 5b and Supplementary Movies 1 and 2 (Supplementary Information), for bacteria 1 and 2 from Fig. 5a-i, ii, respectively. These 3D renderings revealed the spatial distribution and morphological diversity of vesicles within the bacteria, highlighting variations in their shape, size and sphericity.

#### Calcium distribution and XANES spectra

Cryo-soft X-rays can provide structural and chemical information through X-ray absorption near-edge structure (XANES) spectroscopy. Given the ionic exchange observed in the ICP analysis, we were specifically interested in calcium. To differentiate Ca from carbon-dense structures, such as membranes or vesicles, the incident photon energy was scanned across the Ca L-edge. This approach enabled the acquisition of individual XANES spectra from 2D image stacks with 0.1 eV energy steps across the spin-orbit L_3_ (349 eV) and L_2_ (352.4 eV) absorption peaks (Supplementary Information, Supplementary Movie 3). In this way, a full XANES spectrum can be obtained from each pixel in the image (pixel size = 13 nm). In addition, since the absorption variations from other elements are minimal within this energy range, subtracting a pre-edge image (346 eV) from an edge image (L_2_ = 352.4 eV) virtually isolates Ca, resulting in 2D Ca maps.

**Fig. 6:**
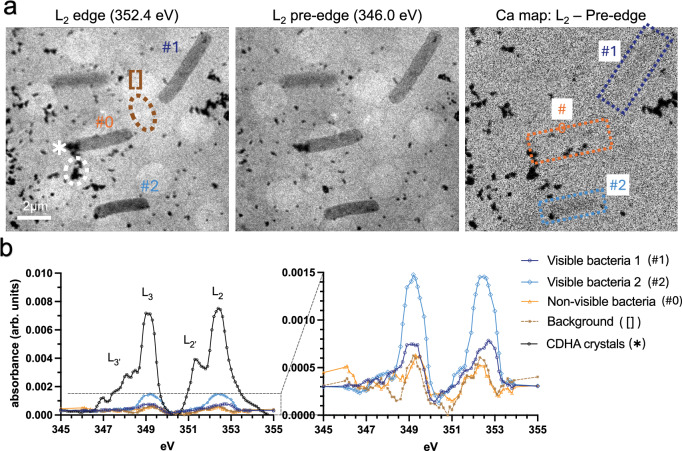
XANES spectromicroscopy at the Ca L_2,3_-edges. **a** Normalized projection image of vitrified *B. subtilis* incubated on flat CDHA, obtained at the Ca L_2_ pre-edge (346.0 eV), the L_2_-edge (352.4 eV), and the corresponding calcium map obtained by subtracting the pre-edge (346.0 eV) from the edge image (352.4 eV). **b** XANES spectra extracted from the different entities highlighted in (**a**), including the background signal, CDHA crystals, two visible bacteria, and a bacterium not visible in the Ca map. The spectra reveal distinct Ca L_2,3_ absorption intensities in consonance with the contrast in the Ca map. Images and spectra are representative of ten image stacks acquired for the flat CDHA sample. Source data is provided in the Source data file, and the XANES image stack is available on the EMPIAR public repository^61^.

Figure 6a shows a normalized Cryo-SXT projection of bacteria incubated on flat CDHA, recorded at the Ca L_2_-edge together with the corresponding calcium map obtained by subtraction of the pre-edge image (346.0 eV). In addition to the Ca-rich CDHA crystals, some bacteria appeared in the Ca map, suggesting Ca internalization. In Fig. 6b, the XANES spectra of CDHA crystals, two bacteria visible on the Ca map, a non-visible bacterium, and the background noise are displayed. The spectra were generated by averaging the absorbance values across the selected areas spanning the Ca L_2,3_-edge. As expected, the CDHA spectrum showed the characteristic doublet structure of hydroxyapatite, including the L_2_’ and L_3_’ pre-peaks, consistent with previous reports^18^, as shown in Fig. S2a of the Supplementary Information. Visible bacteria in the Ca map exhibited pronounced L_2_ and L_3_ peaks, with absorbances in consonance with the contrast in the calcium map, whereas the non-visible bacterium showed low, broad peaks indistinguishable from the background, probably corresponding to a background signal of Ca.

**Fig. 7:**
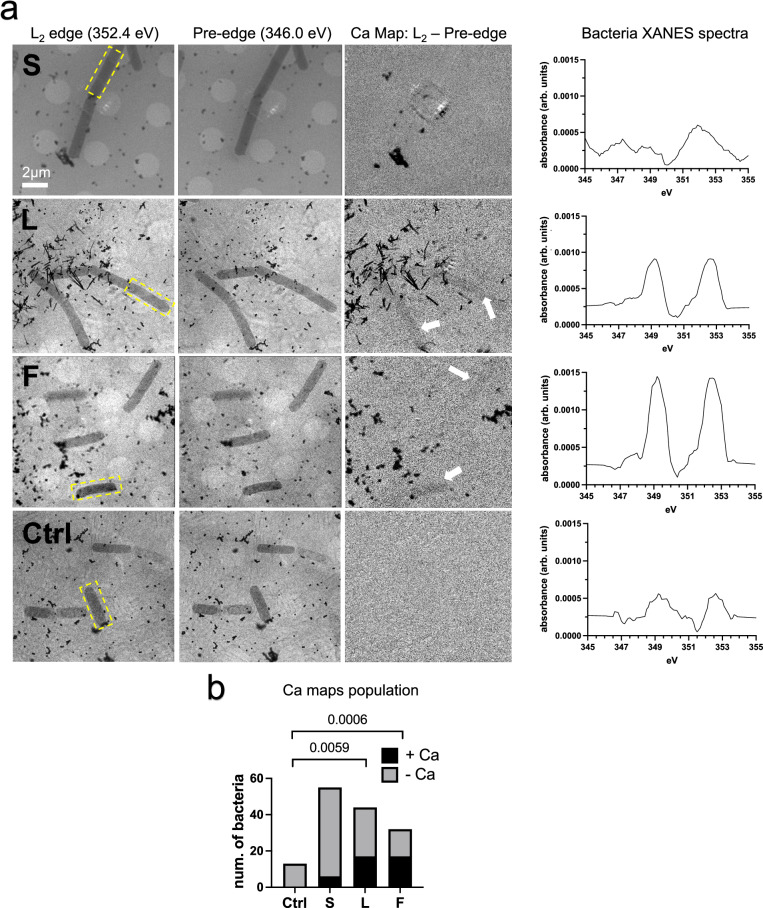
Calcium maps and XANES spectromicroscopy of bacteria incubated on CDHA substrates (S, L, F) or in the culture well (Ctrl). **a** Normalized projection images of bacteria acquired at the Ca L_2_-edge (first column), the pre-edge (second column), and Ca maps generated by subtracting the pre-edge from the Ca L_2_-edge image (third column). White arrows indicate bacteria exhibiting a Ca signal in the Ca maps. Corresponding XANES spectra at the Ca L_2,3_-edges were extracted from the cytosol of the bacteria outlined with a yellow dashed rectangle in the Ca L_2_-edge images (right graphs). **b** Quantification of the proportion of bacteria displaying Ca signal (+Ca) versus those with non-detectable signal (−Ca) across experimental groups. Statistical differences were determined by Fischer’s exact test (two-sided, *P* < 0.05). *P*-values are indicated in the graph. For XANES spectromicroscopy at the Ca L_3,2_ edges, several image stacks were acquired (small pillars, *n* = 9; large pillars, *n* = 7; flat CDHA, *n* = 10; control, *n* = 3). Source data is provided in the Source data file, and XANES image stacks are available on the EMPIAR public repository^61^.

Representative Ca maps for each condition are shown in Fig. 7a, alongside the corresponding XANES spectra of selected bacteria. No calcium-rich bacteria were visible in the Ca maps of bacteria incubated in the empty wells (Ctrl). Bacteria were rarely detected in the calcium maps of the small nanopillar samples, whereas some detached CDHA crystals were visible. In contrast, a higher number of bacteria were revealed on the Ca maps of the large CDHA nanopillars and flat CDHA groups. These findings confirm that Ca is being internalized by some bacteria on these surfaces. Notably, Ca appeared to be homogeneously distributed throughout the cytosol, and no Ca-dense particles were observed.

The XANES spectra of bacteria cytosol were compared across conditions. Similar spectra were obtained for Ca-rich bacteria incubated on L and F CDHA substrates, showing well-defined Ca L_2_ and L_3_ peaks, while no defined L_2,3_’ pre-peaks were observed. The spectra profile is consistent with calcium ions in solution, as previously reported, for instance, in CaCl_2_ solutions^19^ (Fig. S2b) and in seawater^14^, both exhibiting a low or absent L_2_’ peak. This, together with the observation that Ca maps revealed a homogeneous distribution throughout the bacterial cytosol, supports the interpretation that Ca was in soluble form. In contrast, bacteria incubated in the empty culture wells and most of those on small pillars exhibited low, broad peaks. Additional XANES spectra of representative bacteria are shown in Fig. S3 of the Supplementary Information.

The number of bacteria visible in the Ca maps was quantified relative to the total bacterial population for each group (Fig. 7b). Despite the inherently limited sample size due to technical constraints of the long image acquisition times for XANES spectra, statistically significant differences were observed with a higher percentage of calcium-rich bacteria in the F and L groups (53.13% and 38.64%, respectively).

#### Linear absorption coefficient and calcium concentration

Cryo-SXT enables quantification of the LAC, which, according to Beer–Lambert’s law, is proportional to the presence of the element responsible of X-ray absorption. At the Ca L_2_ edge (352.4 eV), Ca exhibits maximal absorbance compared to other elements. Thus, variations in LAC at this energy can be associated with differences in Ca content. While the X-ray absorbance of other elements is not negligible across the L_2,3_-edges, their contribution does not vary significantly, allowing for a semi-quantitative comparison of the Ca concentration between conditions.

**Fig. 8:**
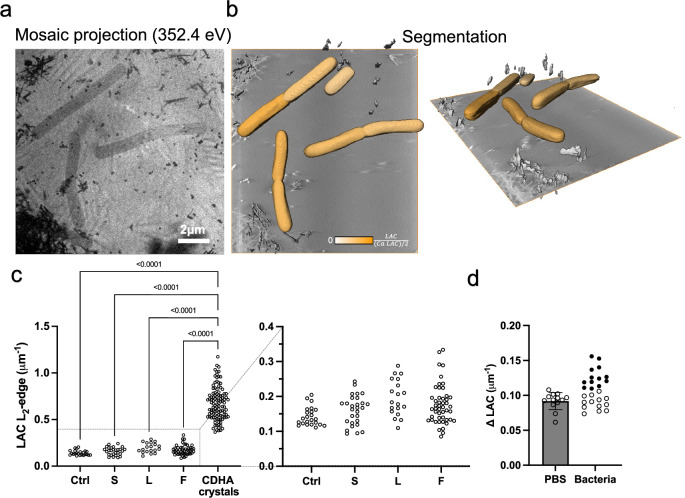
LAC analysis at the L_2_-edge between substrates and ΔLACs from the pre-edge for Ca concentration estimation. **a** Cryo-SXT projection of bacteria exposed to large topography. **b** Segmented 3D reconstructions of individual bacteria and CDHA crystals from the tomography, shown from top and tilted views. The color scale indicates relative LAC values, ranging from 0 (white) to the maximum value, set as half of the LAC of CDHA crystals (orange). CDHA crystals are shown in grey to differentiate them from bacteria. Segmentation enables the quantification of cytosolic LAC independently from the surrounding material. **c** Scatter plot showing LACs of individual bacteria and CDHA crystals, corresponding to all bacteria identified in four independent tomographies per condition. Each data point represents the mean LAC value of a single bacterium, calculated as the average voxel intensity of the segmented volume. Statistical differences were determined by a Kruskal–Wallis test followed by Dunn’s post hoc test (two-sided). **d** LAC increment (ΔLAC) between the L_3_-edge (349 eV) and the pre-edge (346 eV) for vitrified PBS and bacteria incubated on large pillars, from four independent tomographies. The bar indicates the mean and standard deviation of ΔLACs for PBS. The scatter plot displays ΔLACs of individual bacteria. Black dots represent bacteria with ΔLAC significantly higher than PBS, assessed by 1-sample t-test (*α* = 0.01). Source data are provided in the Source data file, and tomographies are available on the EMPIAR public repository^61^.

To quantify LACs, tomographies were acquired at the L_2_-edge (Fig. 8a), reconstructed in 3D, and segmented to isolate individual bacterial cells and CDHA crystals (Fig. 8b). LACs were calculated by averaging the voxel intensities within each segmented volume. Only viable bacteria were included in the analysis, as cytosolic loss results in decreased absorbance. Videos of the complete aligned tilt series can be found in the Supplementary Information (Supplementary Movies 4 and 5). The LAC values for CDHA crystals and individual bacteria across different substrates are shown in Fig. 8c. As expected, CDHA crystals exhibited significantly higher LACs than the bacterial cytosol. For bacteria incubated in the culture well (Ctrl), LAC values ranged between 0.111 and 0.205 μm^−1^. This variability likely reflects contributions from other elements. A substantial fraction of bacteria cultivated on CDHA substrates exhibited LACs comparable to the control, suggesting no additional Ca uptake. This is consistent with previous results from Ca maps and Fluo 4-AM staining. However, a distinct population subset exhibited elevated LACs, indicating increased Ca accumulation. The highest recorded LAC values followed an increasing trend across groups: control (0.205 μm^−1^), small CDHA pillars (0.243 μm^−1^), large CDHA pillars (0.288 μm^−1^), and flat CDHA (0.334 μm^−1^). This trend suggests that the amount of Ca internalized varies depending on the CDHA topography.

To quantify the intracellular Ca concentration, it is necessary to determine the LAC increment (ΔLAC) between the value at the L_3_-edge (349 eV) and that at the pre-edge (346 eV). Assuming that the absorption by other elements remains constant in this energy range, any ΔLAC can be attributed solely to Ca^20^. This was done for bacteria incubated on large pillars, as shown in Fig. 8d, where also the ΔLAC values corresponding to vitrified PBS are displayed. While some bacteria showed values of the same order as PBS, a fraction of the population showed higher values. Taking into account that the calcium concentration of PBS, determined by ICP (Fig. 3), is known (4.53 μM) and that, according to the Beer–Lambert law, there is a linear relationship between LAC values and concentration, it is possible to estimate the intracellular calcium concentration of the bacteria that had been in contact with the nanotopography. The highest measured ΔLAC was 0.156 μm^−1^, corresponding to an intracellular Ca concentration of 7.68 μM.

## Discussion

The mechanobactericidal effect of high aspect-ratio nanostructured surfaces has gained interest since their initial demonstration on insect wings^4^. These nanotopographies have been developed on various materials, and a variety of bactericidal mechanisms have been proposed, including cell wall mechanical rupture via tensile stresses, direct puncture by the sharp nanofeatures, or by stress-induced biological processes by steric impedance^21,22^. While techniques, such as SEM and FIB, have been employed to illustrate these effects^21,23^, they often require complex sample preparation processes that may introduce artefacts.

The situation becomes more complex when these nanotopographies are developed in bioactive materials, such as calcium phosphates, which are intended to simultaneously promote bone regeneration and exert local antimicrobial action. The ease of tuning the shape and size of CDHA crystals by reaction parameters has been widely exploited for the promotion of osteogenesis. In our previous work, we showed that high-aspect-ratio, needle-like CDHA nanocrystals provide a more favorable osteogenic environment, influencing not only immune cells^24^, osteoclasts^25,26^, mesenchymal stem cells^27,28^ and endothelial cells^29^ in vitro but also promoting bone formation in vivo^30–32^. In this case, ionic exchange with the surrounding environment introduces additional variables that must be considered alongside mechanical stimuli.

Here, we demonstrate the value of cryo-soft X-ray microscopy for the simultaneous characterization of bacterial interactions with bioactive substrates, both mechanically and chemically. The minimal sample handling requirements and its ability to image whole hydrated, unstained bacteria represent significant advances over more traditional approaches.

Our previous work demonstrated contact-killing effects towards Gram-negative *Pseudomonas aeruginosa* using calcium phosphates topographies^7^. Achieving mechanobactericidal effects on Gram-positive bacteria poses a greater challenge due to their thicker, more rigid bacterial wall. In this study, *B. subtilis* was used, a model Gram-positive bacterium commonly used in microbiology research and previously investigated with antimicrobial topographies^8,33–35^.

High-aspect-ratio topographies of CDHA nanopillars were synthesized by hydrolysing α-TCP under controlled temperature conditions. Increasing the hydrolysis temperature from 37 to 100 °C accelerated the hydrolysis reaction^36^, yielding taller nanopillars with greater interpillar spacing. However, crystal thickness remained unaffected (Fig. 1a). XRD analysis confirmed that hydrolysis at 37 °C (S and F samples) produced mostly CDHA (>94%), with minor unreacted α-TCP and traces of β-TCP, which is stabilized during the quenching process of α-TCP^37^. Conversely, hydrolysis at 100 °C yielded 14.8% of (β-TCP), consistent with previous findings^32,38^.

Bacterial adhesion experiments revealed increased mortality on nanostructured CDHA compared to flat controls (Fig. 2). *B. subtilis* exhibited less susceptibility compared to previous results with *P. aeruginosa* on similar topographies^7^. It is likely attributable to the thicker peptidoglycan layer of the Gram-positive bacterial wall, which confers higher structural strength. This trend was already observed on black silicon nanotopographies, which also reported a *B. subtilis* mortality lower compared to *P. aeruginosa*^34,35^. Detailed information on the performance of nanostructured topographies tested against *B. subtilis* and other Gram-positive bacterial strains is provided in Table S2 of the Supplementary Information.

Notably, FE-SEM images evidenced the presence of severely damaged bacteria on the nanostructured surfaces, and synchrotron Cryo-SXT provided very relevant information in this respect, as it allows imaging whole bacteria in the hydrated state with minimal pre-processing beyond sonication and vitrification. Because images are acquired in transmission mode, detachment of bacteria from the substrate is required. While this represents a methodological limitation for biomaterial studies, as it does not allow explicit correlation between bacterial cell wall damage and their relative spatial position with respect to the nanopillars, the recovery of bacteria by sonication enabled us examining both intact and damaged cells that had been exposed to different conditions and compare their ultrastructure and chemical speciation. Importantly, the strong agreement between metabolic and live/dead assays on attached bacteria and the CFU counts of bacteria detached by sonication (Fig. 2) confirms that the sample preparation does not affect bacterial viability or mortality, ensuring the reliability of the observed effects. Furthermore, as shown in Fig. S4, live/dead confocal imaging of bacteria detached using the same protocol revealed no significant differences in mortality between adhered and detached bacteria across all tested topographies. Cryo-SXT revealed bacteria with a low absorption contrast suggestive of cytosolic leakage and cell wall rupture on both nanostructured substrates (Fig. 5a, white arrows). Nevertheless, due to the inability to visualize bacteria while physically attached to the nanostructured topographies, it is not possible to unambiguously attribute the observed envelope damage to direct mechanical piercing by individual nanopillars. Moreover, intracellular vesicles and multivesicular bodies, as well as endospore formation, were observed. In *B. subtilis*, the formation of vesicles and multivesicular bodies is commonly associated to stress response. For instance, this process has been described as a pre-lytic remodeling response that can be triggered by cell envelope damage^39^ or by autolytic mechanisms aimed at destroying severely compromised cells or serving as a decoy to protect neighboring ones^40^. Nanopillared topographies have also been reported to induce both effects in *P. aeruginosa*^22^. Although cell envelope damage was observed in bacteria on both small and large pillars, vesiculation was detected exclusively in the S group, typically coinciding with reduced cytosolic contrast. Given the time- and resource-intensive nature of Cryo-SXT, only a limited number of cells (on the order of tens) could be analyzed, precluding statistically robust quantification. Moreover, Cryo-SXT may not detect all vesicles, particularly in cells with intact or dense cytoplasm, due to resolution (∼30 nm) and sensitivity limitations. Considering that bacterial viability was comparable on the S and L substrates, we cannot exclude the possibility that vesiculation also occurs on the latter. Notably, no correlation was found between vesiculation and elevated intracellular calcium levels, as a higher proportion of bacteria with increased calcium content was observed on the large-pillar and flat substrates.

Given the bioactivity of CDHA, ionic interactions were further investigated. Ionic exchange in CDHA is mediated by a hydrated surface layer and does not follow the rules of congruent dissolution. Upon immersion in PBS, which lacks calcium, CDHA released Ca while simultaneously uptaking P, indicating concurrent dissolution and sorption processes^41^. The latter can be associated with the positive charge of Ca-rich CDHA crystal faces, which favor phosphate adsorption from the medium. A mild acidification was also observed, a property previously described for this material^41^. Ionic exchange was most pronounced in the flat substrate, followed by the large and small topographies. The higher reactivity of the F samples is coherent with the smaller crystallite size, higher porosity, and specific surface area, as determined by Nitrogen adsorption (Supplementary Information, Table S3). This is a consequence of the preparation protocol since F samples were prepared by fabricated by direct compaction of individual CDHA nanocrystals, generating numerous interconnected nanometric channels that increase the effective surface area. In contrast, S and L samples were produced by hydrolysis of pre-compacted α-TCP, where in situ crystal growth results in interlocked CDHA structures with lower interconnected porosity. Additionally, the higher Ca release from L samples is likely attributable to their β-TCP content, which is a more soluble phase than CDHA^28,42^. P changes, independently of the condition, were not statistically significant. In the presence of bacteria, these ionic fluctuations were largely buffered, probably due to *B. subtilis* metabolic processes, known to elevate pH^43^. No microstructure degradation was observed associated with these ionic exchanges.

Cryo-soft X-ray microscopy emerged as a powerful tool to assess whether topography-induced extracellular ionic fluctuations elicited by the different substrates influence intracellular calcium levels. Ca maps revealed that *B. subtilis* can internalize calcium in amounts dependent on the Ca release profile of the substrate (Fig. 7a, c). Our findings align with previous observations by Keren-Paz et al.^43^, who reported that extracellular calcium availability led to heterogeneous calcium accumulation in *B. subtilis*, with distinct subpopulations exhibiting either low or elevated calcium levels. The accumulation of intracellular calcium in a subset of cells was proposed to generate a saturated mineral precursor microenvironment, potentially representing an early step in calcite nucleation. In our experiments, Ca-rich precipitates were not observed, likely due to insufficient incubation time^43,44^. Instead, we detected elevated calcium concentration homogeneously distributed in the cytosol of a fraction of the bacterial population, and Ca L-edge XANES spectra supported the presence of calcium in a soluble state. It is important to acknowledge that the assessment of intracellular calcium by XANES is restricted to bacteria that retain their cytosol and therefore have an intact or only slightly damaged cell wall. This limitation, however, is not exclusive to the XANES analysis; it similarly applies to the Fluo-4 AM assay. Interestingly, an excellent correlation was observed between the Fluo-4 fluorescence data and the calcium maps. The number of bacteria visible in the latter (Fig. 7b) was higher on the L and F substrates, which also exhibited the highest percentage of Fluo-4 AM-labeled cells (Fig. 4b), revealing an increase in calcium-rich bacterial cells correlated with elevated Ca release from the underlying substrate.

An advantage of cryo-SXM lies in its capacity to reveal local elemental composition using the LAC, based on Beer–Lambert’s law. This capability enabled us to estimate intracellular Ca concentration on individual bacteria (Fig. 8). Bacteria LAC values (0.168 μm^−1^, average of all conditions) were significantly lower than those of CDHA crystals (0.673 μm^−1^). The high dispersion in LAC values observed for CDHA crystals is likely due to their small dimensions, which approach the spatial resolution limit of the microscope. Comparing between conditions, while most bacteria exhibited LACs similar to the control, a distinct subpopulation showed elevated values, increasing according to the Ca release by the substrate.

A quantification of cytosolic Ca concentration was possible by determining the ΔLAC between the L_2_-edge (349 eV) and the pre-edge (346 eV), as the subtraction of the pre-edge LAC discards the contribution of other elements. The intracellular Ca concentration was estimated for bacteria incubated with the samples with large pillars (L), by linear extrapolation, using the LAC of vitrified PBS, whose concentration had been determined by ICP (4.53 μM Ca), as a reference. The highest value of cytosolic Ca concentration found was 7.68 μM, a value much lower than the Ca concentration measured in the supernatant of the L samples after 3 h of bacterial culture, c.a. 30  μM (Fig. 3a).

While these results illustrate that ionic exchange between the substrate and surrounding fluids can influence the internal concentration of *B. subtilis*, our findings indicate that this process does not directly impact its mortality. One could argue that both XANES calcium maps and Fluo4-AM assays, as well as the calcium quantification performed using LAC, do not monitor the calcium levels that may have been present in ruptured bacteria and that could hypothetically have contributed to their rupture. However, even assuming that calcium concentration analysis is limited to bacteria with intact cell walls, if an increase in intracellular calcium concentration were a trigger for cell death, one would expect a correlation between the substrates showing higher intracellular calcium levels in the alive bacteria and those exhibiting greater mortality, which was not observed in this case. In fact, the substrate exhibiting the highest ionic exchange capacity, the flat substrate (F), did not result in increased mortality rates compared to the control (Fig. 2b), suggesting that ionic exchange alone is not a determining factor in bacterial viability under the conditions tested. This observation is consistent with the known halotolerance of *B. subtilis*^44,45^, which enables adaptation to environments with elevated salinity^46^.

Overall, this study highlights the dual role of substrate topography and chemistry in influencing bacterial behaviour and viability. While the halotolerance of *B. subtilis* prevented mortality linked to ionic uptake in this case, enhancing both the mechanical and chemical components could further improve the antimicrobial performance of the substrates. Refining nanotopographical parameters, such as nanopillar sharpness, may increase mechanobactericidal efficiency. In parallel, surface functionalization with antimicrobial peptides offers a complementary strategy^47^. Owing to their cationic nature, antimicrobial peptides strongly interact with negatively charged bacterial membranes, not only amplifying the mechanical damage induced by nanotopographies but also introducing additional electrostatic and biochemical routes for cell envelope disruption. This combined action can yield multimodal bactericidal surfaces with superior efficacy^48^.

Similarly, doping CaP-based topographies with antimicrobial ions could reinforce bactericidal effects through synergy between ionic toxicity and mechanical stress^49^. Although calcium showed no toxicity toward *B. subtilis* in this study, the observed ionic exchange between bacterial cells and the substrate reveals additional interaction pathways beyond mechanical damage. As metal ion accumulation is often central to toxicity, through membrane destabilization, intracellular interference, or ROS generation, leveraging these synergistic mechanisms may further magnify antimicrobial performance^50^.

In this context, cryo-SXM emerges as a powerful, label-free tool for investigating bacteria–substrate interactions, providing high-resolution insight into both mechanical and ionic exchange processes. By combining cryo-SXT with cryo-XANES, we achieved quantitative intracellular characterization of ionic Ca^2+^ in bacteria at the single-cell level. This integrative approach, linking imaging, spectroscopy, and quantitative analysis, allows exploring elemental dynamics in microorganisms and could be extended to other biologically relevant elements within the water window^51^. This dual analytical capability is particularly valuable for characterizing multifunctional biomaterials with concurrent bioactive and bactericidal properties.

## Methods

### Materials fabrication

#### Synthesis of α-tricalcium phosphate (TCP)

α-TCP was synthesized by mixing calcium hydrogen phosphate (CaHPO_4_, Sigma-Aldrich, St. Louis, USA) and calcium carbonate (CaCO_3_, Sigma-Aldrich, St. Louis, USA) in a 1:2 molar ratio. The mixture was sintered for 15.5 h at 1400 °C and subsequently quenched in air at room temperature. The resulting α-TCP was ground into powder using a planetary ball mill. Milling consisted of three stages: 40 min at 450 rpm and 60 min at 500 rpm with using 10 agate balls (*ø* = 30 mm), and a final 60 min at 500 rpm with 100 agate balls (*ø* = 10 mm).

#### Fabrication of CDHA topographies

Fifty milligrams of α-TCP powder were compacted into 5 mm-diameter discs by applying a uniaxial pressure of 2 tons for 2 min. Two nanostructured topographies of CDHA were generated by hydrolyzing α-TCP under distinct conditions:

- Large CDHA pillars (L): hydrolysis in 300 mL of distilled water preheated to 100 °C, 1 atm, for 1 hour.
- Small CDHA pillars (S): hydrolysis in 300 mL of distilled water at 37 °C for 7 days.

Flat CDHA discs (coded as F) were prepared by first hydrolysing 2 g of α-TCP powder in 100 mL of distilled water at 37 °C for 7 days. The CDHA powder obtained was dried and compacted using the same protocol described above. As a control group, empty tissue culture polystyrene wells were used, except for the live/dead experiments, where 5-mm glass coverslips were used to allow FE-SEM imaging.

### Physico-chemical characterization

#### Topography microstructure analysis

The topographies surface and cross-section were examined using a field emission scanning electron microscope (FE-SEM, Carl Zeiss AG - EVO® 50 Series, Zeiss, Germany). Surface images were acquired at an accelerating voltage of 5 kV, while cross-section images were obtained at 7.5 kV. To minimize charging effects, samples were coated by carbon evaporation. Image analysis was performed using Fiji (Image J v.2.9.0/1.53t) to quantify interpillar spacing, nanopillar thickness, height, density, and orientation with respect the surface normal. Three different micrographs were analysed per condition.

#### Transmission electron microscopy (TEM)

The nanostructured topographies were sonicated in ethanol for 30 s to detach individual nanopillars. Twenty microliters of the resulting suspension were deposited onto copper grids with a holey carbon film and air-dried. The nanopillars were imaged in a transmission electron microscope operated at 120 kV accelerating voltage. Structural information was obtained through SAED. The SAED patterns were indexed according to the structural parameters provided in the inorganic crystal structure database (ICSD), ICSD-050656, for monoclinic apatite.

#### Phase composition and crystallite size analysis

The crystallographic phases were identified by XRD from three replicates analyzed per condition. The diffractometer was equipped with a Cu Kα X-ray tube and a primary monochromator. It operated at 40 kV and 40 mA, with data collected in 0.02° steps over 10–60° and a counting time of 2 s per step. Phase quantification was conducted via Rietveld refinement using the Profex software (Profex 4.3.5)^52^. Crystalline models for CDHA, α-TCP, and β-TCP were retrieved from the International Centre for Diffraction Data (ICDD), ICDD PDF 01-086-1201, ICDD PDF 00-029-0359, and ICDD PDF 00-003-0681, respectively. Crystallite size (X_hkl_) was estimated from the (211) peak of HA using the Scherrer’s equation (Eq. 1):

Eq. 1 $${{{ X}}}_{{{ h}}{{kl}}}={{ K}} * \frac{{{\lambda }}}{{{{\beta }}}_{{{1}}/{{2}}} * {{\cos }}({{\theta }})}$$

Where *λ* is the wavelength of monochromatic X-ray beam (0.15406 nm for Cu Kα radiation), *β*_1/2_ is the full width at half maximum for the peak under study (rad), *θ* is the diffraction angle (^o^), and K is a constant varying with crystal habit and here set to the typical value of 0.89. “Peak profiles were deconvoluted using a pseudo-Voigt function (Fig. S1)”.

#### Specific surface area and porosity

The specific surface area (SSA) and porosity were determined by nitrogen adsorption. Twenty discs of each group were degassed at 10 mm Hg at 100 °C overnight. The adsorption and desorption isotherms were recorded. The SSA was determined using the Brunauer–Emmett–Teller (BET) method.

#### Antibacterial activity

*B. subtilis* (CCUG 10780, Culture Collection University of Gothenburg, Sweden) was cultured on Brain infusion agar plates (BHI, Scharlau, 02-599-500 with Agar bacteriological, Scharlau, 07-004-500) prepared following the manufacturer’s instructions. For bacterial inocula, one colony was suspended in BHI and grown overnight at 37 °C until the exponential growth phase. The culture was then centrifuged at 3500 × *g* for 5 min, and the old supernatant was discarded. Then, the cells were resuspended in phosphate-buffered saline (PBS tablets, Gibco®, ref. 18912-014, lot 2153555) and adjusted to 0.25 optical density (OD_600_). PBS was chosen to minimize bacterial growth throughout the experiment. The CDHA pellets were sterilized by autoclaving in distilled water at 121 °C for 20 min. Discs were transferred to a sterile 48-well plate and incubated with 0.7 mL of the bacterial suspension for 3 h at 37 °C. After incubation, non-adherent bacteria were rinsed by immersing each sample thrice in sterile PBS. The samples were then stained using LIVE/DEAD® BacLight™ Bacterial Viability Kit (Invitrogen, Cat. No. L7012, USA). The staining solution was prepared by diluting 2.5 μL of the mixture of 1.67 mM SYTO9 and 18.3 mM propidium iodide (PI) per 1 mL of PBS. The samples were incubated in 350 μL of diluted dye for 10 min at room temperature, protected from light, washed three times with PBS, and imaged in a confocal laser microscope. Zen Blue 2.3 (Zeiss Microscopy) software was employed for image acquisition and image analysis. The analysis criteria used considered green cells (SYTO 9) as viable, whereas cells fluorescing red (PI) or displaying both fluorophores colocalized were considered non-viable. Confocal microscopy experiments were performed independently on three separate days (*n* = 3), and four distinct images were taken and quantified per condition (*n* = 12).

#### SEM bacteria preparation

Following confocal imaging, bacteria were fixed with 500 μL of 2.5% glutaraldehyde in PBS overnight at 4 ˚C, washed with PBS three times, and dehydrated through a graded series of aqueous ethanol solutions (50%, 70%, 90%, 96%, and 100% twice, Ethanol Absolute Pure, 141086, PanReac Applichem), each step lasting for 10 min. Following ethanol dehydration, a graded series of HMDS (1,1,1,3,3,3-hexamethyldisilazane 98%, Thermoscientific, Cat. No.: 120585000) with ethanol solutions (33.33%, 50%, 66,67%, and 100% three times) were applied for 15 min each. Dried samples were subsequently coated with carbon sputter to minimize charging effects. Images were obtained on a Tescan Amber field-emission microscope (Tescan, Brno, Czech Republic) operated at an accelerating voltage of 5 kV and were acquired at zenithal and oblique views (55° tilt).

#### Metabolic activity assay

Metabolic activity of adherent bacteria was quantified using the BacTiter-Glo™ assay (Promega). Following the adhesion experiment, CDHA discs were incubated for 5 min in a 1:1 mixture of phosphate-buffered saline (PBS) and BacTiter-Glo™ reagent. The resulting luminescent solution from each disc was transferred to an opaque white 96-well plate (Nunc™ MicroWell™ Flat-Bottom, Thermo Fisher Scientific, cat. no. 136101), and luminescence was recorded immediately using a plate reader (Tecan Infinite® M Plex multimode microplate reader, Tecan Group Ltd.). Experiments were performed independently on three separate days (*n* = 3), and 3 technical replicates were used per condition (*n* = 9).

#### Colony-forming unit (CFU) enumeration

For CFU determination, CDHA discs were first washed three times with PBS to remove non-adherent bacteria. Bacteria were detached using the same sonication procedure used for cryo-SXT sample preparation to ensure reproducibility of the results. The protocol is described in detail in that section and illustrated in Fig. S5. The resulting suspension was diluted by transferring 20 µL into 180 µL of PBS. Serial tenfold dilutions of these suspensions were plated on Mueller–Hinton agar (Scharlau, cat. no. 02-136-500) using the Miles and Misra method and incubated overnight at room temperature. Colonies were enumerated the following day. Mueller–Hinton agar was prepared according to the manufacturer’s instructions.

#### Fluo-4 AM calcium internalization assessment

Intracellular calcium accumulation in *B. subtilis* was assessed using Fluo-4 AM (ThermoFisher Scientific, F14201), a calcium-sensitive dye originally developed for eukaryotic cells but also validated for Gram-positive bacteria^53^. Incubation conditions were optimized for *B. subtilis* and determined by comparing different timepoints (30 min, 45 min, 1 h and 2 h) and temperatures (37 °C and room temperature). To normalize data with the total population, CellBrite™ Fix 555 Membrane Labeling Kit (Biotum Inc., #30088) was used. Both dyes were prepared by diluting 1 μL of reagent per 1 mL of PBS. In the final protocol, the substrates were incubated for 30 min with 300 μL of Fluo-4 AM solution at room temperature, protected from light. After adding 0.3 μL of CellBrite™ solution, they were incubated for 15 additional minutes. Subsequently, the samples were imaged using confocal microscopy and analysed using Zen Blue 2.3.

#### Material reactivity

The reactivity of the different substrates was assessed by measuring the Ca and P concentration in the surrounding medium by ICP-optical emission spectroscopy. The substrates (*n* = 6) were incubated in 48-well plates for 3 h at 37 °C with 0.7 mL of PBS, with or without bacteria (OD_600_ = 0.25). Empty culture wells served as a control condition. After incubation, the supernatants were filtered through a 0.22 μm membrane (Millex-GP PES, Millipore). For ICP analysis, 200 μL of the filtrate weres diluted in 3.8 mL of 2% nitric acid (Nitric Acid 69% TMA, HIPERPUR, Panreac). Calibration was performed using five standard dilutions prepared from certified solutions (Inorganic Ventures) traceable to NIST. The remaining supernatant was used to measure the pH with a selective electrode.

### Synchrotron cryo-soft X-ray microscopy (cryo-SXM) experiments

All cryo-SXM experiments were conducted at the MISTRAL beamline of the ALBA Synchrotron Light Source (Barcelona, Spain). This beamline utilizes photons extracted from a bending magnet source, focalizing them onto the sample through a capillary condenser in front of the monochromator exit slit. Located following the sample, a Fresnel zone plate with an outermost zone width of 40 nm serves as the objective lens, generating a magnified image on a direct illumination CCD detector positioned a few meters from the sample^12,54^.

#### Sample preparation for cryo-SXT

An adhesion experiment was performed as previously described, by incubating three replicates per condition and three empty wells as controls for 3 h. Following incubation, the discs were washed three times with PBS to remove non-adherent bacteria. To obtain a concentrated bacterial suspension suitable for cryo-SXR grid preparation (OD_600_ > 0.3), each disc was placed face-down onto a 40 µL drop of PBS placed on the inner surface of a 24-well plate lid. The lid was then floated on the water surface of an ultrasonic bath (Sonorex Digitec DT 52, Bandelin; nominal power 60 W), and sonicated for 30 s (Fig. S5). Ten microliters of the obtained bacterial suspensions were mixed with 1 μL of 100 nm gold fiducial markers (GC100, BBI solutions) and deposited onto the carbon side of Holey carbon-coated Quantifoil® R2/2 G200F1 finder grids (Quantifoil Micro Tools, Groβlobichau, Germany), which were previously glow-discharged using a Leica ACE200 to favor bacteria attachment to the grid. Excess liquid was blotted, and the grids were plunge-frozen into liquid ethane using an automatic plunge freezer for the bare grid technique. Three grids per condition were prepared and pre-screened using a cryo-visible light microscope equipped with a cryo-stage. The samples were stored in liquid nitrogen and kept under cryogenic conditions during data collection.

#### Cryo-soft X-ray tomography

Cryo-SXT images were acquired at multiple photon energies. A first tilt series of images for tomographic reconstruction was acquired at 520 eV to maximize contrast of carbon-rich structures, facilitating optimal visualization of bacteria ultrastructure. Each projection was acquired with an exposure time of 2–10 s, using an angular step of 1° on a 141° angular range. To assess local calcium distribution, additional tilt series of images were acquired at the Ca L_2_ and L_3_ edges (352.4 and 349 eV, respectively), and at the pre-edge (346 eV) (exposure time 2–10 s, angular step 1°, and angular ranges of 101° at 352.4 eV and 121° at 349.6 and 346 eV). The effective voxel size of reconstructed tomograms was 13 nm. No signs of radiation damage were observed under these acquisition conditions.

The LAC was determined from the 3D reconstructions at the Ca L_2_-edge, based on the Beer–Lambert law^55^ (Eq. 2), which relates the attenuation of radiation to the properties of the material through which the light is traveling.

Eq. 2 $$I={I}_{0}{e}^{\int {\mu }_{l}\left(z\right){dz}}$$

Where ${\mu }_{l}$ stands for the LAC, *I*_0_ to the incoming flux, and z to the thickness of the material. The 3D reconstruction was performed following this law in the rearranged form (Eq. 3):

Eq. 3 $$-{ln}\left(\frac{I}{{I}_{0}}\right)=\int {\mu }_{l}\left(z\right){dz}={\mu }_{l}{{\cdot }}z={Absorbance}$$

Each projection of the tilt series was normalized using flat-field, accounting for different exposure times and the slight decrease in beam current during acquisition. Wiener deconvolution was applied to enhance image quality, considering the experimental impulse response of the optical system^11^. Subsequently, the Napierian logarithm was operated to each image. The resulting stacks were loaded into IMOD software^56^ for individual projections alignment using Au fiducials markers to define a common tilt-axis. Usually, this operation reduces the effective field of view by 10 to 15%. Aligned stacks were reconstructed using algebraic reconstruction techniques (ART)^57^. 3D reconstructed tomographies were segmented using Amira 3D software (Thermo Fisher Scientific, Waltham, MA, USA). LAC values were calculated by averaging voxel intensities within the segmented region and dividing by the voxel depth (0.013 μm).

The quantification of the intracellular calcium concentration was based on the increase in LAC (ΔLAC) between the pre-edge (346 eV) and the L_3_-edge (349.6 eV). This increase can be attributed solely to the contribution of calcium contribution thereby eliminating the contribution of other elements. ΔLAC was calculated by subtracting the tomographic reconstruction between both energies before reconstructing it. From the determination of the LAC value of the vitrified PBS, since its corresponding concentration was known, as it had been measured by ICP (Fig. 3a), it was possible to calculate the cytosolic calcium concentration by applying the linear relationship established by the Beer–Lambert law.

#### Cryo-soft X-ray spectroscopy and calcium maps

X-ray absorption near edge structure (XANES) spectra of calcium were scanned from 340 to 361 eV, covering the Ca L_3,2_ edges. Spectra were extracted from 2D image stacks, allowing acquisition pixel-by-pixel of absorption spectra over a 13 × 13 µm field of view^12^. During stack acquisition, the monochromator slits gaps were kept constant for each image. The energy step was set to 0.5 eV in the pre-edge (340–346 eV) and post-edge (354–360 eV) regions and 0.1 eV elsewhere. Each image was captured with a 6 s exposure time and an effective pixel size of 13 nm. Each image in the resulting stack thus represents transmitted intensity at a specific photon energy. Post-processing involved normalizing the stack of images by division with the corresponding flat-field image, and subsequently aligned with the IMOD software^56^. Ca maps were generated by subtracting the transmission image acquired at the pre-edge, to that at the Ca L_2_ edge.

#### XANES spectra

Bacteria and CDHA crystals were chosen as regions of interest (ROIs) in the calcium maps. ROIs were manually selected within the scanned area of the aligned 2D image stacks. Absorbance spectra were obtained by plotting the average ROI signal on each image across all energy steps. Due to the beam time availability constraints, no optimization experiments were performed to reduce beam damage. However, only few image stacks displayed radiation damage and were excluded from the analysis. FIJI ImageJ v2.9.0/1.53t was used for data extraction, and spectra were processed and plotted using a custom Python 3 script, provided as Supplementary Information, and available on the Zenodo repository^58^.

#### Statistical analysis

The normality of the data was assessed before analyses. Statistical significance was determined using either 1-sample t-test, a Kruskal–Wallis test, a one-way or two-way ANOVA, and a mixed-effects model for CFUs counting, as specified in each Figure legend. Statistically significant differences were considered for two-tailed *P* < 0.05, and *P* < 0.01 for ΔLAC. Statistical analyses were performed in GraphPad Prism 10.2.3 and a mixed-effects model on Minitab 19.

### Reporting summary

Further information on research design is available in the Nature Portfolio Reporting Summary linked to this article.

## Supplementary information

Supplementary Information

Description of Additional Supplementary File

Supplementary Movie 1

Supplementary Movie 2

Supplementary Movie 3

Supplementary Movie 4

Supplementary Movie 5

Reporting Summary

Transparent Peer Review File

## Source data

Source data 1

## Data Availability

The Source data underlying Table 1, Figs. 1b–g, 2b, d, 3, 4b, 6b, 7, 8c, d, and S3,S4 are provided as a Source data file. Cryo-SXT raw data shown in Figs. 5, 6a, 7a, 8a, b, and S3 were generated at ALBA synchrotron BL09 Mistral Beamline. The normalized tilt series, reconstructed tomographies, XANES image stacks and calcium maps data have been deposited in the Electron Microscopy Public Image Archive (EMPIAR) under accession codes EMPIAR-13280 (.hdf5 files are normalized tilt series;.mrc or ended in “3DS30” are reconstructed tomographies; archives including “lnWOFF” or similar text are for LAC quantification; Archives including “XANES” text are for spectra acquisition); confocal microscopy datasets are deposited in the ZENODO repository at the following links: Fig. 2 (10.5281/zenodo.19135539), Fig. 4 (10.5281/zenodo.19135331), Fig. S4(10.5281/zenodo.19135718). Source data are provided with this paper.

## Code availability

The code used for processing XANES spectra is provided in the Supplementary Information and is publicly available on ZENODO, 10.5281/zenodo.17297365, together with detailed instructions for execution.
