# Supplementary material for: Synchrotron cryo-soft X-ray microscopy reveals bacterial interactions with nanostructured calcium phosphate substrates
Source: Nat Commun. 2026 Jul 13;17:8626. doi: 10.1038/s41467-026-75323-y (PMC13487168; doi:10.1038/s41467-026-75323-y)
Supplement: Supplementary file 1 [file 41467_2026_75323_MOESM1_ESM.pdf]

Supplementary Information - Synchrotron cryo-soft X-ray microscopy reveals bacterial interactions with nanostructured calcium phosphate substrates

Marc Iglesias-Fernandez[1,2], Ana Joaquina Pérez-Berná[3], Eva Pereiro[3], Maria-Pau Ginebra[1,2,4,5,*], Montserrat Espanol[1,2,4,*]

[1] Biomaterials, Biomechanics and Tissue Engineering Group, Department of Materials Science and Engineering, Universitat Politècnica de Catalunya - BarcelonaTech (UPC), Av. Eduard Maristany, 16, 08019 Barcelona, Spain.

[2]Barcelona Research Centre in Multiscale Science and Engineering, Universitat Politècnica de Catalunya - BarcelonaTech (UPC), Av. Eduard Maristany, 16, 08019 Barcelona, Spain

[3]MISTRAL Beamline Experiments Division, ALBA Synchrotron Light Source, 08290 Barcelona, Spain

[4]CIBER de Bioingeniería, Biomateriales y Nanomedicina (CIBER BBN), Instituto de Salud Carlos III, Spain

[5]Institute for Bioengineering of Catalonia (IBEC), Barcelona Institute of Science and Technology, Baldiri Reixach 10-12, 08028 Barcelona, Spain

Corresponding authors:
maria.pau.ginebra@upc.edu
montserrat.espanol@upc.edu

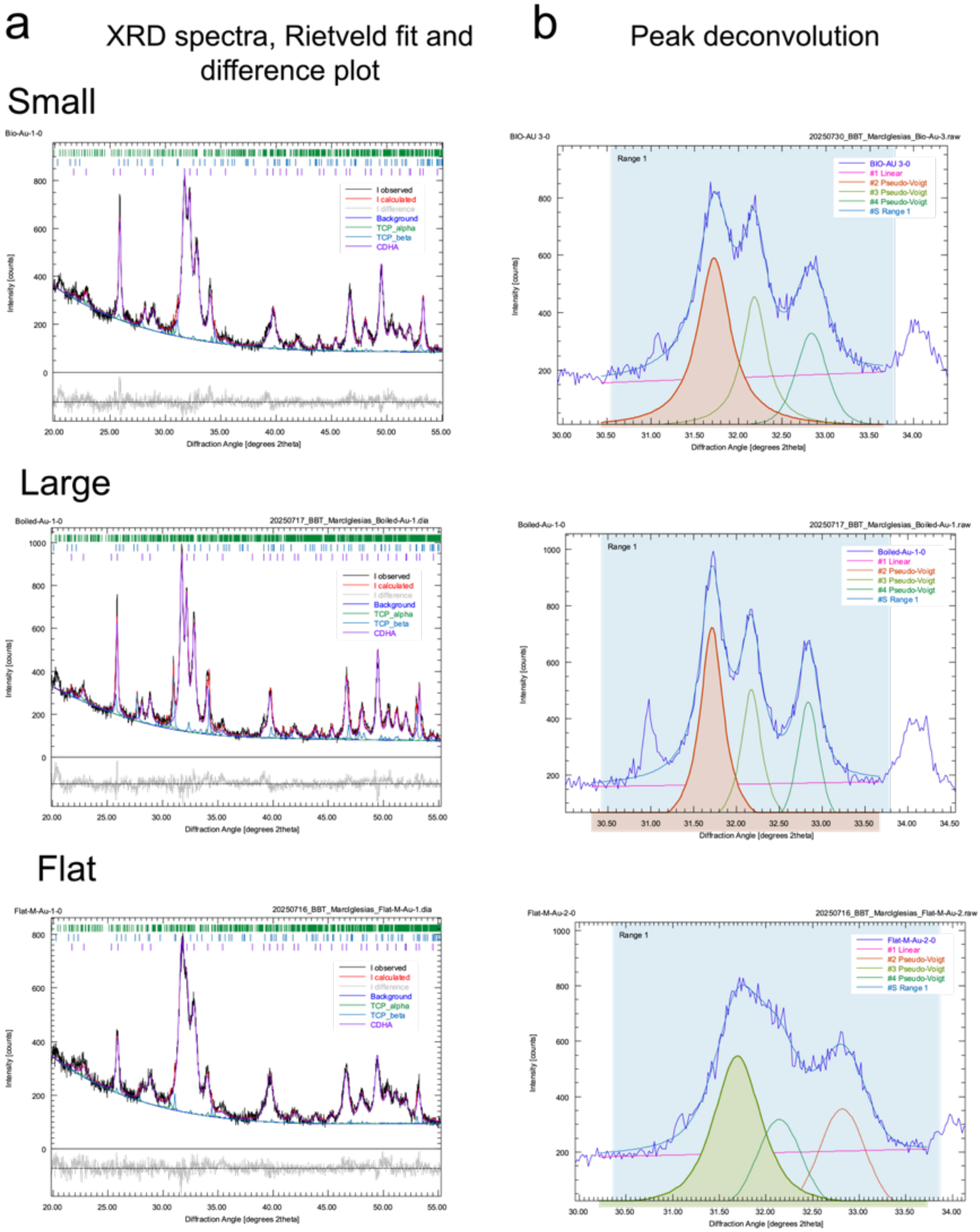

Figure S1. Representative plots of the Rietveld fit, difference plot and peak deconvolution. The shadowed peak was used for the estimation of crystallite size.

Table S1. Phase quantification and crystallite size of the Small, Large, and Flat topographies. *Reported parameters include phase wt.%, estimated standard deviation (ESD), Rietveld fitting quality indicators: weighted profile R-factor (R_{wp}) and expected R-factor (R_{exp}), from which χ^2 and the goodness of fit (GOF) are derived. Crystallite size was evaluated from the broadening of the (211) peak using the Scherrer equation. All data were above the limit of quantification (LOQ > 4·ESD) and the limit of detection (LOD > 2·ESD).*

		**Phase quantification**		**Rietveldt fitting**				**Crystallite size**
**Topography**	Phase	Phase (wt. %)	ESD (%)	Rwp	Rexp	χ^2	GoF	X_{hkl} (nm)
	alpha	2.2	0.3	6.820	6.003	1.291	1.136	36.5 ± 1.7
**Small**	β-TCP	3.7	0.4					
	**CDHA**	94.1	0.4					
	alpha	3.5	0.3	7.313	5.783	1.602	1.265	48.6 ± 2.4
**Large**	beta	14.8	0.4					
	**CDHA**	81.8	0.5					
	alpha	2.1	0.4	6.640	5.900	1.266	1.125	25.7 ± 1.7
**Flat**	beta	2.5	0.3					
	**CDHA**	95.4	0.4					

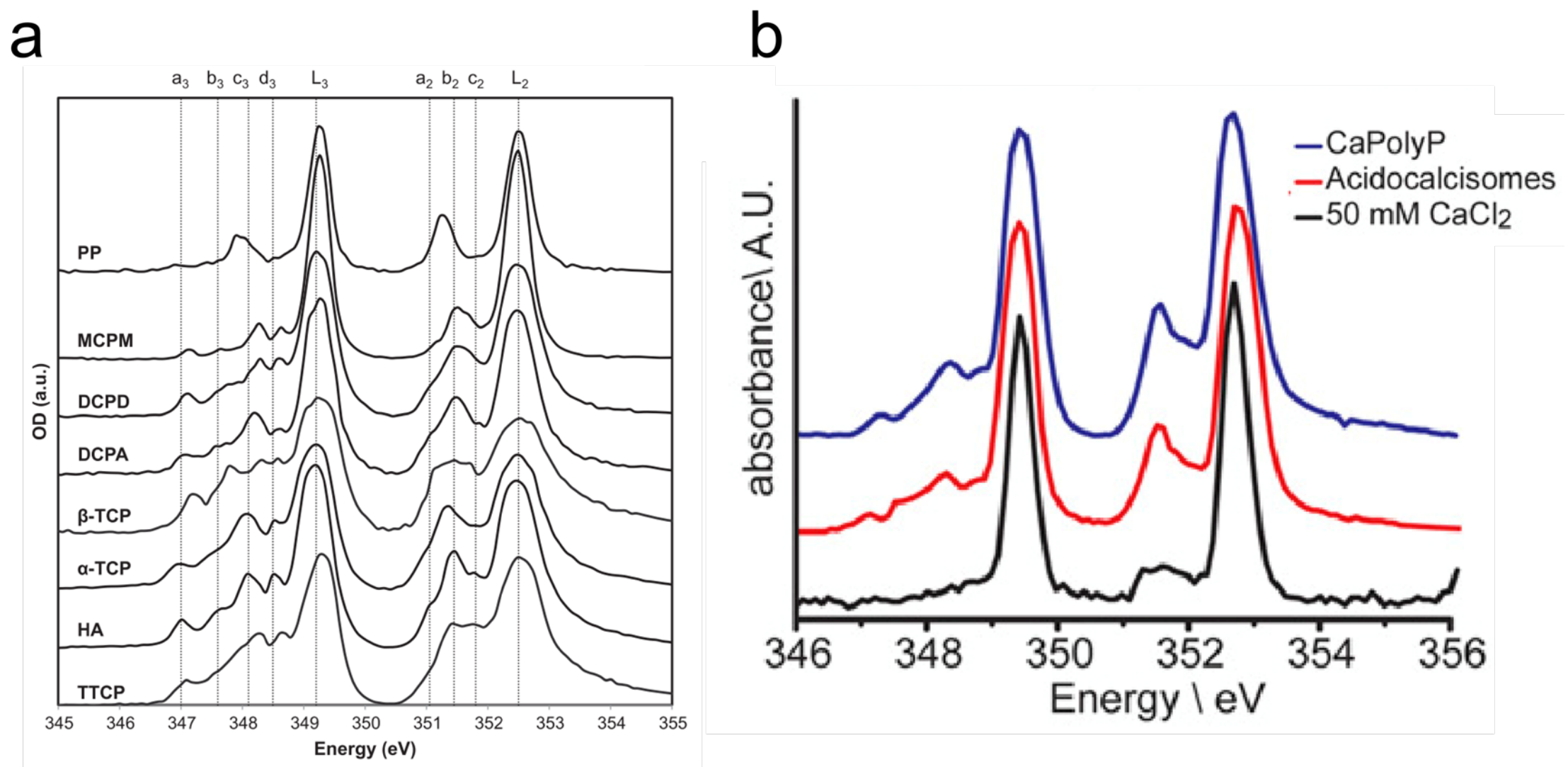

***Figure S2**. a) XANES spectra of the Ca $L_{2,3}$-edges of several Ca-phosphate reference compounds, including hydroxyapatite (HA), polyphosphates (PP), monocalcium phosphate monohydrate (MCPM), dicalcium phosphate dihydrate (DCPD), dicalcium phosphate anhydrous (DCPA), beta tricalcium phosphate (β-TCP), alpha tricalcium phosphate (α-TCP) and tetracalcium phosphate (TTCP). Vertical dotted lines correspond to the peak energies of HA. From J. Cosmidis, et a. Acta Biomater. 12, 260–269 (2015). b) Ca $L_{2,3}$ XANES spectra of calcium polyphosphates, acidocalciosimes of C. reinhardtii and a $CaCl_2$ 50 mM solution in water. From A. Gal, et al., Proc. Natl. Acad. Sci. U. S. A. 115, 11000–11005 (2018).*

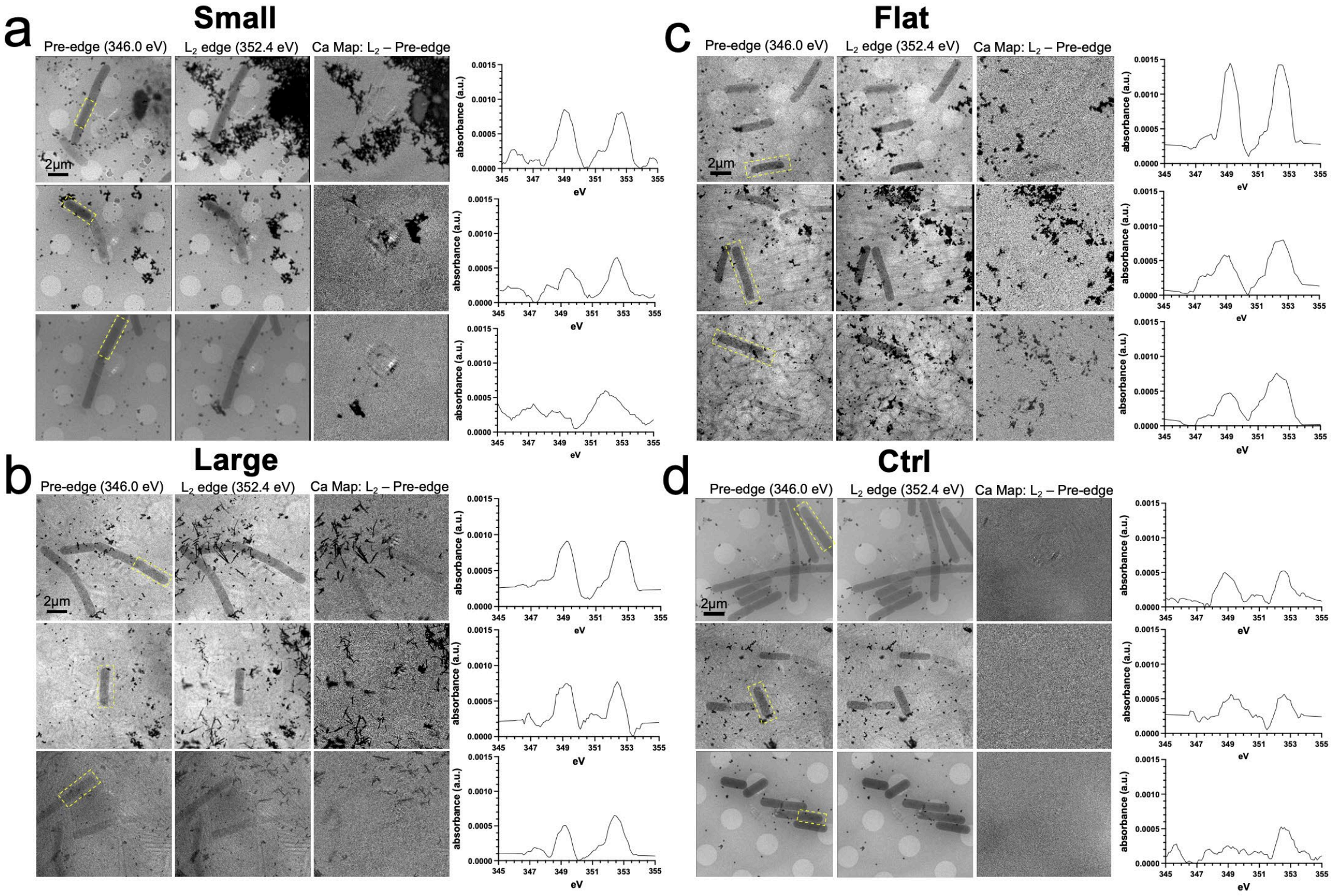

Figure S3. Calcium maps and XANES spectromicroscopy of bacteria incubated on CDHA substrates (S, L, F) or in the culture well (Ctrl). *Representative images and XANES spectra of B. subtilis incubated for 3 h on (a) Small, (b) Large, (c) Flat CDHA substrates, and (d) in the culture well (Ctrl). Normalized projection images of bacteria acquired at the Ca pre-edge (first column),* L_2*-edge (second column), and Ca maps generated by subtracting the pre-edge from the Ca* L_2*-edge image (third column). Corresponding XANES spectra at the Ca* $L_{2,3}$*-edges were extracted from the cytosol of the bacteria outlined with a yellow dashed rectangle in the Ca pre-edge images. For each condition, three spectra are shown from different cells identified in the Ca maps. The plots illustrate variability among cells and confirm that spectra from Large and Flat conditions exhibit characteristic Ca* $L_{2,3}$ *edge peaks, while signals from Small and Control conditions are weaker or absent. XANES image stacks are available on Zenodo public repository.*[1]

XANES acquisition requires significantly longer exposure times to cover 20 eV in 0.1 eV steps than single-energy tomographic imaging, which limited the number of cells that could be analyzed. Consequently, XANES data are presented as proof of concept for Ca speciation, whereas LAC quantification was used to compare intracellular Ca concentrations across conditions and populations. This correlation between LAC and XANES can be assumed because, in samples with similar thickness, such as the bacterial cells in this case, the LAC value is directly related to the height of the Ca L_2 edge peak. While the energy position of this absorption edge is fixed for Ca, its intensity scales with concentration. Thus, the modest increases in peak height observed in most XANES spectra (slightly above control values, ~0.0005 a.u.) are consistent with the small differences detected in LAC quantification (Fig. 8 of the manuscript).

Table S2. Summary of reported bactericidal efficacy of nanostructured surfaces against Gram-positive bacterial strains.

**Bacteria**	**Material**	**Incubation time**	**Reported mortality / reduction**	**Reference**
Bacillus subtilis	Black silicon	3 h	~60% mortality	[2]
Bacillus subtilis	Dragonfly wings (natural)	3 h	~50% lower than *P. aeruginosa* mortality	[3]
Bacillus subtilis	CDHA (this work)	3 h	59% mortality	–
Lactobacillus spp. (8-strain coculture)	Gecko skin replicas (chitosan, alginate, silk fibroin)	48 h – 7 days	Up to 95% (qualitative SEM analysis)	[4]
Staphylococcus aureus	Black silicon	18 h	~92% mortality	[2]
Staphylococcus aureus	Ormostamp polymer	2 h	97% mortality	[5]
Staphylococcus aureus	Silicon	3 h	86% mortality (also killed eukaryotic cells)	[6]
Staphylococcus aureus	Aluminium	24 h	Log reduction ~2.7	[7]
Staphylococcus aureus	Gold nanospikes	24 h	Log reduction ~3.7	[8]
Staphylococcus aureus	PMMA topographies	7 h	60% mortality	[9]
Staphylococcus aureus	Polyvinylidene	18 h	75% mortality	[10]
Staphylococcus aureus	Silicon	8 h	50% mortality	[11]
Staphylococcus aureus	Titanium	90 min	70% mortality	[12]
Staphylococcus aureus	Titanium	18 h	65% mortality	[13]
Staphylococcus aureus	Titanium	1 h	none	[14]
Staphylococcus aureus	Titanium	3 h	Real-Time Glo: 60% Bactiter Glo < 10%	[15]
Staphylococcus aureus	Titanium	2 h	~45%	[16]
Staphylococcus aureus	Titanium Oxide	3 h	~50%	[17]
Staphylococcus aureus	Titanium Oxide	18 h	19,8%	[18]
Staphylococcus aureus	Caloptery haemorrhoidalis and	3 h	26% mortality	[19]

	damselfly wings (natural)			
Staphylococcus aureus	Block copolymer (polystyrene-block-poly(2-vinylpyridine))	3 h	~20% mortality	20
Staphylococcus aureus	Titanium oxide	1 h	No mortality	21
Staphylococcus aureus	Titanium oxide	2 h	< 20% mortality	22
Staphylococcus aureus	Black silicon	3 h	65% mortality	23

The most frequently studied Gram-positive strains in this context include *S. aureus* and *Lactobacillus spp*.

For *B. subtilis*, mortality rates of approximately 60% have been reported after 3 h of incubation on black silicon nanotopographies.[2] Another study, using natural dragonfly wings, compared bactericidal activity across different wing types, noting that *B. subtilis* mortality was roughly 50% lower than that observed for *P. aeruginosa*.[3] In a previous work with CaP nanotopographies, we observed ~86% mortality for *P. aeruginosa* under similar conditions.[24] Therefore, the ~59% mortality obtained in the present study for *B. subtilis* is consistent with values reported in the literature.

For *Lactobacillus spp*., a coculture of eight oral strains was tested on bioinspired Gecko skin replicas made from chitosan, alginate, and silk fibroin. Mortality rates of up to 95% were reported after 48 h to 7 days of incubation, based on qualitative SEM image analysis.[4] However, the long incubation periods and qualitative assessment methods limit direct comparison with our 3 h quantitative assays.

For *S. aureus*, numerous studies have explored a wide range of synthetic and natural nanotopographies. High mortality rates have been reported in certain cases, such as 97% after 2 h on Ormostamp polymer[5] and ~92% after 18 h on black silicon,[2]. In general, nanotopographies fabricated on black silicon have shown strong bactericidal performance against *S. aureus*[6,23]. Other materials, including aluminium,[7] titanium,[12,13] gold,[8] and polymers such as PMMA,[9] or Polyvinylidene[10], have also yielded mortality rates exceeding 60%. Nonetheless, several studies have reported more modest reductions (< 50%) after incubation across different materials.11,14–22 Overall, while effective bactericidal responses have been achieved on some nanostructured topographies, many reports still show limited efficacy. This supports the widely recognized challenge of eliminating Gram-positive bacteria, which are less susceptible to nanotopographic effects due to their thicker and mechanically stiffer cell envelope.

In summary, although direct comparison between our results and those from previous studies is constrained by differences in experimental conditions and incubation times, the findings presented here demonstrate bactericidal efficiencies that are consistent with current literature and underscore the inherent difficulty of designing effective nanotopographies against Gram-positive bacteria.

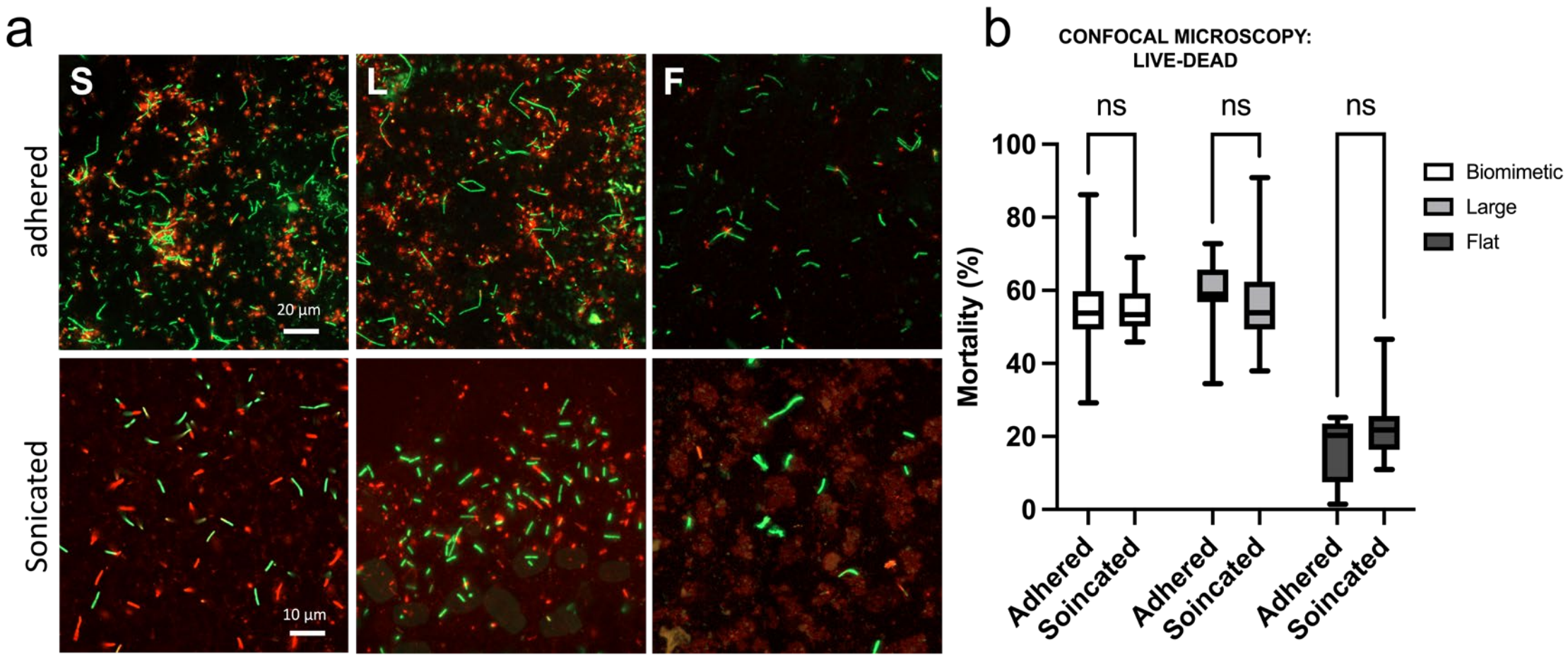

Figure S4. Assessment of the impact of the sonication protocol (30 s in 40 µL PBS) on bacterial viability and mortality. *To assess whether sonication affected bacteria mortality, we compared Live/Dead staining of bacteria adhered to the topographies with those detached following the sonication protocol. Live/Dead staining of detached bacteria was performed as follows: each PBS suspension of sonicated bacteria was placed on a confocal cover lid, and 0.5 µL of Live/Dead reagent was added directly to each suspension. Images were acquired after 7 min incubation and up to a maximum of 15 min, in accordance with the manufacturer's recommended incubation window. (a) Confocal images of adhered (top row) and sonicated (bottom row) bacteria from Small (S), Large (L), and Flat (F) topographies. Notably, the red background of sonicated bacteria corresponds to CDHA crystals. (b) Boxplots of mortality of sonicated and adhered bacteria after 3 h (n=12). Box plots indicate median (middle line), 25th, 75th percentile (box) and 5th and 95th percentile (whiskers). No outliers were observed. Statistical differences were assessed with 1-sample t-test (two-sided, P-value < 0.05). Confocal images are available on Zenodo public repository.*[25]

Table S3. Specific Surface area, porosity (pore volume) and pore size of the substrates measured by nitrogen adsorption.

	Specific surface area (m^2/g)	Pore volume (cm^3/g)	Pore size (nm)
F	41.548	0.095	8.5
S	1.493	0.003	10.7
L	6.636	0.042	22.1

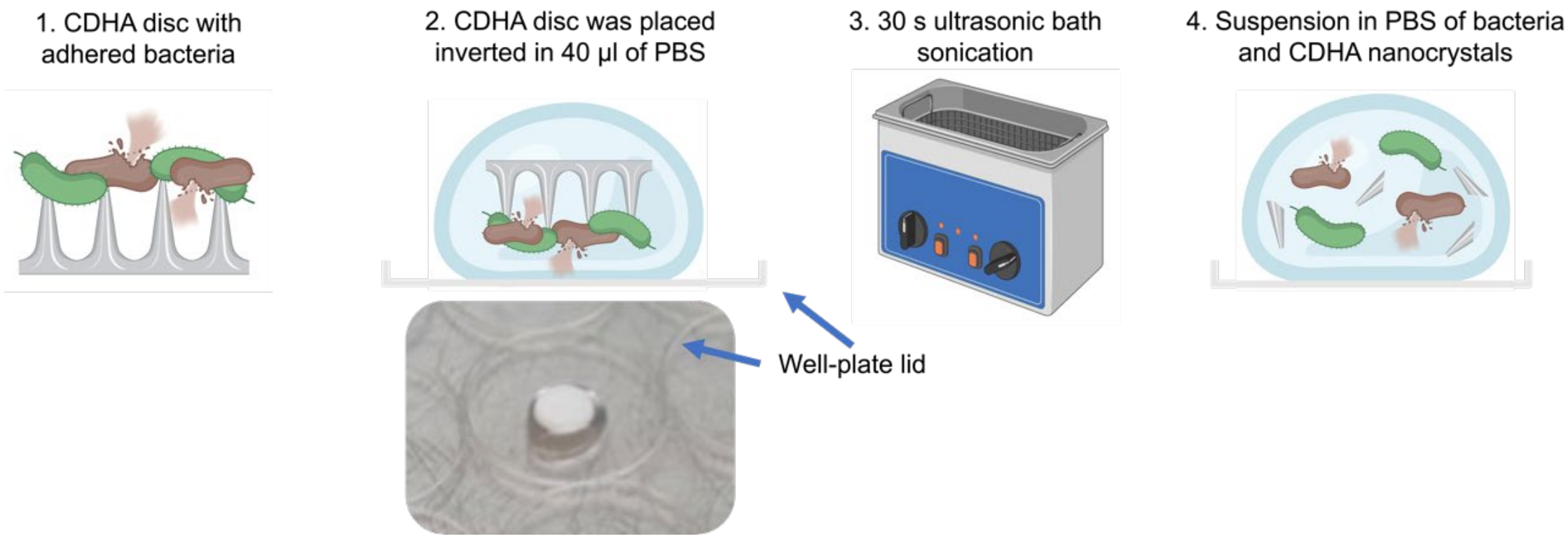

Figure S5. Schematic representation of the bacterial detachment protocol. *(1) CDHA substrates were washed three times with PBS to remove non-adherent bacteria. (2) Each disc was placed face-down onto a 40 µL drop of PBS positioned on the inner surface of a 24-well plate lid. (3) The lid was then floated on the water surface of an ultrasonic bath (Sonorex Digitec DT 52, Bandelin; nominal power 60 W) and sonicated for 30 s, releasing adherent bacteria together with CDHA nanocrystals into suspension (4). Created in BioRender. Ginebra, M. P. (2026) https://BioRender.com/eratgkl and released under a Creative Commons Attribution-NonCommercial-NoDerivs 4.0 International license.*

Supplementary Python 3 Script for processing XANES spectra.

The script processed data as follows: first, the contribution of lower-energy absorption was eliminated by subtracting a baseline. This baseline was calculated with a polynomial fit at the energies of the pre-edge, post-edge, and the absorbance at the local minimum between L_2 and L_3 edges (350-351 eV). Then, the signal was smoothed with the Savitzki-Golay filter, fitted with a 2nd-degree polynomial order.

The script can be copied and paste to code editor after the following line:

```
#!/usr/bin/env python3
# -*- coding: utf-8 -*-
"""
Created on Mon Jun 17 16:28:01 2024

@author: Marc Iglesias Fernandez
marc.iglesias.fernandez@gmail.com

If this code is used for your research, please cite this article.
"""


'''
INPUT DATA FORMAT: This code is designed to process .txt files conaining your
spectras data in columns in the following format:
    - 1st column contain the X axis values, that is the energy (eV) of each
    absorbance value. First row of the column must be the title,"eV" in
    this case
    - Other columns: contain the corresponding absorbance values of your
    different samples. First row is the name of the object analyzed. Use
    underscore as blank spaces for the titles names.

For example:

eV     title_sample_1 title_sample_2 [...]
x1     y1_sample1     y1_sample2
x2     y2_sample1     y2_sample2
x3     y3_sample1     y3_sample2
x4     y4_sample1     y4_sample2
[...]


The code is split in two parts:
    1st contains the functions created. Do not modify this part
    2nd part: scripts that can be modified to adjuts the plots.
'''


'''
FIRST PART OF THE CODE: DEFINITION OF FUNCTIONS TO OPERATE. DO NOT MODIFY THIS
PART OF THE CODE
'''
import matplotlib.pyplot as plt
import numpy as np

"""------------------------------raw data processing------------------------"""

'''FUNCTION TO IMPORT COLUMNS FROM THE .txt FILE'''
def arxiu_a_columnes(filename):
    with open(filename, 'r') as file:
        # Extract the first row (column titles)
        titles = file.readline().strip().split()
```

```
        # Load the numerical data from the file, skipping empty lines
        lines = (line.strip() for line in file if line.strip())
        llista_columnes = np.loadtxt(lines, skiprows=1)

        x = llista_columnes[:, 0]
        ys = llista_columnes[:, 1:].T

    return ys, x, titles

'''FUNCTION TO SELECT THE RANGE OF X VALUES TO PLOT'''
def retalla_func(x, ys,  x_ini, x_fin):
    index_peaks_Ca = np.where((x >= x_ini) & (x <= x_fin))
    x_tallat = x[index_peaks_Ca]
    ys_tallat = [col[index_peaks_Ca] for col in ys]
    return x_tallat, ys_tallat

'''FUNCTION TO SMOOTH SPECTRAS'''
from scipy.signal import savgol_filter
def smooth(ys, window_length, poly_order):
    # Apply Savitzky-Golay filter to each column
    smooth_y = [savgol_filter(col, window_length, poly_order) for col in ys]
    return smooth_y

'''baseline substraction defined by the preedge, postedge & minimum between edges'''
from numpy.polynomial import Polynomial

'''FUNCTION TO CALCULATE THE BASELINE TO SUBSTRACT YOUR DATA'''
'''
HOW IT WORKS: it calculates a polinomial function fitted using the desired
values in the preedge, values in the postedge after L2 and L3 Ca peaks, and the
local minimunn between L2 and L3 Ca peaks.
'''
def calculate_baseline(x, ys, degree, x1_minims, x2_minims,
                       punts_preedge, punts_postedge):
    corrected_ys = []
    baselines=[]
    for spectrum in ys:

        minima_indices=np.where((x>=x1_minims)&(x <= x2_minims))
        # Create x and y data for the polynomial fit
        x_minims=x[0:punts_preedge]
        y_minims=spectrum[0:punts_preedge]
        x_minims = np.append(x_minims, x[minima_indices])
        y_minims = np.append(y_minims, spectrum[minima_indices])
        x_minims= np.append(x_minims,x[(punts_postedge*-1):])
        y_minims = np.append(y_minims, spectrum[(punts_postedge*-1):])

        # Fit a polynomial to the selected minima points
        polynomial = Polynomial.fit(x_minims, y_minims, degree)

        # Calculate the baseline using the polynomial function
        baseline = polynomial(x)
        # Subtract the baseline from the spectrum
        corrected_spectrum = spectrum - baseline

        corrected_ys.append(corrected_spectrum)
        baselines.append(baseline)
    return np.array(corrected_ys), baselines


'''____________________bring the minimum to zero__________________________'''
'''in case any spectra after baseline sbstraction has values below 0,
translates the minimum value to 0. This function can be skipped when data
processing'''
def porta_a_0(x, ys):
    normalized_y = []
```

```
    #zona_interes = np.where((x >= x_ini) & (x <= x_fin))
    for col in ys:
        #normalized_col = np.interp(col, (col[zona_interes].min(), col[zona_interes].max()), (0,
col[zona_interes].max()-col[zona_interes].min()))
        normalized_col = np.interp(col, (col.min(), col.max()), (0, col.max()-col.min()) )
        normalized_y.append(normalized_col)
    return normalized_y




"""---------------------------------------------------------------------"""
"""-----------------------MAIN PROGRAM-----------------------------------"""
"""---------------------------------------------------------------------"""
'''
2nd part of the code: IN THIS SECTION, INDICATED PARTS, CAN BE MODIFIED
ACCORDING TO THE USERS NEEDS
'''


'''
TO MODIFY BY THE USER: write the file name containing the data to process
in the variable "filename" example: filename = 'Write_your_file_name.txt'.

Here are created variables for the files attached as example. Below are shown
the values to modify in their respective variables for getting the optimal
plots for the spectras.
'''

filename = 'Tomo31 Flat PAPER.txt'
### optimal plotting conditions: Baseline --> degree = 3
###                    window_length = 17
###                    punts_preedge = 5
###                    punts_preedge = 5


#filename = 'Tomo11 Boiled paper.txt' # Baseline --> degree = 3
### optimal plotting conditions: Baseline --> degree = 3
###                    window_length = 17
###                    punts_preedge = 5
###                    punts_preedge = 5

#filename = 'CDHA ref.txt'
### optimal plotting conditions: Baseline --> degree = 3
###                    window_length = 3 (smoothing no needed)
###                    punts_preedge = 18 (spectra step size is narrower => more preedge measured)
###                    punts_preedge = 10



'''(not necessary to modify) write the axis titles of the plots____________'''
plt.xlabel('photon energy (eV)')
plt.ylabel('absorption (a.u.)')


'''______________________ploting of the raw data____________________________'''
'''
IMPORTS .txt FILE of the filename var containing the data and converts it
in the following vars:
    titles-->list of the 1st row with sample titles
    x-->list of the first column (energy steps)
```

```
    ys_raw-->conatins the other columns (absorbance)
'''
ys_raw, x, titles = arxiu_a_columnes(filename)
#plots raw data in separate plots
for i in range(len(ys_raw)):
    plt.plot(range(len(x)),ys_raw[i],label=titles[i+1], linewidth=1)
    plt.title("raw data", fontsize=16, fontweight='bold', ha='center')
    plt.legend()
    plt.show()



'''__________________________baseline polyfit_________________________________'''
'''
CALCULATES BASELINE TO SUBSTRACT. Returns:
    ys_flat--> list with the list of raw spectra datas minus baseline
    baselines--> list with the baselines for each spectra raw data
TO MODIFY BY THE USER:

    degree= Insert the plynomial degree to fit data. for ex 3

    punts_preedge= Numer of dots (X axis) of the preedge to fit, starting the
                form 340 eV. For example, if this value is 5, the baseline
                is going to use values from 340 eV to 345 eV

    punts_postedge= As punts_preedge, but counting backwards from your last X
                energy avaiable. For example, if this value is 5,
                the baseline is going to use values from 360 eV to 355 eV

    x1_minims= Definition of the initial energy value (X axis) in which is
            found the minimum to fit between L2 and L3 peaks. For ex. 349.8

    x2_minims= Definition of the initial energy value (X axis) in which is
            found the minimum to fit between L2 and L3 peaks. For ex. 351.3
'''

ys_flat, baselines=calculate_baseline(x, ys_raw, degree=3, x1_minims=349.8,
                                    x2_minims=351.3, punts_preedge=5,
                                    punts_postedge=5)
#plots raw data with its baseline
for i in range(len(ys_raw)):
    plt.plot(x,ys_raw[i],label=titles[i+1])
    plt.plot(x,baselines[i])
    plt.title("Baseline for "+titles[i], fontsize=16, fontweight='bold', ha='center')
    plt.legend()
    plt.show()




'''-----------------------SMOOTH SPECTRA----------------------------------'''
'''
Returns: ys_smooth--> list containing a list of the smoothed signals of your
                spectras
TO MODIFY BY THE USER:
    window_length = the higher, the smoother.
    poly_order = degree of the interpolation between dots.
'''
ys_smooth=smooth(ys_flat, window_length = 17, poly_order = 2)
#plot of smoothed data with negative values
for i in range(len(ys_smooth)):
    plt.plot(x,ys_smooth[i],label=titles[i+1])
    plt.title("Smooth of "+titles[i], fontsize=16, fontweight='bold', ha='center')
    plt.legend(fontsize='small')
    plt.show()

'''---------------------PLOT THE X DESIRED RANGE---------------------------'''
```

```
'''plots y values between initial < x < final
Returns:
    x_tallat-->list of X values in the desired range
    ys_tallat-->List of y lists values in the desired range

(NOT NECESSARY) TO MODIFY BY THE USER:

    x_ini= initial x value to plot

    x_fin= final x value to plot

'''
x_tallat, ys_tallat=retalla_func(x, ys_smooth, x_ini=345, x_fin=355)
#plot selected x range
for i in range(len(ys_tallat)):
    plt.plot(x_tallat,ys_tallat[i],label=titles[i+1])
plt.title("x range for "+titles[i], fontsize=16, fontweight='bold', ha='center')
plt.legend(fontsize='small')
plt.show()



'''--------------------translate all spectras to minimum=0-----------------'''
'''
if baseline substraction leads to negative values, brings the minimum to 0
'''
ys_a0=porta_a_0(x_tallat, ys_tallat)
#plot smooth data
for i in range(len(ys_a0)):
    plt.plot(x_tallat,ys_a0[i],label=titles[i+1])
plt.title("a zero tot", fontsize=16, fontweight='bold', ha='center')
plt.legend(fontsize='small')
plt.show()



'''-------------------------------PLOT OF THE FINAL SPECTRAS---------------'''
num_columns = len(ys_a0)// 2
for espectre in range(len(ys_a0)):

    '''_____________________________PLOT_________________________________'''
    ####--------------------------nom eixos---------------------------
    plt.xlabel('eV')
    plt.ylabel('absorbance (a.u.)')
    ####------espectre
    plt.plot(x_tallat,ys_a0[espectre], label=titles[espectre+1]) #grafica espectre triat
    plt.title(titles[espectre+1], fontsize=16, fontweight='bold', ha='center')
    plt.legend(fontsize='small')
    plt.show()


"""
TO PLOT DATA IN OTHER SOFTWARES, Y VALUES ARE STORED IN ys_a0 and X VALUES ARE
STORED IN x_tallat
"""
```

Supplementary references

1. Iglesias-Fernandez, M., Pérez-Berná, A. J., Pereiro, E., Ginebra-Molins, M.-P. & Espanol, M. Figure 7 & S3: Synchrotron cryo-soft X-ray microscopy sheds light on the interaction of bacteria with nanostructured calcium phosphate substrates. (2026) doi:https://doi.org/10.5281/zenodo.18598093.
2. Ivanova, E. P. et al. Bactericidal activity of black silicon. Nat. Commun. 4, 1–7 (2013).
3. Mainwaring, D. E. et al. The nature of inherent bactericidal activity: Insights from the nanotopology of three species of dragonfly. Nanoscale 8, 6527–6534 (2016).
4. Green, D. W. et al. High Quality Bioreplication of Intricate Nanostructures from a Fragile Gecko Skin Surface with Bactericidal Properties. Sci. Rep. 7, 1–12 (2017).
5. Wu, S., Zuber, F., Maniura-Weber, K., Brugger, J. & Ren, Q. Nanostructured surface topographies have an effect on bactericidal activity. J. Nanobiotechnology 16, 1–9 (2018).
6. Hasan, J., Raj, S., Yadav, L. & Chatterjee, K. Engineering a nanostructured 'super surface' with superhydrophobic and superkilling properties. RSC Adv. 5, 44953–44959 (2015).
7. Buhl, S., Stich, A., Bulitta, C., Zapata, C. & Paniagua, S. Proof of the effectiveness of novel bioinspired antimicrobial nanostructures. Curr. Dir. Biomed. Eng. 10, 131–134 (2024).
8. Wu, S., Zuber, F., Brugger, J., Maniura-Weber, K. & Ren, Q. Antibacterial Au nanostructured surfaces. Nanoscale 8, 2620–2625 (2016).
9. Viela, F. et al. Nano-engineering safer-by-design nanoparticle based moth-eye mimetic bactericidal and cytocompatible polymer surfaces. RSC Adv. 8, 22606–22616 (2018).
10. Chen, C. et al. Bactericidal surfaces prepared by femtosecond laser patterning and layer-by-layer polyelectrolyte coating. J. Colloid Interface Sci. 575, 286–297 (2020).
11. Susarrey-Arce, A. et al. Bacterial viability on chemically modified silicon nanowire arrays. J. Mater. Chem. B 4, 3104–3112 (2016).
12. Izquierdo-Barba, I. et al. Nanocolumnar coatings with selective behavior towards osteoblast and Staphylococcus aureus proliferation. Acta Biomater. 15, 20–28 (2015).
13. Zhao, L. et al. Low-Temperature Hydrothermal Synthesis of Novel 3D Hybrid Nanostructures on Titanium Surface with Mechano-bactericidal Performance. ACS Biomater. Sci. Eng. 7, 2268–2278 (2021).
14. Sengstock, C. et al. Structure-related antibacterial activity of a titanium nanostructured surface fabricated by glancing angle sputter deposition. Nanotechnology 25, (2014).
15. Jenkins, J. et al. Antibacterial effects of nanopillar surfaces are mediated by cell impedance, penetration and induction of oxidative stress. Nat. Commun. 11, (2020).
16. Cao, Y. et al. Nanostructured titanium surfaces exhibit recalcitrance towards Staphylococcus epidermidis biofilm formation. Sci. Rep. 8, 1–13 (2018).
17. Jaggessar, A. et al. Mechanical, bactericidal and osteogenic behaviours of hydrothermally synthesised TiO 2 nanowire arrays. J. Mech. Behav. Biomed. Mater. 80, 311–319 (2018).
18. Bhadra, C. M. et al. Antibacterial titanium nano-patterned arrays inspired by dragonfly wings. Sci. Rep. 5, 1–12 (2015).
19. Truong, V. K. et al. The susceptibility of Staphylococcus aureus CIP 65.8 and Pseudomonas aeruginosa ATCC 9721 cells to the bactericidal action of nanostructured Calopteryx haemorrhoidalis damselfly wing surfaces. Appl. Microbiol. Biotechnol. 101, 4683–4690 (2017).
20. Fontelo, R., Soares da Costa, D., Reis, R. L., Novoa-Carballal, R. & Pashkuleva, I. Bactericidal nanopatterns generated by block copolymer self-assembly. Acta Biomater. 112, 174–181 (2020).
21. Diu, T. et al. Cicada-inspired cell-instructive nanopatterned arrays. Sci. Rep. 4, (2014).
22. Hizal, F. et al. Impact of 3D hierarchical nanostructures on the antibacterial efficacy of a bacteria-triggered self-defensive antibiotic coating. ACS Appl. Mater. Interfaces 7, 20304–20313 (2015).
23. Linklater, D. P., Nguyen, H. K. D., Bhadra, C. M., Juodkazis, S. & Ivanova, and E. P. Influence of nanoscale topology on bactericidal efficiency of black silicon surfaces. Nanotechnology 28, 9 (2017).
24. Iglesias-Fernandez, M., Buxadera-Palomero, J., Sadowska, J.-M., Espanol, M. & Ginebra, M.-P. Implementation of bactericidal topographies on biomimetic calcium phosphates and the potential effect of its reactivity. Biomater. Adv. 212797 (2022).
25. Iglesias-Fernandez, M., Pérez-Berná, A. J., Pereiro, E., Ginebra-Molins, M.-P. & Espanol, M. Figure S4: Synchrotron cryo-soft X-ray microscopy sheds light on the interaction of bacteria with nanostructured calcium phosphate substrates. (2026) doi:https://doi.org/10.5281/zenodo.19135718.
