# Supplementary material for: Synchrotron cryo-soft X-ray microscopy reveals bacterial interactions with nanostructured calcium phosphate substrates
Source: Nat Commun. 2026 Jul 13;17:8626. doi: 10.1038/s41467-026-75323-y (PMC13487168; doi:10.1038/s41467-026-75323-y)
Supplement: Supplementary file 2 [file 41467_2026_75323_MOESM2_ESM.pdf]

The Description of Additional Supplementary Files

Supplementary Movie 1: Visualisation of a 3D segmentation of bacteria incubated on small pillars displaying multivesicular bodies (Fig. 5 b1). The tomography was acquired at 520eV. The video shows the membrane (yellow), vesicles (red), and multivescicular bodies (blue). The video was created with Avizo AMIRA software.

Supplementary Movie 2: Visualization of a 3D segmentation of bacteria incubated on small pillars displaying multivesicular bodies (Fig. 5 b2). The tomography was acquired at 520eV. The video shows the membrane (yellow), cytoplasmic membrane (green), vesicles (red), and multivesicular bodies (blue). The video was created with Avizo AMIRA software.

Supplementray Movie 3: Video of the stack of 2D images acquired throughout the L3,2 calcium edge (340-360 eV), displaying bacteria incubated on the Flat substrate. Each frame corresponds to a different energy. Note that the closer the frame was acquired near the L3,2 edges, the greater is the contrast of the Cacontaining areas.

Supplementary Movie 4: Video of the cryo Soft X-ray tomography acquisition at the L2- calcium edge displaying bacteria incubated on small pillars. Projections are acquired in a range of 101°. Each frame corresponds to an angle.

Supplementary Movie 5: 3D reconstruction of a tomography acquired at the L2- calcium edge displaying bacteria incubated on small pillars. Each frame corresponds to a slice of the 3D reconstruction.
