# Supplementary material for: Synchrotron cryo-soft X-ray microscopy reveals bacterial interactions with nanostructured calcium phosphate substrates
Source: Nat Commun. 2026 Jul 13;17:8626. doi: 10.1038/s41467-026-75323-y (PMC13487168; doi:10.1038/s41467-026-75323-y)
Supplement: Supplementary file 3 [file 41467_2026_75323_MOESM8_ESM.pdf]

nature portfolio

Corresponding author(s): Maria-Pau Ginebra Molins

Last updated by author(s): 19-5-2026

Reporting Summary

Nature Portfolio wishes to improve the reproducibility of the work that we publish. This form provides structure for consistency and transparency in reporting. For further information on Nature Portfolio policies, see our Editorial Policies and the Editorial Policy Checklist.

Statistics

For all statistical analyses, confirm that the following items are present in the figure legend, table legend, main text, or Methods section.

n/a	Confirmed	
☐	☒	The exact sample size (*n*) for each experimental group/condition, given as a discrete number and unit of measurement
☐	☒	A statement on whether measurements were taken from distinct samples or whether the same sample was measured repeatedly
☐	☒	The statistical test(s) used AND whether they are one- or two-sided<br>*Only common tests should be described solely by name; describe more complex techniques in the Methods section.*
☐	☒	A description of all covariates tested
☐	☒	A description of any assumptions or corrections, such as tests of normality and adjustment for multiple comparisons
☐	☒	A full description of the statistical parameters including central tendency (e.g. means) or other basic estimates (e.g. regression coefficient) AND variation (e.g. standard deviation) or associated estimates of uncertainty (e.g. confidence intervals)
☐	☒	For null hypothesis testing, the test statistic (e.g. *F*, *t*, *r*) with confidence intervals, effect sizes, degrees of freedom and *P* value noted<br>*Give P values as exact values whenever suitable.*
☒	☐	For Bayesian analysis, information on the choice of priors and Markov chain Monte Carlo settings
☒	☐	For hierarchical and complex designs, identification of the appropriate level for tests and full reporting of outcomes
☒	☐	Estimates of effect sizes (e.g. Cohen's *d*, Pearson's *r*), indicating how they were calculated

Our web collection on statistics for biologists contains articles on many of the points above.

Software and code

Policy information about availability of computer code

Data collection	Topography SEM images with SMARTSEM Version 6.00 with service pack 5 and Tescan Essence. XRD spectra with D8tools-02B Version 5.02. Confocal microscope with Zeiss Zen 2.6 Blue edition.
Data analysis	Statistics with Prism 10.2.3 (347), Minitab 19.2020.1, Image analysis with Fiji ImageJ 1.54f, XRD and crytalinity with Profex 5.2.9, XANES with custom script, cryo-SXT segmentation and LAC calculation with AMIRA 2021.1.

For manuscripts utilizing custom algorithms or software that are central to the research but not yet described in published literature, software must be made available to editors and reviewers. We strongly encourage code deposition in a community repository (e.g. GitHub). See the Nature Portfolio guidelines for submitting code & software for further information.

Data

Policy information about availability of data

All manuscripts must include a data availability statement. This statement should provide the following information, where applicable:

- Accession codes, unique identifiers, or web links for publicly available datasets
- A description of any restrictions on data availability
- For clinical datasets or third party data, please ensure that the statement adheres to our policy

Source Data are provided with this paper. The source data underlying Figures 1b-g, Table 1, 2b-d, 3, 4b, 6b, 7, 8c-d, and Supplementary Figures S3 and S4 are provided as a Source Data file. Cryo-SXT raw data shown in Fig. 5, 6a, 7a, 8a-b, and Supplementary Figure S3 were generated at ALBA synchrotron BL09 Mistral

Beamline. The normalized tilt series, reconstructed tomographies, XANES image stacks and calcium maps data have been deposited in the Electron Microscopy Public Image Archive (EMPIAR) under accession codes EMPIAR-13280 [https://www.ebi.ac.uk/empiar/EMPIAR-13280] (.hdf5 files are normalized tilt series; .mrc or ended in "3DS30" are reconstructed tomographies; archives including "lnWOFF" or similar text are for LAC quantification; Archives including "XANES" text are for spectra acquisition.); Confocal microscopy datasets, are deposited in the ZENODO repository at the following links: Fig. 2 (https://doi.org/10.5281/zenodo.19135539),
Fig. 4 (https://doi.org/10.5281/zenodo.19135331),
Fig. S4 (https://doi.org/10.5281/zenodo.19135718).

Research involving human participants, their data, or biological material

Policy information about studies with human participants or human data. See also policy information about sex, gender (identity/presentation), and sexual orientation and race, ethnicity and racism.

Reporting on sex and gender	Human sex and gender has not been collected as it is not relevant for microbiological experiments
Reporting on race, ethnicity, or other socially relevant groupings	This information has not been collected as it is not relevant for microbiological experiments
Population characteristics	This information has not been collected as it is not relevant for microbiological experiments
Recruitment	This information has not been collected as it is not relevant for microbiological experiments
Ethics oversight	This information has not been collected as it is not relevant for microbiological experiments

Note that full information on the approval of the study protocol must also be provided in the manuscript.

Field-specific reporting

Please select the one below that is the best fit for your research. If you are not sure, read the appropriate sections before making your selection.

☒ Life sciences ☐ Behavioural & social sciences ☐ Ecological, evolutionary & environmental sciences

For a reference copy of the document with all sections, see nature.com/documents/nr-reporting-summary-flat.pdf

Life sciences study design

All studies must disclose on these points even when the disclosure is negative.

Sample size	No statistical sample-size calculation was made. Sample size was n=9 for experiment, sum of three biological replicates with three technical replicates. Confocal imaging results sample size was n=12, as four images were acquired per condition on each experiment.
Data exclusions	For LAC quantification, the values of dead bacteria were excluded on the analysis due to the reasons justified in the article
Replication	Reproducibility was assessed performing all experiments three times on different days. Three technical replicates were assessed per experiment and four images were acquired per condition on confocal microscopy experiments. Cryo-SXT experiments were performed throughout 4 different beamtimes.
Randomization	Allocation was not random as it is not relevant for the experimental performed in this study.
Blinding	Blinding is not relevant in this study due to the narute of the experimental.

Reporting for specific materials, systems and methods

We require information from authors about some types of materials, experimental systems and methods used in many studies. Here, indicate whether each material, system or method listed is relevant to your study. If you are not sure if a list item applies to your research, read the appropriate section before selecting a response.

Materials & experimental systems

n/a	Involved in the study
☒	☐ Antibodies
☒	☐ Eukaryotic cell lines
☒	☐ Palaeontology and archaeology
☒	☐ Animals and other organisms
☒	☐ Clinical data
☒	☐ Dual use research of concern
☒	☐ Plants

Methods

n/a	Involved in the study
☒	☐ ChIP-seq
☒	☐ Flow cytometry
☒	☐ MRI-based neuroimaging

Plants

Seed stocks	no seed stock were used in this study
Novel plant genotypes	no novel plant genotypes were used in this study
Authentication	no seed stock and novel plant genotypes were used in this study
