# Supplementary material for: Synchrotron cryo-soft X-ray microscopy reveals bacterial interactions with nanostructured calcium phosphate substrates
Source: Nat Commun. 2026 Jul 13;17:8626. doi: 10.1038/s41467-026-75323-y (PMC13487168; doi:10.1038/s41467-026-75323-y)
Supplement: Supplementary file 4 [file 41467_2026_75323_MOESM9_ESM.pdf]

nature portfolio

Peer Review File

Synchrotron cryo-soft X-ray microscopy reveals bacterial interactions with nanostructured calcium phosphate substrates

Corresponding Author: Professor Maria-Pau Ginebra

This file contains all reviewer reports in order by version, followed by all author rebuttals in order by version.

Version 0:

Reviewer comments:

Reviewer #1

(Remarks to the Author)
This study aims to understand the potential mechano-bactericidal and chemical effects of nanopillared calcium-deficient hydroxyapatite (CDHA) on Gram-positive Bacillus subtilis. The same group previously reported promising mechano-bactericidal activity of CDHA against Gram-negative Pseudomonas aeruginosa. The current study complements that work by examining the phenomenon with Gram-positive B. subtilis. It is well established in the mechano-bactericidal literature that Gram-positive bacteria have thicker cell envelopes and are therefore more resistant to nanotopography-mediated mechanical perturbation. In addition to using a Gram-positive rather than a Gram-negative species, another enhancement is the use of cryo-SXM to probe physical and chemical changes within cells in a near-native aqueous environment during interaction with various CDHA samples and controls. The authors reported up to 50% reduction achieved (CDHA with large pillars) in B. subtilis after 3-h static incubation in PBS.
***Strength:
This study presents solid, comprehensive data that enrich the bacteria-material interaction literature. The experiments are clearly well thought out, and the effort to present the whole picture is commendable. Thorough characterization is provided for the materials, the liquid environment, and the bacterial cells, and the main conclusions are supported by the data. A notable strength is the use of complementary techniques—fluorescence microscopy, ICP-MS, and XANES—to trace Ca^{2+} flow from CDHA to the surroundings and ultimately into the cytosol. Consistency between the fluorescence and XANES data strengthens their reliability. Cryo-SXM is still a relatively new method for studying bacteria–material interactions under close-to-native conditions – this study provides another solid case study of implementing these novel tools in understanding bacteria-material interactions.
***Weakness:
Although the study provides several new observations through cryo-SXM, it is unclear how these findings might help improve mechano-bactericidal efficacy against Gram-positive bacteria.
-The authors reported up to 50% reduction achieved (CDHA with large pillars) in B. subtilis after 3-h static incubation in PBS. How does that compare with results achieved in prior studies using BS or other Gram+ bacteria?
-I have concerns about “selection bias” in surveying and quantifying the intracellular Ca2+ concentration, which may be intrinsic to XANES technique itself. XANES only looked at the cells that are still intact and are dislodged from the material surfaces, following 40s sonication. The conclusions drawn on intracellular Ca2+ concentration are applicable only to these selected cells, but it is not discussed how representative that selected population was of the whole population on material surfaces in their native state. Take, for example, the ruptured cells (close 50% for L) – what were their Ca2+ level? Could they have had ruptured (but not their peers) because they had higher/lower intracellular Ca2+? They were completely excluded in the XANES analysis… Fluorescent data may help address this. A more detailed discussion comparing/contrasting the fluorescent and XANES results may help too.
-The authors convincingly show that cryo-SXT and XANES can resolve subcellular structures such as vesicle formation and Ca^{2+} accumulation; however, the biological significance of these observations, particularly in relation to mechano-bactericidal efficacy, need to be discussed in greater details.
-Can the authors formulate new hypotheses on how nanotopography and surface chemistry could be modified to enhance the bactericidal efficacy of CDHA—or similar materials—based on these observations? This is a major weakness of the study.
-While cryo-SXT and XANES are still fairly new in the field, these techniques have already been used to explore whole-cell

ultrastructure (e.g., doi: 10.1042/ETLS20170086). This raises the bar for novelty, and demonstrating biological significance or proposing material-engineering insights derived from these techniques is essential to appeal to the readership of Nature Communications.

Reviewer #2

(Remarks to the Author)
General Assessment:
The work presented here is original and addresses a timely topic—the need for antibiotic-free antibacterial surfaces to mitigate antibiotic resistance. The authors combine advanced materials synthesis (CDHA nanopillars) with state-of-the-art imaging techniques (Cryo-SXT and XANES) to investigate bacteria–surface interactions. However, significant methodological flaws raise concerns about the validity of the conclusions. Below, I provide a structured review highlighting both strengths and critical weaknesses that must be addressed to make this work suitable for publication.

Novelty and Impact

The study is original and interdisciplinary, bringing together materials science, microbiology, and advanced imaging.
The application of Cryo-SXT and XANES is innovative for analyzing bacterial interactions with surfaces and Ca incorporation.
However, critical methodological flaws in sample preparation and data analysis compromise the validity of key conclusions. These issues are detailed in subsequent sections.

Does the Work Support the Conclusions and Claims?

Currently, the data do not robustly support the main claims, particularly regarding the bactericidal effect of the surfaces and the role of calcium uptake. Specific concerns:

The Live/Dead staining results are invalid due to fixation prior to staining.

SEM sample preparation leads to artifacts (e.g., cell collapse), rendering morphological interpretations unreliable.

Sonication-based sample preparation likely detaches or damages both bacteria and nanostructures, undermining claims about cell wall damage localization.

No direct evidence of bacterial viability (e.g. CFU counts) is presented.

Comment on bone integration of the material are made without supporting data.

3. Major Flaws / Drawbacks in the Experimental Setup

3.1 Live/Dead Staining

Critical Flaw: The cells are fixed before Live/Dead staining. This renders the assay invalid, as fixed cells are no longer viable and cannot accurately differentiate between live and dead populations. Please repeat these experiments using a fixative-compatible Live/Dead protocol or alternative method that preserves viability.

3.2 SEM Sample Preparation

After dehydration, samples are coated with carbon. This procedure induces shrinkage and collapse artifacts, distorting the bacterial morphology. For accurate bacterial visualization, critical point drying (CPD) or HMDS drying must be used. Please repeat these experiments using an appropriate protocol.

3.3 Sonication Procedure

The manuscript lacks detail about the sonication step:

What type of sonication was performed,and what energy was used? Sonication requires the sample to be immersed in liquid, yet 40 µL is insufficient to fully immerse the samples. Please clarify this procedure in the Methods section.

Additionally, 30-second sonication in ethanol reportedly caused nanopillar detachment from base—this suggests the structures/fabricated material are unstable for their intended application as implant coatings. How would these structures withstand clinical handling or in vivo conditions? Please address this concern regarding translational potential.

3.4 Sample Preparation for Cryo-SXT

The authors aim to study bacterial cell wall damage relative to the position of nanopillars. However, using sonication to

detach bacteria removes them from the surface, so their spatial relationship to the nanopillars is lost. This critically undermines the study's objective.

Moreover, if bacteria are strongly attached (as stated), detaching them with sonication could damage the cells, leading to artifacts in the Cryo-SXT images.

Notably, the TEM sample preparation shows that sonication for 30 seconds caused detachment of the nanopillars themselves. Why are nanopillars not observed in association with bacterial cells in Cryo-SXT?

Since cells are removed from the actual surface, this condition is no longer "near-native." Please update the manuscript to reflect this limitation and discuss its impact.

3.5 Cryo-SXT Observations

Figure 5a (L vi) shows cells without a visible cell wall, yet they maintain their shape. How is this possible? Please clarify.

4. Additional Data Analysis Concerns

4.1 Data Interpretation

The authors claim that only small nanopillars cause vesicle formation, but do not provide robust quantitative evidence to support this claim. What evidence you can provide for other cells, that similar vesicles are not present? Even in Figure 5a-i ii, iii darker cells do not show vesicles. Can this be a limitation of the technique?

Page 25, Lines 422–424: The claim that CDHA is porous after pressing, but not other substrates which are compressed.Please provide evidence, e.g. cross-section SEM images (currently unclear).

Page 25, Lines 426–428: P in F is low; S, L, and Ctrl have slightly higher P (Line 328). Is this due to PBS treatment?

Figure 2 shows high Ca2+ for F and L, but no corresponding increase in bactericidal action is reported. This suggests calcium has no effect on bactericidal action—please address this contradiction.

Calibration with known Ca2+ concentrations is not described. Are the confocal measurements relative or absolute?

4.2 XANES Spectroscopy

Figure 6: Please provide a pre-edge image to establish background contrast. The scale and field of view in the L2 edge and Ca map differ—please explain. Also, dark particles in the Ca map appear larger; why? Remove disruptive labels within image and use consistent symbols.

Figure 7:

Line 281: How many bacterial cells were analyzed via XANES? Please provide representative data for multiple cells.

The control images at 352.4 eV and 346.0 eV look identical. If there is no Ca, why is there absorption at the L2 edge in the control sample? Control samples should show lower absorbance than samples with Ca. Please explain this inconsistency in Lines 313-315.

How many cells were analyzed for each treatment group?

Use consistent y-axis scales across Figures 6 and 7.

Figure 8a–b: Scales and field of view differ. Please explain.

Line 347: Ca concentrations are reported for bacteria detached from the surface. How can this be considered " Ca concentrations of bacteria in contact with the nanotopography"?

4.3 Terminology and Consistency

There is inconsistency in referring to "CaP" and "CDHA" in text and in images. Are these terms interchangeable or distinct in your study? Please clarify throughout the manuscript.

5. Data Interpretation and Conclusions

The authors claim the substrate is suitable for bone integration. However, no experiments on osteointegration or relevant cell lines are presented. Please provide supporting data or temper the claims accordingly.

The effects of Ca and P are not clearly contributed for bactericidal action as per results. However, authors claim synergistic effects of surface chemistry and nanotopography on bactericidal action. This statement is not justified with the data from the manuscript.

6. Methodology

Please refer to Section 3 above for details. In its current form, the fluorescence microscopy and SEM sample preparation do not meet field standards and must be repeated using appropriate protocols.

7. Reproducibility

The manuscript generally provides sufficient detail, except for the sonication procedure, which lacks clarity on immersion and power settings. Please expand this section and consider adding a schematic to aid reproducibility. Ideally provide a schematic of the sample preparation in a single figure, so that workflow is clear.

Recommendation

Major Revision

Before publication, the authors must:
Repeat key experiments (Live/Dead staining, SEM imaging) using correct sample preparation methodologies.
Provide quantitative viability data (CFU or alternative) to support bactericidal claims.
Address concerns about sample preparation and discuss limitations of detached cells in Cryo-SXT.
Clarify inconsistencies in XANES data interpretation.
Provide evidence or a more cautious interpretation of bone integration claims.

I suggest the authors can address these points with appropriate data and discussion, the manuscript would significantly improve in rigor and reliability, potentially making it suitable for publication.

Reviewer #3

(Remarks to the Author)
This manuscript presents a study of the interaction between Bacillus subtilis and nanostructured calcium phosphate (CDHA) substrates, using synchrotron cryo-soft X-ray microscopy and XANES spectroscopy to explore both mechanical and chemical effects at the subcellular level. Ca L-edge XANES spectromicroscopy is used to resolve intracellular calcium accumulation in B. subtilis exposed to nanostructured CDHA surfaces. The authors demonstrate that calcium uptake is substrate-dependent, with higher internalisation observed on flat and large-pillar CDHA surfaces. The Ca maps extracted from the XANES spectra distinguish between calcium-rich and calcium-poor bacteria populations, and the XANES spectra suggest the presence of amorphous or soluble calcium species rather than crystalline deposits. Whilst the work has relevance in biomaterials and microbiology the article focusses on the application on the cryo-SXT technique and the information that this, in combination with Ca XANES, can provide in terms of visualising the morphology of the bacteria cell and locating and quantifying the Ca uptake. In this aspect some lovely data is presented, the reconstructions and segmentations are well considered and the methodology is well described. The significance and impact of the results in themselves is however less clear, beyond how the cryo-SXT method can be used, for example how do these results inform the design of nanostructured antibacterial surfaces.
Minor revisions and discussion points are suggested.
Page2 line 12 “Nanotropographies” should be “Nanotopographies”
Page 4 line 23: Cryo-SXR should be Cryo-SXT
Page 5 paragraph 1, fig 1: A representative plot of the Reitveld fit, and difference plot, should be presented, along with the refinement R-factor or goodness of fit. The CaP phase have multiple overlapping peaks therefore the quality of the fitting is important when considering the phase fractions. Errors should eb given on the phase fractions presented in Table 1. The authors state that the sharper peaks indicate increased crystallinity – have the peak widths been calculated? The peak widths are also affected by crystallite size, so it would be expected to have broader peak for the S morphologies, how then is extent of crystallinity determined?
Page 13: sample preparation for cryo_SXT – all bacteria were detached from substrates after 3 h incubation, could this detachment (and any bacteria damage)have occurred during transfer onto the substrate for vitrification eg. duringthe sonication?
How was the 3h incubation time chosen?
Ca – XANES:
How was the effect of beam damage considered and measured for the Ca XANES data collection, method section states no beam damage was observed?
For the identification of the spectra, plots of standard Hap Ca XANES and amorphous CaP should be provided as reference data for comparison. Ca spectra in a bacterial environment may be different to standards, are there any literature

comparisons for this?
In order to identify the location of different spectra in the maps, were any PCA and cluster analysis performed, or were the spectra isolated manually only by comparison to the cryo-SXT locations?
The bacterial Ca is described as being in the cytosol, how is surface absorption to the outside of the bacteria ruled out?

Version 1:

Reviewer comments:

Reviewer #1

(Remarks to the Author)
The authors have addressed all of my comments. No further comments/questions from me. I recommend publishing the revised manuscript.

Reviewer #2

(Remarks to the Author)
I thank the authors for the revised version of the manuscript. The authors have carefully considered my previous comments and have repeated experiments as suggested. Overall, most of the issues I raised have been addressed. Some points have been incorporated into the manuscript, and others have been reasonably explained.

However, I would like to emphasize that explanations given only in the response-to-reviewers should also be reflected in the manuscript text itself, so that future readers will not raise the same questions again. This is particularly important for the issues discussed under Comment 3.4.
I believe that after incorporating these clarifications into the manuscript, the work will be clearer, more informative, and at a quality suitable for Nature Communications.
I list few remaining points and clarifications below:
1. Metadata availability
As the data are not publicly available, I request that the authors provide the relevant metadata for the review process (e.g., acquisition parameters, processing steps, and any key intermediate files, as appropriate for the new data acquired on methodology).
2. Clarification of “cell envelope damage” (line 452 and response to Comment 3.4)
In line 452, the authors refer to “cell envelope damage,” and in their response to Comment 3.4 they state that the “goal was to assess the extent and nature of cellular damage”. However, cell envelope damage is not clearly demonstrated in any of the provided images, or the proximity of nanopillar to a damaged wall. The method, as presented, shows differences in cytoplasm intensity, from which cell wall damage is inferred, rather than directly resolved. This is conceptually similar to the interpretation of Live/Dead staining, where membrane damage is inferred from staining colour, but the damage itself is not directly visualized.
I recommend that the authors:
o Rephrase this part of the manuscript to make it explicit that cell envelope damage is inferred from intensity differences, rather than directly imaged; and/or
o Provide clearer supporting evidence, if available, that directly links the observed intensity changes to structural damage of the cell envelope.
3. Cryo-SXT: alignment of manuscript text with the response to Comment 3.4
The response to Comment 3.4 regarding the capabilities and limitations of Cryo-SXT is helpful and improves the understanding of the approach and its goals. At present, this explanation is largely confined to the response letter. I request that these clarifications be incorporated into the main text—specifically in the Discussion and, to a more concise extent, in the Introduction—to make the goal and interpretative framework of the Cryo-SXT measurements clearer for the reader.
4. Interpretation of vesicles vs. puncture/rupture on bactericidal mechanism (Figures 7a and 5)
As presented, Figures 7a and 5 do not clearly show puncture or rupture of the cell envelope by nanopillars. Instead, the main consistent observation appears to be the presence of vesicles, which is more suggestive of a general stress response rather than direct mechanical puncturing. I suggest that the authors:
o Explicitly acknowledge in the Discussion that no unambiguous puncture/rupture events are observed in these figures; and
o Clarify that the vesicle formation is interpreted as a stress response, and discuss how this fits within the broader mechanistic picture of bacterial interaction with nanopillars.
5. Visibility of nanopillars in Cryo-SXT and identification of nanopillars (Figure 5 and 7L,F)
In the response to the question about the absence of clearly visible nanopillars in one set of Cryo-SXT images, the authors state that “identifying nanopillars in Cryo-SXT images is not straightforward due to their nanoscale dimensions and the contrast limitations of the technique.” This is understandable; however, the structure indicated by the asterisk in the Figure 5 does not convincingly resemble a nanopillar. Is the dark spot next to cell in Figure5L a nanopillar? Figure 7L shows nanopillars, indeed.
I therefore ask the authors to:
o Reconsider and clarify the identification of the asterisk-marked structure in these panels;
o Provide a more cautious wording in the caption and main text, making it clear if the assignment as a nanopillar is tentative or inferred; and
Subject to these relatively minor clarifications and textual adjustments—most of which aim at aligning the claims with what is

directly supported by the data—I am satisfied with the scientific quality and clarity of the manuscript and consider it suitable for publication in Nature Communications.

Reviewer #3

(Remarks to the Author)
The manuscript has been thoroughly revised to address the reviwer comment, adding significant new data to support their conclusion. Importantly I feel that the authors make a convincing argument for novelty regarding the use of cryoSXT and XANES in bacterial cells, and the study is indeed a good showcase for the applicability of the technique in this area.
The concerns regarding selection bias in studying only intact cells is clearly addressed
Further work has been added to address the concerns of reviewer 2, large amount of extra work to repeat SEM studies and live/dead assays with different protocols, undertake CFU and metabolic assays has been carried out and added to the manuscript. Th information added does add tot eh size of the paper, I whilst I am not convinced it was all required the improved SEM methodology and details on the sonication in particular do add weight to the studies conclusions by addressing the sample selection and preparation concerns highlighted by all reviewers. The question regarding aiming to investigate the cell wall damage relative to nanopillar location is not a claim that the authors are making in the paper so sonication does not undermine their results – the question regrading introduction of artefacts is more valid and is to me suitably addressed by the additional assays and comparison of results across methodologies.
Regarding the XANES spectroscopy and figure 6, the changes in images/projection angles used do now make the presentation clearer. The apparent difference in particle size is consistent with the explanation provided of the refocusing required for each XANES energy acquisition, and does not detract from the results or interpretation provided in the manuscript.
The clarification regarding the repeat results on a number of cells is an important point, however it is recognised as a challenge more generally with these techniques, especially when performing XANES analysis due to the time taken for the data collection – and limitations on access to SR beamtime required – this I feel should not detract from the result observed and described in this work, as long as the authors are open about the extent, and consistency of the data collected as they have been here. The explanation of the choice of the 3h time point also highlights the challenge around access to experiment time, and is fully justified.
The interpretation of the XANES spectra is consistent with the use of the technique – a background in the control no Ca case would be expected as consistent with the explanation the authors provide, the Ca absorbance, and hence the Ca map, is obtained through the difference in the pre edge and edge images.
The addition of the Ca standard to the plots is helpful and adds clarity. The comments regarding the PCA analysis and selection of regions satisfactorily address the questions I had raised. Similarly, the question of 3D distribution using the LAC analysis is convincing
The authors have repeated the XRD analysis on different conditions and added the fit to the SI this is more convincing of the rigour of the analysis. The analysis of peak width also strengthens their comments around the crystallinity of the substrates.

Version 2:

Reviewer comments:

Reviewer #2

(Remarks to the Author)
I would like to thank the authors for their careful and constructive revision of the manuscript entitled “Synchrotron cryo-soft X-ray microscopy sheds light on the interaction of bacteria with nanostructured calcium phosphate substrates.” The revisions demonstrate a clear effort to address the concerns raised in the previous review rounds. Well done!

Overall, the updates are satisfactory. The manuscript has been improved through the incorporation of clarifications regarding the experimental design, methodological details, limitations of the methods used and interpretation of the results. The Discussion section now appropriately reflects the points raised during review, and the scientific narrative is clearer and coherent as a result.

I also appreciate the authors’ efforts toward increased data transparency, including the partial public deposition of SXT datasets. However, the live/dead confocal microscopy dataset—one of the key datasets requested in previous review—has not yet been made publicly available. Given its importance to the biological conclusions of the study, I strongly encourage the authors to deposit these data (at minimum the associated metadata and representative raw data) in an appropriate public repository to ensure reproducibility and transparency.

In addition, while the main text and Discussion have been appropriately revised, the Abstract appears largely unchanged from the original submission. It does not fully reflect the clarifications and refinements introduced during revision. I recommend a minor update to the Abstract to ensure consistency with the revised manuscript and to accurately represent the scope and conclusions of the study.

Subject to these minor revisions, I am satisfied with the quality and presentation of the manuscript.

Reviewer #3

(Remarks to the Author)
The authors have addressed all my comment. the manuscript, SI and available data and processing routines appear to be clear and robust. I recommend publication

Version 3:

Reviewer comments:

Reviewer #2

(Remarks to the Author)
The authors have thoroughly addressed all comments and suggestions raised in previous rounds of review. The revised manuscript, supporting information, and accompanying data and processing routines are clear, rigorous, and robust. I have no further concerns and fully support publication.

Open Access This Peer Review File is licensed under a Creative Commons Attribution 4.0 International License, which permits use, sharing, adaptation, distribution and reproduction in any medium or format, as long as you give appropriate credit to the original author(s) and the source, provide a link to the Creative Commons license, and indicate if changes were made.
In cases where reviewers are anonymous, credit should be given to 'Anonymous Referee' and the source.
The images or other third party material in this Peer Review File are included in the article's Creative Commons license, unless indicated otherwise in a credit line to the material. If material is not included in the article's Creative Commons license and your intended use is not permitted by statutory regulation or exceeds the permitted use, you will need to obtain permission directly from the copyright holder.
To view a copy of this license, visit https://creativecommons.org/licenses/by/4.0/

REVIEWER COMMENTS

Reviewer #1 (Remarks to the Author):

This study aims to understand the potential mechano-bactericidal and chemical effects of nanopillared calcium-deficient hydroxyapatite (CDHA) on Gram-positive Bacillus subtilis. The same group previously reported promising mechano-bactericidal activity of CDHA against Gram-negative Pseudomonas aeruginosa. The current study complements that work by examining the phenomenon with Gram-positive B. subtilis. It is well established in the mechano-bactericidal literature that Gram-positive bacteria have thicker cell envelopes and are therefore more resistant to nanotopography-mediated mechanical perturbation. In addition to using a Gram-positive rather than a Gram-negative species, another enhancement is the use of cryo-SXM to probe physical and chemical changes within cells in a near-native aqueous environment during interaction with various CDHA samples and controls. The authors reported up to 50% reduction achieved (CDHA with large pillars) in B. subtilis after 3-h static incubation in PBS.
*****Strength:**
This study presents solid, comprehensive data that enrich the bacteria-material interaction literature. The experiments are clearly well thought out, and the effort to present the whole picture is commendable. Thorough characterization is provided for the materials, the liquid environment, and the bacterial cells, and the main conclusions are supported by the data. A notable strength is the use of complementary techniques—fluorescence microscopy, ICP-MS, and XANES—to trace Ca^{2+} flow from CDHA to the surroundings and ultimately into the cytosol. Consistency between the fluorescence and XANES data strengthens their reliability. Cryo-SXM is still a relatively new method for studying bacteria–material interactions under close-to-native conditions – this study provides another solid case study of implementing these novel tools in understanding bacteria-material interactions.
*****Weakness:**
Although the study provides several new observations through cryo-SXM, it is unclear how these findings might help improve mechano-bactericidal efficacy against Gram-positive bacteria.

We appreciate the positive comments and the reviewer's recognition of the contributions of our work toward improving the understanding of bacteria–substrate interactions. In the responses to

the specific questions raised, which we develop below, we hope to highlight further the relevance of the results obtained and their potential contribution to the improvement of antimicrobial surface design.

-The authors reported up to 50% reduction achieved (CDHA with large pillars) in B. subtilis after 3-h static incubation in PBS. How does that compare with results achieved in prior studies using BS or other Gram+ bacteria?

We thank the reviewer for raising this important point. We agree that providing references on how different topographies perform against *B. subtilis* and other Gram-positive bacteria is highly relevant for the reader. We have expanded this information in the Discussion section and have included a detailed comparison with the existing literature on antimicrobial nanotopographies tested with *B. subtilis* and other Gram-positive bacteria such as *S. aureus*, and various species of *Lactobacillus,* in the Supplementary Information.

Discussion section:

"Bacterial adhesion experiments revealed increased mortality on nanostructured CDHA compared to flat controls (Fig. 2). *B. subtilis* exhibited less susceptibility compared to previous results with *P. aeruginosa* on similar topographies[7]. It is likely attributable to the thicker peptidoglycan layer of the Gram-positive bacterial wall, which confers higher structural strength. This trend was already observed on black silicon nanotopographies, which also reported a *B. subtilis* mortality lower compared to *P. aeruginosa.*[34,35] Detailed information on the performance of nanostructured topographies tested against *B. subtilis* and other Gram-positive bacterial strains is provided in Table S2 of the Supplementary Information."

Table S2. Summary of reported bactericidal efficacy of nanostructured surfaces against Gram-positive bacterial strains.

**Bacteria**	**Material**	**Incubation time**	**Reported mortality / reduction**	**Reference**
Bacillus subtilis	Black silicon	3 h	~60% mortality	[1]
Bacillus subtilis	Dragonfly wings (natural)	3 h	~50% lower than *P. aeruginosa* mortality	[2]
Bacillus subtilis	CDHA (this work)	3 h	59% mortality	–
Lactobacillus spp. (8-strain coculture)	Gecko skin replicas (chitosan, alginate, silk fibroin)	48 h – 7 days	Up to 95% (qualitative SEM analysis)	[3]
Staphylococcus aureus	Black silicon	18 h	~92% mortality	[1]
Staphylococcus aureus	Ormostamp polymer	2 h	97% mortality	[4]
Staphylococcus aureus	Silicon	3 h	86% mortality (also killed eukaryotic cells)	[5]
Staphylococcus aureus	Aluminium	24 h	Log reduction ~2.7	[6]

Staphylococcus aureus	Gold nanospikes	24 h	Log reduction ~3.7	7
Staphylococcus aureus	PMMA topographies	7 h	60% mortality	8
Staphylococcus aureus	Polyvinylidene	18 h	75% mortality	9
Staphylococcus aureus	Silicon	8 h	50% mortality	10
Staphylococcus aureus	Titanium	90 min	70% mortality	11
Staphylococcus aureus	Titanium	18 h	65% mortality	12
Staphylococcus aureus	Titanium	1 h	none	13
Staphylococcus aureus	Titanium	3 h	Real-Time Glo: 60% Bactiter Glo < 10%	14
Staphylococcus aureus	Titanium	2 h	~45%	15
Staphylococcus aureus	Titanium Oxide	3 h	~50%	16
Staphylococcus aureus	Titanium Oxide	18 h	19,8%	17
Staphylococcus aureus	Caloptery haemorrhoidalis and damselfly wings (natural)	3 h	26% mortality	18
Staphylococcus aureus	Block copolymer (polystyrene-block-poly(2-vinylpyridine))	3 h	~20% mortality	19
Staphylococcus aureus	Titanium oxide	1 h	No mortality	20
Staphylococcus aureus	Titanium oxide	2 h	< 20% mortality	21
Staphylococcus aureus	Black silicon	3 h	65% mortality	22

The most frequently studied Gram-positive strains in this context include *S. aureus* and *Lactobacillus spp*.

For *B. subtilis*, mortality rates of approximately 60% have been reported after 3 h of incubation on black silicon nanotopographies.[1] Another study, using natural dragonfly wings, compared bactericidal activity across different wing types, noting that *B. subtilis* mortality was roughly 50% lower than that observed for *P. aeruginosa*.[2] In a previous work with CaP nanotopographies, we observed ~86% mortality for *P. aeruginosa* under similar conditions.[23] Therefore, the ~59% mortality obtained in the present study for *B. subtilis* is consistent with values reported in the literature.

For *Lactobacillus spp.*, a coculture of eight oral strains was tested on bioinspired Gecko skin replicas made from chitosan, alginate, and silk fibroin. Mortality rates of up to 95% were reported after 48 h to 7 days of incubation, based on qualitative SEM image analysis.[3] However, the long incubation periods and qualitative assessment methods limit direct comparison with our 3 h quantitative assays.

For *S. aureus*, numerous studies have explored a wide range of synthetic and natural nanotopographies. High mortality rates have been reported in certain cases, such as 97% after 2 h on Ormostamp polymer[4] and ~92% after 18 h on black silicon,[1]. In general, nanotopographies fabricated on black silicon have shown strong bactericidal performance against *S. aureus*[5,22]. Other materials, including aluminium,[6] titanium,[11,12] gold,[7] and polymers such as PMMA,[8] or Polyvinylidene[9], have also yielded mortality rates exceeding 60%. Nonetheless, several studies have reported more modest reductions (< 50%) after incubation across different materials.[10,13–21] Overall, while effective bactericidal responses have been achieved on some nanostructured topographies, many reports still show limited efficacy. This supports the widely recognized challenge of eliminating Gram-positive bacteria, which are less susceptible to nanotopographic effects due to their thicker and mechanically stiffer cell envelope.

In summary, although direct comparison between our results and those from previous studies is constrained by differences in experimental conditions and incubation times, the findings presented here demonstrate bactericidal efficiencies that are consistent with current literature and underscore the inherent difficulty of designing effective nanotopographies against Gram-positive bacteria.

1. Ivanova, E. P. et al. Bactericidal activity of black silicon. Nat. Commun. 4, 1–7 (2013).
2. Mainwaring, D. E. et al. The nature of inherent bactericidal activity: Insights from the nanotopology of three species of dragonfly. Nanoscale 8, 6527–6534 (2016).
3. Green, D. W. et al. High Quality Bioreplication of Intricate Nanostructures from a Fragile Gecko Skin Surface with Bactericidal Properties. Sci. Rep. 7, 1–12 (2017).
4. Wu, S., Zuber, F., Maniura-Weber, K., Brugger, J. & Ren, Q. Nanostructured surface topographies have an effect on bactericidal activity. J. Nanobiotechnology 16, 1–9 (2018).
5. Hasan, J., Raj, S., Yadav, L. & Chatterjee, K. Engineering a nanostructured 'super surface' with superhydrophobic and superkilling properties. RSC Adv. 5, 44953–44959 (2015).
6. Buhl, S., Stich, A., Bulitta, C., Zapata, C. & Paniagua, S. Proof of the effectiveness of novel bioinspired antimicrobial nanostructures. Curr. Dir. Biomed. Eng. 10, 131–134 (2024).
7. Wu, S., Zuber, F., Brugger, J., Maniura-Weber, K. & Ren, Q. Antibacterial Au nanostructured surfaces. Nanoscale 8, 2620–2625 (2016).
8. Viela, F. et al. Nano-engineering safer-by-design nanoparticle based moth-eye mimetic bactericidal and cytocompatible polymer surfaces. RSC Adv. 8, 22606–22616 (2018).
9. Chen, C. et al. Bactericidal surfaces prepared by femtosecond laser patterning and layer-by-layer polyelectrolyte coating. J. Colloid Interface Sci. 575, 286–297 (2020).
10. Susarrey-Arce, A. et al. Bacterial viability on chemically modified silicon nanowire arrays. J. Mater. Chem. B 4, 3104–3112 (2016).
11. Izquierdo-Barba, I. et al. Nanocolumnar coatings with selective behavior towards osteoblast and Staphylococcus aureus proliferation. Acta Biomater. 15, 20–28 (2015).
12. Zhao, L. et al. Low-Temperature Hydrothermal Synthesis of Novel 3D Hybrid Nanostructures on Titanium Surface with Mechano-bactericidal Performance. ACS Biomater. Sci. Eng. 7, 2268–2278 (2021).
13. Sengstock, C. et al. Structure-related antibacterial activity of a titanium nanostructured surface fabricated by glancing angle sputter deposition. Nanotechnology 25, (2014).
14. Jenkins, J. et al. Antibacterial effects of nanopillar surfaces are mediated by cell impedance, penetration and induction of oxidative stress. Nat. Commun. 11, (2020).
15. Cao, Y. et al. Nanostructured titanium surfaces exhibit recalcitrance towards Staphylococcus epidermidis biofilm formation. Sci. Rep. 8, 1–13 (2018).
16. Jaggessar, A. et al. Mechanical, bactericidal and osteogenic behaviours of hydrothermally synthesised TiO 2 nanowire arrays. J. Mech. Behav. Biomed. Mater. 80, 311–319 (2018).
17. Bhadra, C. M. et al. Antibacterial titanium nano-patterned arrays inspired by dragonfly wings. Sci. Rep. 5, 1–12 (2015).
18. Truong, V. K. et al. The susceptibility of Staphylococcus aureus CIP 65.8 and Pseudomonas aeruginosa ATCC 9721 cells to the bactericidal action of nanostructured Calopteryx haemorrhoidalis damselfly wing surfaces. Appl. Microbiol. Biotechnol. 101, 4683–4690 (2017).
19. Fontelo, R., Soares da Costa, D., Reis, R. L., Novoa-Carballal, R. & Pashkuleva, I. Bactericidal nanopatterns generated by block copolymer self-assembly. Acta Biomater. 112, 174–181 (2020).
20. Diu, T. et al. Cicada-inspired cell-instructive nanopatterned arrays. Sci. Rep. 4, (2014).
21. Hizal, F. et al. Impact of 3D hierarchical nanostructures on the antibacterial efficacy of a bacteria-triggered self-defensive antibiotic coating. ACS Appl. Mater. Interfaces 7, 20304–20313 (2015).
22. Linklater, D. P., Nguyen, H. K. D., Bhadra, C. M., Juodkazis, S. & Ivanova, and E. P. Influence of nanoscale topology on bactericidal efficiency of black silicon surfaces. Nanotechnology 28, 9 (2017).

23. Iglesias-Fernandez, M., Buxadera-Palomero, J., Sadowska, J.-M., Espanol, M. & Ginebra, M.-P. Implementation of bactericidal topographies on biomimetic calcium phosphates and the potential effect of its reactivity. Biomater. Adv. 212797 (2022).

-I have concerns about "selection bias" in surveying and quantifying the intracellular Ca2+ concentration, which may be intrinsic to XANES technique itself. XANES only looked at the cells that are still intact and are dislodged from the material surfaces, following 40s sonication. The conclusions drawn on intracellular Ca2+ concentration are applicable only to these selected cells, but it is not discussed how representative that selected population was of the whole population on material surfaces in their native state. Take, for example, the ruptured cells (close 50% for L) – what were their Ca2+ level? Could they have had ruptured (but not their peers) because they had higher/lower intracellular Ca2+? They were completely excluded in the XANES analysis… Fluorescent data may help address this. A more detailed discussion comparing/contrasting the fluorescent and XANES results may help too.

We thank the reviewer for raising this question, which is undoubtedly relevant. We agree that the quantification of intracellular calcium by XANES is limited to bacteria that retain their cytosol and therefore have an intact or only slightly damaged cell wall. Nonetheless, this limitation is not exclusive to the XANES analysis; it is also present, to a similar extent, in the fluorescence assay. In fact, it is interesting to note the excellent correlation between the Fluo-4 results and the calcium maps. The number of bacteria visible in the latter (Figure 7b) is higher on the L and F substrates, which are also those showing the highest percentage of Fluo-4 AM–labelled cells (Figure 4b).

Certainly, as the reviewer points out, these two assays, as well as the calcium quantification performed using LAC, do not monitor the calcium levels that may have been present in ruptured bacteria and that could hypothetically have contributed to their rupture. However, even assuming that calcium concentration analysis is limited to bacteria with intact cell walls, if an increase in intracellular calcium concentration were a trigger for cell death, one would expect a correlation between the substrates showing higher intracellular calcium levels in the alive bacteria and those exhibiting greater mortality, which was not observed in this case. In fact, the highest intracellular calcium concentrations were found in bacteria cultured on the flat substrate, which was the one with lowest mortality rate.

Following the reviewer's suggestion, we have included these points in the Discussion section of the manuscript as follows:

"(…) we detected elevated calcium concentration homogeneously distributed in the cytosol of a fraction of the bacterial population, and Ca L-edge XANES spectra supported the presence of calcium in a soluble state. It is important to acknowledge that the assessment of intracellular calcium by XANES is restricted to bacteria that retain their cytosol and therefore have an intact or only slightly damaged cell wall. This limitation, however, is not exclusive to the XANES analysis; it similarly applies to the Fluo-4 AM assay. Interestingly, an excellent correlation was observed between the Fluo-4 fluorescence data and the calcium maps. The number of bacteria visible in the latter (Figure 7b) was higher on the L and F substrates, which also exhibited the highest percentage of Fluo-4 AM–labelled cells (Figure 4b), revealing an increase in calcium-rich bacterial cells, correlated with elevated Ca release from the underlying substrate. "
(…)

"While these results illustrate that ionic exchange between the substrate and surrounding fluids can influence the internal concentration of *B. subtilis*, our findings indicate that this process does not

directly impact its mortality. One could argue that both XANES calcium maps and Fluo4-AM assays, as well as the calcium quantification performed using LAC, do not monitor the calcium levels that may have been present in ruptured bacteria and that could hypothetically have contributed to their rupture. However, even assuming that calcium concentration analysis is limited to bacteria with intact cell walls, if an increase in intracellular calcium concentration were a trigger for cell death, one would expect a correlation between the substrates showing higher intracellular calcium levels in the alive bacteria and those exhibiting greater mortality, which was not observed in this case. In fact, the substrate exhibiting the highest ionic exchange capacity, the flat substrate (F), did not result in increased mortality rates compared to the control (Fig. 2b), suggesting that ionic exchange alone is not a determining factor in bacterial viability under the conditions tested. This observation is consistent with the known halotolerance of *B. subtilis*,[44,45] which enables adaptation to environments with elevated salinity.[46]"

-The authors convincingly show that cryo-SXT and XANES can resolve subcellular structures such as vesicle formation and Ca^{2+} accumulation; however, the biological significance of these observations, particularly in relation to mechano-bactericidal efficacy, need to be discussed in greater details.

We thank the reviewer for highlighting this point. We have further elaborated on the biological significance of cryo-SXT observations, expanding the Discussion as follows:

"(…) Cryo-SXT revealed bacteria with a low absorption contrast suggestive of cytosolic leakage and cell wall rupture on both nanostructured substrates (Fig. 5). Moreover, intracellular vesicles and multivesicular bodies, as well as endospore formation, were observed. In *B. subtilis*, the formation of vesicles and multivesicular bodies has been described as a pre-lytic remodelling response that can be triggered by cell envelope damage[39] or by autolytic mechanisms aimed at destroying severely compromised cells or serving as a decoy to protect neighbouring ones.[40] Nanopillared topographies have also been reported to induce both effects in *P. aeruginosa*.[22] Although cell envelope damage was evident in bacteria on both small and large pillars, vesiculation was detected exclusively in the S group, typically coinciding with reduced cytosolic contrast. Given the time- and resource-intensive nature of Cryo-SXT, only a limited number of cells (on the order of tens) could be analysed, precluding statistically robust quantification. Moreover, Cryo-SXT may not detect all vesicles, particularly in cells with intact or dense cytoplasm, due to resolution (~30 nm) and sensitivity limitations. Considering that bacterial viability was comparable on the S and L substrates, we cannot exclude the possibility that vesiculation also occurs on the latter. Notably, no correlation was found between vesiculation and elevated intracellular calcium levels, as a higher proportion of bacteria with increased calcium content was observed on the large-pillar and flat substrates."

-Can the authors formulate new hypotheses on how nanotopography and surface chemistry could be modified to enhance the bactericidal efficacy of CDHA—or similar materials—based on these observations? This is a major weakness of the study.

Thank you for this observation. To address this, we have introduced a paragraph in the Discussion section as follows:

"[…] This observation is consistent with the known halotolerance of *B. subtilis*,[44,45] which enables adaptation to environments with elevated salinity.[46]

Overall, this study highlights the dual role of substrate topography and chemistry in influencing bacterial viability. While the halotolerance of *B. subtilis* prevented mortality linked to ionic uptake in

this case, enhancing both the mechanical and chemical components could further improve the antimicrobial performance of the substrates. Refining nanotopographical parameters, such as nanopillar sharpness, may increase mechanobactericidal efficiency. In parallel, surface functionalization with antimicrobial peptides offers a complementary strategy.[47] Owing to their cationic nature, antimicrobial peptides strongly interact with negatively charged bacterial membranes, not only amplifying the mechanical damage induced by nanotopographies but also introducing additional electrostatic and biochemical routes for cell envelope disruption. This combined action can yield multimodal bactericidal surfaces with superior efficacy.[48]

Similarly, doping CaP-based topographies with antimicrobial ions could reinforce bactericidal effects through synergy between ionic toxicity and mechanical stress.[48] Although calcium showed no toxicity toward *B. subtilis* in this study, the observed ionic exchange between bacterial cells and the substrate reveals additional interaction pathways beyond mechanical damage. As metal ion accumulation is often central to toxicity, through membrane destabilization, intracellular interference, or ROS generation, leveraging these synergistic mechanisms may further magnify antimicrobial performance.[50]

In this context, Cryo-SXM emerges as a powerful, label-free tool for investigating bacteria-substrate interactions, providing high-resolution insight into both mechanical and ionic exchange processes. This dual analytical capability is particularly valuable for characterising multifunctional biomaterials with concurrent bioactive and bactericidal properties."

-While cryo-SXT and XANES are still fairly new in the field, these techniques have already been used to explore whole-cell ultrastructure (e.g., doi: 10.1042/ETLS20170086). This raises the bar for novelty, and demonstrating biological significance or proposing material-engineering insights derived from these techniques is essential to appeal to the readership of Nature Communications.

We thank the reviewer for raising this point regarding novelty. We believe that the value of this study lies not only in uncovering new biological insights into the poorly understood bacteria-substrate interactions on antimicrobial nanotopographies, but also in demonstrating the broad applicability of synchrotron cryo-soft X-ray techniques to health research and microbiology.

As correctly mentioned by the reviewer, cryo-SXT has previously been applied to study cellular ultrastructure; however, the vast majority of published work has focused on eukaryotic cells. Applications to bacteria remain scarce, mainly due to the technical challenges imposed by their small size and the sensitivity required for detecting ionic elements. The paper mentioned (doi: 10.1042/ETLS20170086) reports cryo soft X-ray tomography data, without including Ca chemical speciation or cryo-XANES analysis. To the best of our knowledge, our study is the first to report intracellular Ca^{2+} characterization in bacteria using cryo-SXT combined with cryo-XANES. Previous publications from the MISTRAL beamline (*e.g., Sviben et al., Nat. Commun. 7,1-9, 2016; Gal et al., PNAS 115, 11000-05, 2018; Kahil et al., PNAS 117, 30957-65, 2020; Sorrentino et al., IJMS 22, 2021; Rossi et al., IJMS 24, 2023*) demonstrated the power of combining tomography with XANES, but none investigated bacterial cells. Moreover, those works primarily characterized Ca in solid compounds of comparable size to entire cells, whereas our study focuses on Ca in ionic form within the intracellular environment, where the lower atomic density makes detection and quantification substantially more challenging.

Finally, we would like to emphasize that our work goes beyond illustrative spectra: by pairing XANES with linear absorption coefficient (LAC) quantification from cryo-tomography, we derived quantitative intracellular Ca^{2+} concentrations at the single-cell level. This constitutes an advance both in

methodology and in application. The novelty of our work therefore resides in (i) applying cryo-SXR to bacterial systems, (ii) integrating imaging and spectroscopy, and (iii) obtaining quantitative ionic Ca^{2+} values per bacterium, an achievement not reported in previous studies. Furthermore, this methodological approach could be extended to monitor other biologically relevant elements, provided that their absorption edges lie within the water window.

We have summarized these aspects and included them at the end of the Discussion section as follows:

"In this context, Cryo-SXM emerges as a powerful, label-free tool for investigating bacteria-substrate interactions, providing high-resolution insight into both mechanical and ionic exchange processes. By combining cryo-SXT with cryo-XANES, we achieved, for the first time, quantitative intracellular characterization of ionic Ca^{2+} in bacteria at the single-cell level. This integrative approach, linking imaging, spectroscopy, and quantitative analysis, opens new avenues for exploring elemental dynamics in microorganisms and could be extended to other biologically relevant elements within the water window.[51] This dual analytical capability is particularly valuable for characterising multifunctional biomaterials with concurrent bioactive and bactericidal properties."

Reviewer #2 (Remarks to the Author):

General Assessment:

The work presented here is original and addresses a timely topic—the need for antibiotic-free antibacterial surfaces to mitigate antibiotic resistance. The authors combine advanced materials synthesis (CDHA nanopillars) with state-of-the-art imaging techniques (Cryo-SXT and XANES) to investigate bacteria–surface interactions. However, significant methodological flaws raise concerns about the validity of the conclusions. Below, I provide a structured review highlighting both strengths and critical weaknesses that must be addressed to make this work suitable for publication.

Novelty and Impact

The study is original and interdisciplinary, bringing together materials science, microbiology, and advanced imaging.

The application of Cryo-SXT and XANES is innovative for analyzing bacterial interactions with surfaces and Ca incorporation.

However, critical methodological flaws in sample preparation and data analysis compromise the validity of key conclusions. These issues are detailed in subsequent sections.

Does the Work Support the Conclusions and Claims?

Currently, the data do not robustly support the main claims, particularly regarding the bactericidal effect of the surfaces and the role of calcium uptake. Specific concerns:

The Live/Dead staining results are invalid due to fixation prior to staining.

We repeated the Live/Dead assays following the reviewer's recommendation, performing the staining on unfixed samples as requested.

SEM sample preparation leads to artifacts (e.g., cell collapse), rendering morphological interpretations unreliable.

We repeated the SEM observations using an alternative preparation protocol that minimizes dehydration artifacts, as suggested.

Sonication-based sample preparation likely detaches or damages both bacteria and nanostructures, undermining claims about cell wall damage localization.

We performed additional tests to verify that the sample preparation protocol did not introduce extra damage to the bacteria, confirming that the observed effects were not artifacts of sonication.

No direct evidence of bacterial viability (e.g. CFU counts) is presented.

We conducted additional CFU and metabolic assays to directly quantify bacterial viability, as recommended.

Comment on bone integration of the material are made without supporting data.

We included additional information and previously reported data supporting the bone integration capacity of these substrates

A detailed description of the actions taken to address each of these points is provided below.

3. Major Flaws / Drawbacks in the Experimental Setup

3.1 Live/Dead Staining

Critical Flaw: The cells are fixed before Live/Dead staining. This renders the assay invalid, as fixed cells are no longer viable and cannot accurately differentiate between live and dead populations. Please repeat these experiments using a fixative-compatible Live/Dead protocol or alternative method that preserves viability.

We thank the reviewer for this valuable comment. Following the reviewer's advice, we repeated the Live/Dead assays, performing the staining on unfixed samples as requested. Moreover, to obtain more robust results and complementary information on bacterial viability, we also carried out CFU assays and measured metabolic activity using luminescence (BacTiter-Glo).

The results were included in a new version of Figure 2. Confocal Live/Dead images without fixing bacteria prior to the staining protocol is shown in Figure 2a. Mortality was quantified from confocal images, revealing mortality rates of 55.28% on the S topography and 58.86% on the L topography, compared to 16.7% on F and 9.04% on TCPS (Ctrl). These values are comparable to those obtained using the previous protocol and follow the same trend. Consistently, Bactiter-Glo assays indicated a reduction in metabolic activity on the nanotopographies relative to the flat substrate. CFU counts further confirmed this tendency, showing Log_{10}(fold change) reductions of 0.63 (S) and 0.81 (L) compared to F.

The updated version of Figure 2, together with the revised experimental methods, incorporating the changes made to the Live/Dead protocol as well as the metabolic and CFU assays, are presented below. For the CFU experiments, which required detaching the cells from the substrate, we employed the same sonication procedure used for cryo-SXT sample preparation to ensure reproducibility of the results. This procedure is described in detail later in this document. Statistical analysis was updated with the Mixed effects model used for CFUs.

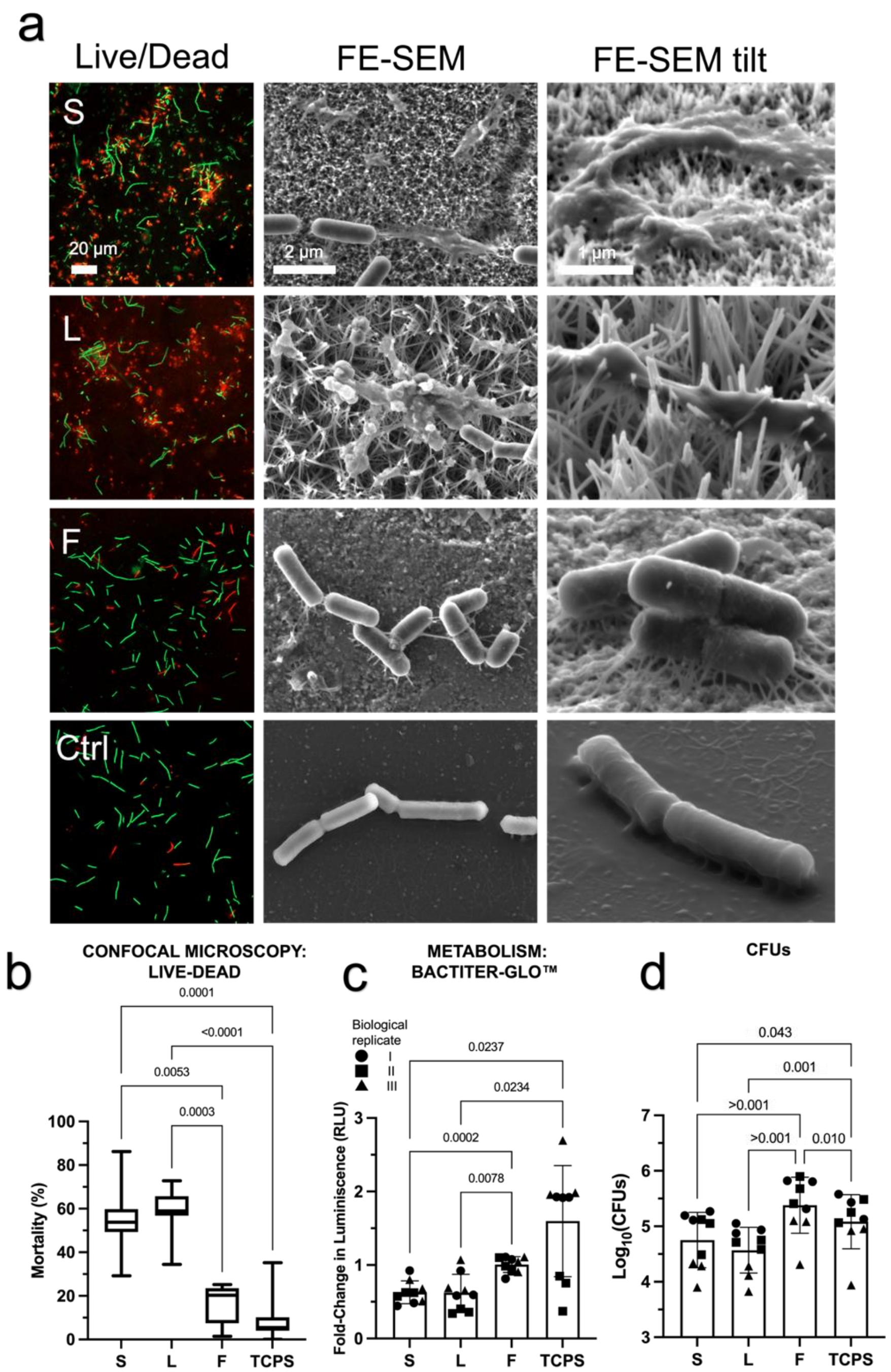

Figure 2. B. subtilis mortality on the nanostructured CDHA topographies. *Bacteria were incubated on the different substrates for 3 hours: nanostructured CDHA substrates with small and large nanopillars (S and L respectively), flat CDHA (F) and glass coverslips (Ctrl). a) First column: CSLM images of bacteria after Live/Dead BacLight staining. Red identifies dead bacteria, while green denotes viable cells. Second and third columns: Top-view and tilted FE-SEM micrographs showing bacteria adhesion on different substrates; b) Boxplot displaying ~~Quantification of~~ bacteria mortality, expressed as the percentage of dead cells relative to the total population adhered, obtained from CSLM images. Box plots indicate median (middle line), 25th, 75th percentile (box) and 5th and 95th percentile (whiskers). No outliers were observed. c) Bacterial viability assessment by metabolic activity (Bactiter-Glo) normalized to the F control. d) Bacterial viability assessment by CFUs counting of the total viable bacteria adhered to the topographies. For c) and d) data are presented as mean ± standard deviation and dot shape indicate the corresponding biological replicate. For Live/Daad mortality (b) and Bactiter-Glo™ (c), statistically significant differences were determined using One-way ANOVA. For CFU counting (d) statistically significant differences were determined using a mixed-effects model. P-Values are given within the graph.*

Results

(…)

"The bactericidal effect was significantly higher on both the small and large nanopillars than on the flat surface or glass coverslip controls (Ctrl), as evidenced by the increased presence of red-stained bacteria dead bacteria. No statistically significant differences in mortality were found between the two nanostructured topographies (L and S), nor between the two flat surfaces (F and Ctrl) (Fig. 2b). In agreement with these findings, Bactiter-Glo assays indicated a significant reduction in metabolic activity on the nanotopographies compared with the flat substrate (Fig. 2c). CFU analysis further supported this trend, confirming a reduction in bacterial viability on the nanostructured surfaces relative to the flat control (Fig. 2d)."

Materials and methods

"Antibacterial activity

(…)

The samples were then ~~fixed with 500 µL of 2.5% glutaraldehyde in PBS for 30 min at 4° C, washed with PBS three times and~~ stained using LIVE/DEAD® BacLight™ Bacterial Viability Kit (Invitrogen, Cat. No. L7012, USA). The staining solution was prepared by diluting 2,5 µL of the mixture of 1.67 mM SYTO9 and 18.3 mM Propidium Iodide (PI) per 1 mL of PBS.

(…)

Metabolic Activity Assay

Metabolic activity of adherent bacteria was quantified using the BacTiter-Glo™ assay (Promega). Following the adhesion experiment, CDHA discs were incubated for 5 min in a 1:1 mixture of phosphate-buffered saline (PBS) and BacTiter-Glo™ reagent. The resulting luminescent solution from each disc was transferred to an opaque white 96-well plate (Nunc™ MicroWell™ Flat-Bottom, Thermo Fisher Scientific, cat. no. 136101), and luminescence was recorded immediately using a plate reader (Tecan. Infinite® M Plex multimode microplate reader, Tecan Group Ltd.). Experiments were performed independently on three separate days (n=3), and 3 technical replicates were used per condition (n=9).

Colony-forming unit (CFU) enumeration

For CFU determination, CDHA discs were first washed three times with PBS to remove non-adherent bacteria. Bacteria were detached using the same sonication procedure used for cryo-SXT sample preparation to ensure reproducibility of the results. The protocol is described in detail in that section and illustrated in Fig. S5. The resulting suspension was diluted by transferring 20 µL into 180 µL of PBS. Serial tenfold dilutions of these suspensions were plated on Mueller–Hinton agar (Scharlau, cat. no. 02-136-500) using the Miles and Misra method and incubated overnight at room temperature. Colonies were enumerated the following day. Mueller–Hinton agar was prepared according to the manufacturer's instructions.

Statistical analysis

Normality of data was assessed before analyses. Statistical significance was determined using either 1-sample t Test, Kruskal-Wallis test, one-way or two-way ANOVA, and mixed-effects model for CFUs counting, as specified in each Figure legend. Statistically significant differences were considered for

two-tailed p-value < 0.05, and p-value < 0.01 for ΔLAC. Statistical analyses were performed in Graph Pad Prism 10.2.3, and mixed-effects model on Minitab 19.

3.2 SEM Sample Preparation

After dehydration, samples are coated with carbon. This procedure induces shrinkage and collapse artifacts, distorting the bacterial morphology. For accurate bacterial visualization, critical point drying (CPD) or HMDS drying must be used. Please repeat these experiments using an appropriate protocol.

We thank the reviewer for this valuable suggestion. Following the reviewer's advice, we repeated the SEM sample preparation using HMDS drying after ethanol dehydration. Specifically, we applied HMDS/ethanol mixtures at ratios of 1:3, 1:1, and 3:1 for 15 min each, followed by three washes in 100% HMDS for 15 min. Newly acquired SEM images obtained with this protocol are now included in the revised manuscript (Fig. 2a, please see the response to the previous comment), and the Materials and Methods section has been updated accordingly.

Materials and methods

(…)

SEM bacteria preparation

Following confocal imaging, ~~the samples~~ bacteria were fixed with 500 µL of 2.5% glutaraldehyde in PBS overnight at 4˚ C, washed with PBS three times and dehydrated through a graded series of aqueous ethanol solutions (50%, 70%, 90%, 96%, and 100% twice, Ethanol Absolute Pure, 141086, PanReac Applichem), each step lasting for 10 min. Following ethanol dehydration, graded series of HMDS (1,1,1,3,3,3-Hexamethyldisilazane 98%, thermoscientific, Cat. No: 120585000) with ethanol solutions (33.33%, 50%, 66,67%, and 100% three times) were applied for 15 min each. Dried samples were subsequently coated with carbon sputter to minimize charging effects. Images were obtained on a Tescan Amber field-emission microscope (Tescan, Brno, Czech Republic) operated at an accelerating voltage of 5 kV and were acquired at zenithal and oblique views (55° tilt).

3.3 Sonication Procedure

The manuscript lacks detail about the sonication step:

What type of sonication was performed, and what energy was used? Sonication requires the sample to be immersed in liquid, yet 40 µL is insufficient to fully immerse the samples. Please clarify this procedure in the Methods section.

We thank the reviewer for raising this important point. Bacterial detachment presented a particular challenge, as the preparation of cryo-SXR TEM grids required obtaining a bacterial suspension with a sufficient concentration (approximately $OD_{600} \approx 0.3$). To achieve this, it was necessary to minimize the volume of PBS used. After several optimization trials, we determined that a volume of 40 µL was adequate to fully immerse the small discs used in our experiments, as illustrated in the representative image provided. Sonication was performed in an ultrasonic bath (Sonorex Digitec DT 52, Bandelin, Germany) operating at a nominal power of 60 W.

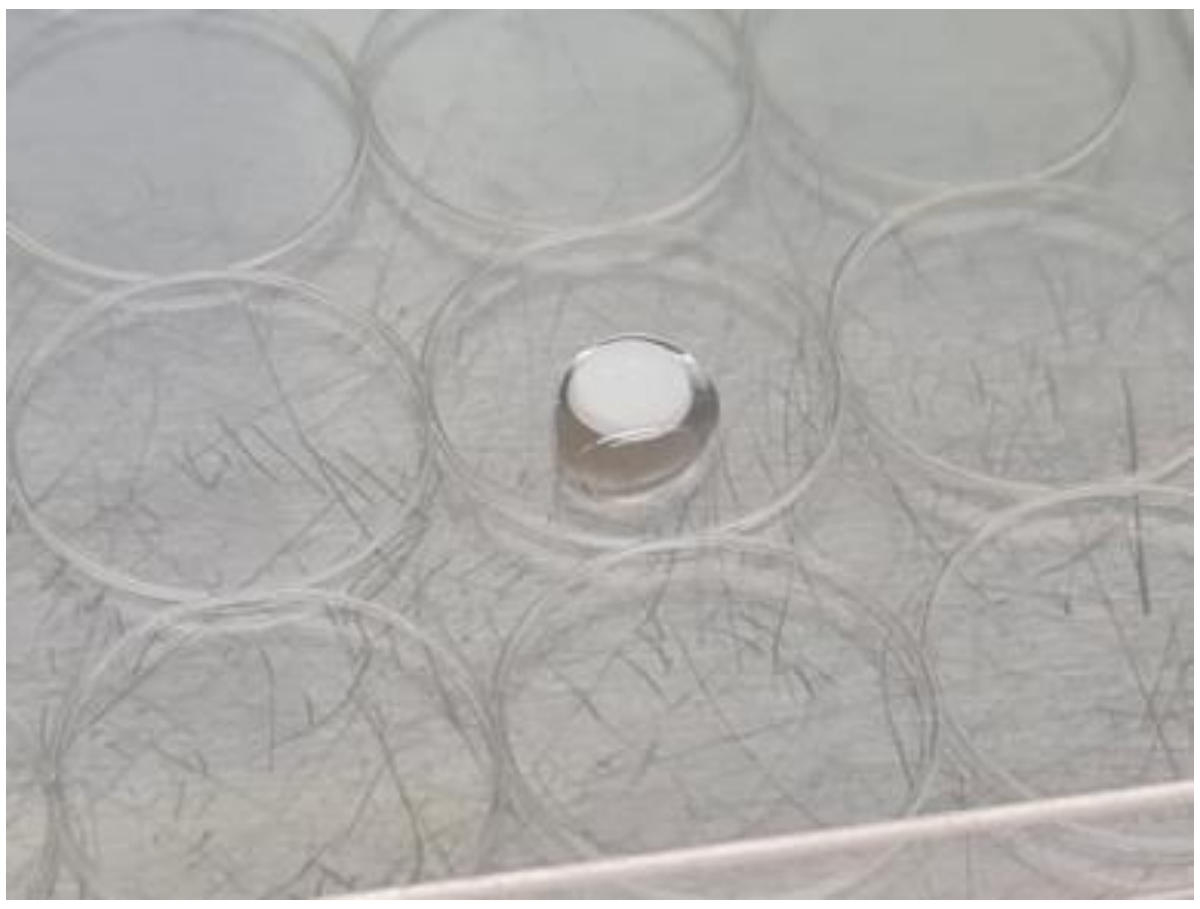

We have provided additional details on the protocol in the revised Materials and Methods section and a schematic illustration has also been included as Supplementary Information (Fig. S5).

"Sample Preparation for Cryo-SXT

~~A 3 h~~ An adhesion experiment was performed as previously described by incubating three technical replicates per condition and three empty wells as controls for 3h. Following incubation, the discs were washed three times with PBS to remove non-adherent bacteria. To obtain a concentrated bacterial suspension suitable for cryo-SXR grid preparation (OD_{600} > 0.3), each disc was placed face-down onto a 40 µL drop of PBS placed on the inner surface of a 24-well plate lid. The lid was then floated on the water surface of an ultrasonic bath (Sonorex Digitec DT 52, Bandelin; nominal power 60 W), and sonicated for 30 s (Fig. S5). 10 µL of the obtained bacterial suspensions were mixed with 1 µL of 100 nm gold fiducial markers (GC100, BBI solutions) and deposited onto the carbon side of Holey carbon-coated Quantifoil® R2/2 G200F1 finder grids (Quantifoil Micro Tools, Groβlobichau, Germany), which were previously glow-discharged using a Leica ACE200 to favor bacteria attachment to the grid. (…)"

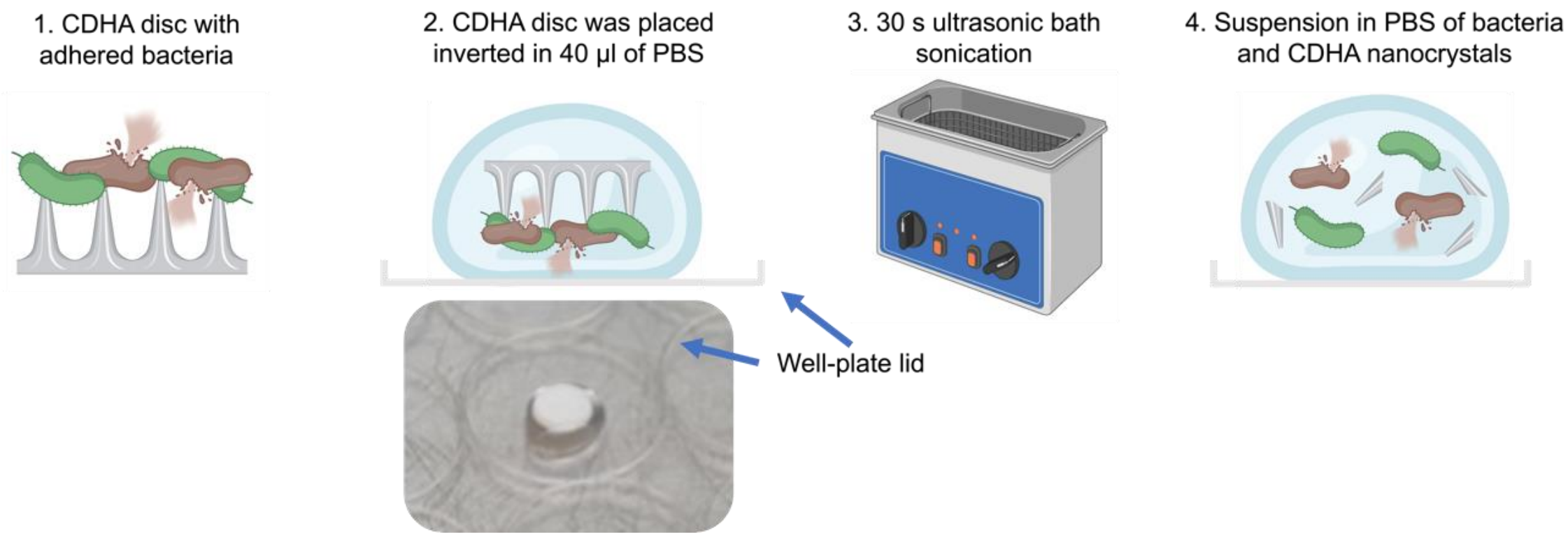

Figure S5. Schematic representation of the bacterial detachment protocol. *(1) CDHA substrates were washed three times with PBS to remove non-adherent bacteria. (2) Each disc was placed face-down onto a 40 µL drop of PBS positioned on the inner surface of a 24-well plate lid. (3) The lid was then floated on the water surface of an ultrasonic bath (Sonorex Digitec DT 52, Bandelin; nominal power 60 W) and sonicated for 30 s, releasing adherent bacteria together with CDHA nanocrystals into suspension (4).*

Additionally, 30-second sonication in ethanol reportedly caused nanopillar detachment from base—this suggests the structures/fabricated material are unstable for their intended application as implant coatings. How would these structures withstand clinical handling or in vivo conditions? Please address this concern regarding translational potential.

We thank the reviewer for this important comment regarding translational potential. We agree that the current form of these nanostructures may not be optimal for direct use as implant coatings since their mechanical stability may be compromised under high loading conditions. However, the material developed in this work is not intended as a coating for e.g. a metallic implant, but rather as synthetic bone grafts based on CDHA with antimicrobial topographies. It could be developed for instance in the form of granules or scaffolds, as we described for instance in *Raymond et al., Clinical Oral Investigations (2021) 25, 6695–706* or *Mateu-Sanz et al., Adv. Funct. Mater. (2025), e09357*. These applications are highly relevant for bone regeneration and oral surgery, where infection remains a major clinical challenge. In this context, we do not expect them to undergo frictional forces comparable to those acting on metallic implant coatings, such as in dental implants or articular prostheses. We have clarified this intended clinical application in the Introduction:

"Calcium Phosphates (CaPs) are the most widely used synthetic bone grafts to treat bone defects caused by trauma, disease, surgery, or congenital malformations. Among them, hydroxyapatite and calcium-deficient hydroxyapatite (CDHA) are particularly favored for bone tissue regeneration due to their biocompatibility, osseointegration, and osteoconductive properties[1]. These synthetic grafts can be engineered in various forms, such as granules, blocks, or porous scaffolds, tailored for applications in bone filling or bone augmentation. However, clinical procedures involving their application are not without risks. Postoperative infections can pose a challenge compromising the success of bone grafts.[2] The current standard treatment for preventing or treating such infections is antibiotic administration. However, the increasing antibiotic resistance calls for novel alternatives to combat biomaterial-associated infections.[3] "

3.4 Sample Preparation for Cryo-SXT

The authors aim to study bacterial cell wall damage relative to the position of nanopillars. However, using sonication to detach bacteria removes them from the surface, so their spatial relationship to the nanopillars is lost. This critically undermines the study's objective.

We thank the reviewer for this valuable observation and the opportunity to clarify our objective. The aim of our study was not to explicitly investigate bacterial cell wall damage in relation to the precise geometric position of the nanopillars. Instead, our goal was to assess the extent and nature of cellular damage induced by different topographies and to characterize the corresponding biological responses and subcellular morphology of bacteria after incubation. Recovering bacteria through sonication enabled us to examine both intact and damaged cells under various conditions and compare their ultrastructure and chemical speciation. We were therefore less focused on the exact spatial positioning of cells relative to the nanopillars, and more interested in understanding how exposure to different CDHA nanotopographies influenced bacterial morphology, subcellular organization and calcium internalization.

Moreover, if bacteria are strongly attached (as stated), detaching them with sonication could damage the cells, leading to artifacts in the Cryo-SXT images.

We thank the reviewer for raising this important concern. We agree that it is reasonable to consider that sonication could, in principle, compromise bacterial integrity and potentially induce artifacts. In this regard, the additional assays included in this revision, comprising: i) metabolic activity measurements (Bactiter-Glo) performed on attached bacteria, and ii) CFU counts of bacteria detached following the sonication protocol used for Cryo-SXT sample preparation, provide highly relevant information (Fig. 2). The viability trends observed in both assays were consistent with each other, as well as with the live/dead staining results obtained for bacteria adhered to the substrates also shown in Fig. 2. Together, these findings confirm that the sonication procedure did not compromise bacterial viability or alter the integrity of the cells.

To further address this point, we performed an additional experiment to assess whether the sonication protocol affected bacterial mortality. Live/dead confocal imaging (without glutaraldehyde fixation) was conducted on bacteria detached from the topographies using the same sonication procedure. Quantitative analysis showed no significant differences in mortality between bacteria adhered to the substrates or detached by sonication, across all tested topographies. These results confirm that the sonication step did not compromise bacterial integrity, supporting the validity of the Cryo-SXT observations.

We have included this information in the Discussion section and in Fig. S4 of the Supplementary Information:

"Discussion

(…)

Notably, FE-SEM images evidenced the presence of severely damaged bacteria on the nanostructured surfaces, and Synchrotron Cryo-SXT provided very relevant information in this respect, as it allows imaging whole bacteria in the hydrated state with minimal pre-processing beyond sonication and vitrification. Because images are acquired in transmission mode, detachment of bacteria from the substrate is required. While this represents a methodological limitation for biomaterial studies, the strong agreement between metabolic and Live/Dead assays on attached bacteria and the CFU counts of bacteria detached by sonication (Fig. 2) confirms that the sample preparation does not affect bacterial viability or mortality, ensuring the reliability of the observed effects. Furthermore, as shown in Fig. S4, Live/Dead confocal imaging of bacteria detached using the same protocol revealed no significant differences in mortality between adhered and detached bacteria across all tested topographies."

Supplementary Information

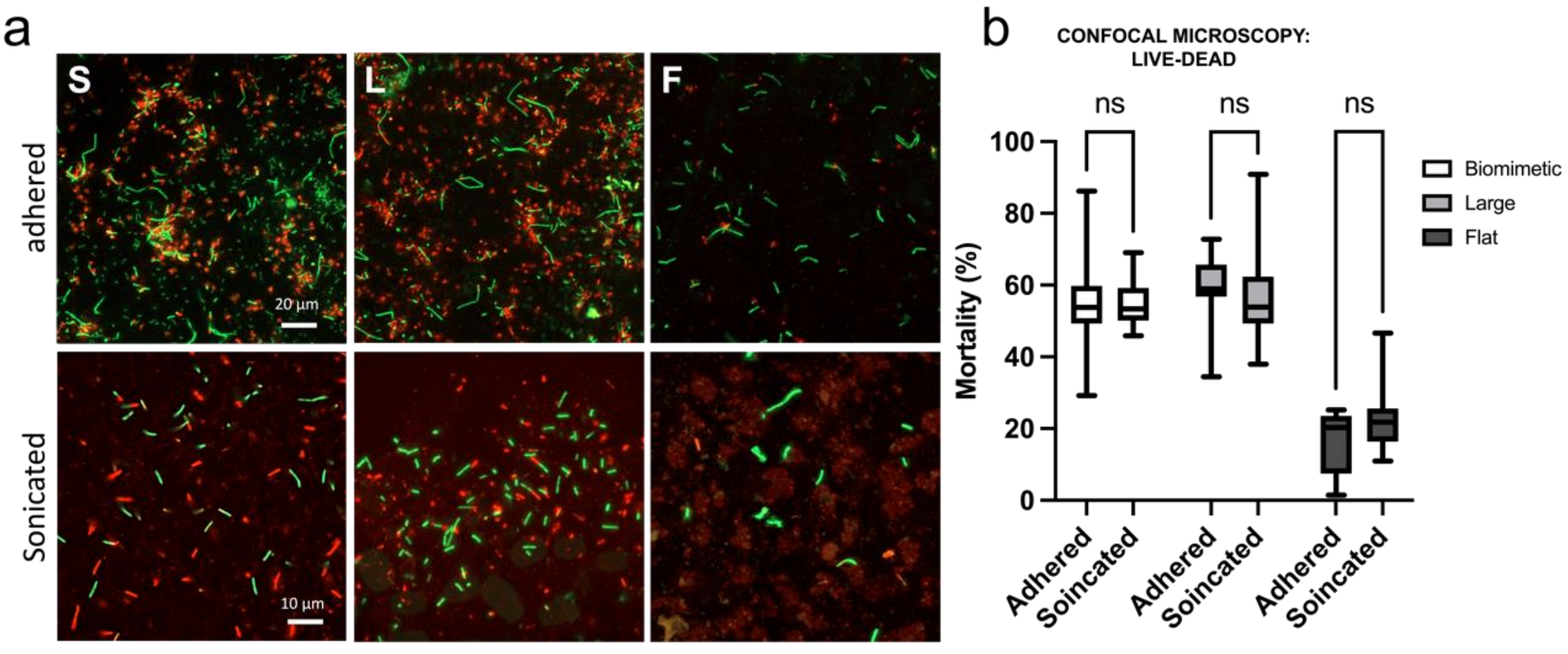

Figure S1. Assessment of the impact of the sonication protocol (30 s in 40 µL PBS) on bacterial viability and mortality. *To assess whether sonication affected bacteria mortality, we compared Live/Dead staining of bacteria adhered to the topographies with those detached following the sonication protocol. Live/Dead staining of detached bacteria was performed as follows: each PBS suspension of sonicated bacteria was placed on a confocal cover lid, and 0.5 µL of Live/Dead reagent was added directly to each suspension. Images were acquired after 7 min incubation and up to a maximum of 15 min, in accordance with the manufacturer's recommended incubation window. (a) Confocal images of adhered (top row) and sonicated (bottom row) bacteria from Small (S), Large (L), and Flat (F) topographies. Notably, the red background of sonicated bacteria corresponds to CDHA crystals. (b) Boxplots of mortality of sonicated and adhered bacteria after 3 h (n=12). Box plots indicate median (middle line), 25th, 75th percentile (box) and 5th and 95th percentile (whiskers). No outliers were observed.*

Notably, the TEM sample preparation shows that sonication for 30 seconds caused detachment of the nanopillars themselves. Why are nanopillars not observed in association with bacterial cells in Cryo-SXT?

We thank the reviewer for this comment and for the opportunity to clarify. Identifying detached nanopillars in Cryo-SXT images is not straightforward due to their nanoscale dimensions and the contrast limitations of the technique. However, detached pillars can indeed be observed in our datasets. Nanopillars are indicated in Figure 5 of the submitted manuscript with an asterisk (*).

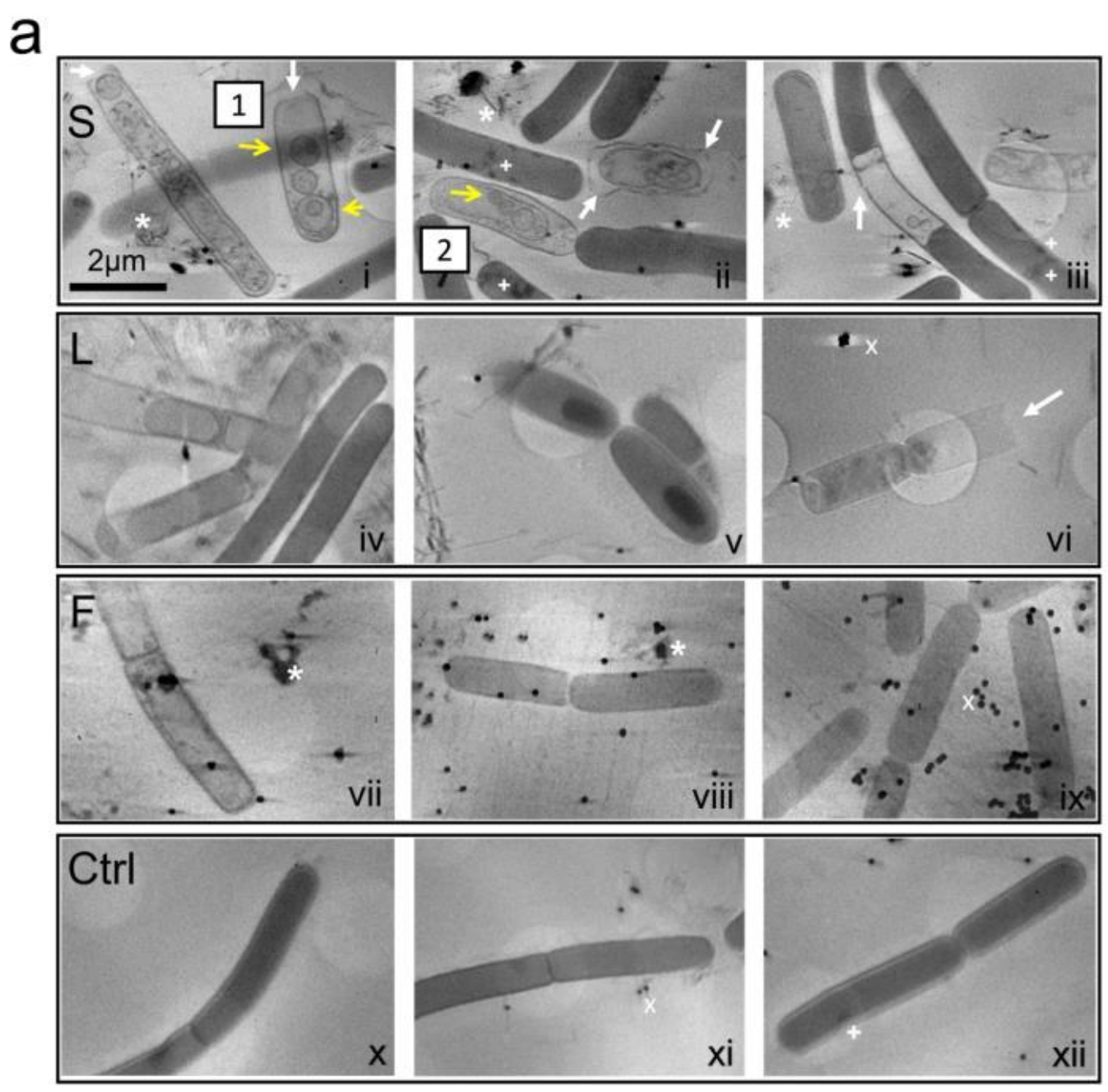

Figure 5. Bacteria ultrastructure and 3D segmentation of B. subtilis incubated on CDHA substrates or in the culture well. *a) Virtual slices of reconstructed tomographies acquired at 520 eV of vitrified bacteria after 3h incubation on CDHA substrates with small pillars (**i-iii**), large pillars (**iv-vi**), flat CDHA (**vii-ix**) and without substrate (**x-xii**). Images show bacterial cells, CDHA crystals (*), gold fiducials (x), and some circular holes in the background from the support film. White arrows indicate disrupted cell walls.(…)*

They are also visible in the calcium maps (Fig. 7a), in in particular to the images of the Large pillar condition (L), owing to their greater dimensions compared to the smaller-pillar conditions.

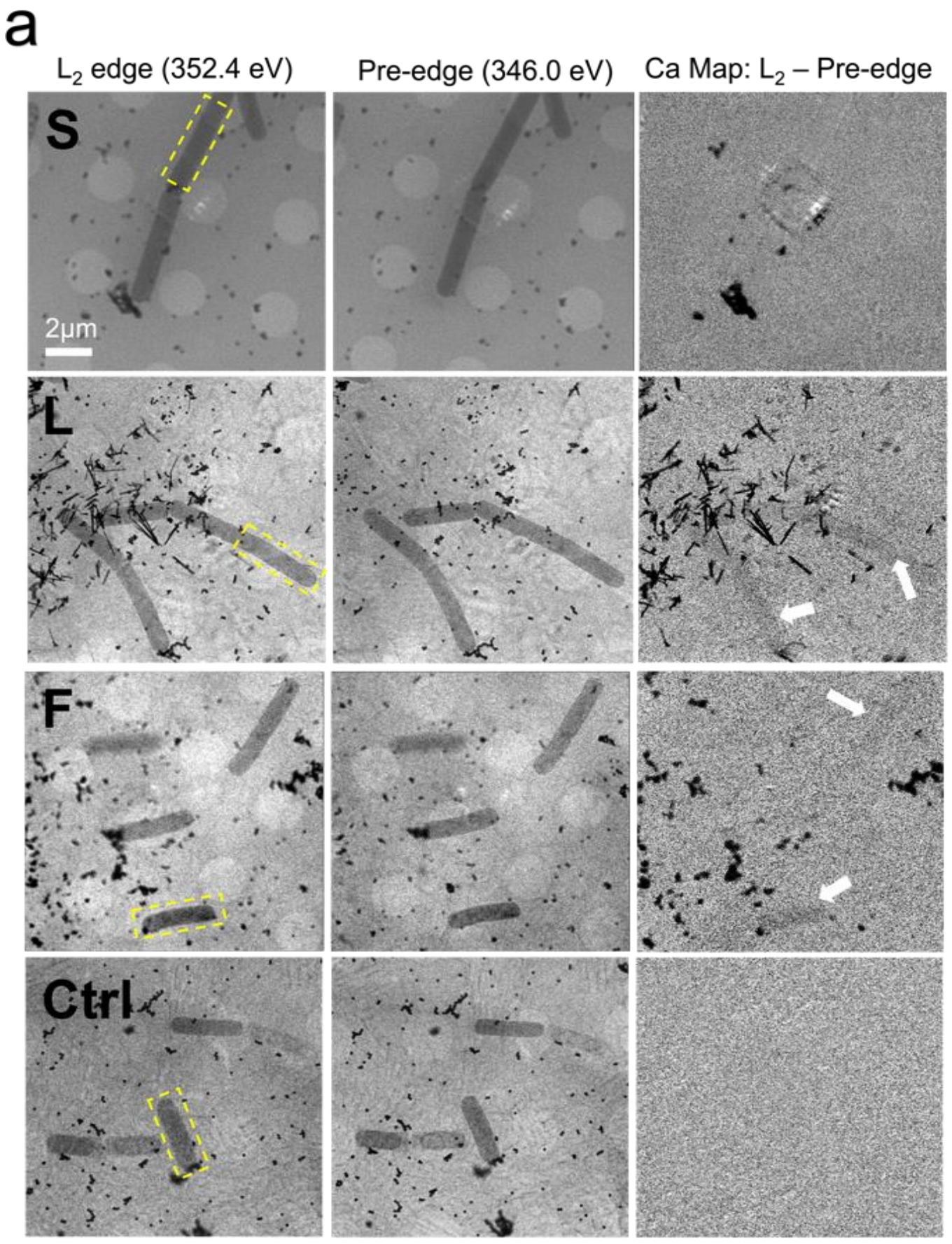

Figure 7. Calcium maps and XANES spectromicroscopy of bacteria incubated on CDHA substrates (S, L, F) or in the culture well (Ctrl). *a) Normalized projection images of bacteria acquired at the Ca L_2-edge (first column), the pre-edge (second column) and Ca maps generated by subtracting the pre-edge from the Ca L_2–edge image (third column). White arrows indicate bacteria exhibiting Ca signal in the Ca maps. Corresponding XANES spectra at the Ca $L_{2,3}$-edges were extracted from the cytosol of the bacteria outlined with a yellow dashed rectangle in the Ca L_2-edge images (right graphs). (…)*

Since cells are removed from the actual surface, this condition is no longer "near-native." Please update the manuscript to reflect this limitation and discuss its impact.

We thank the reviewer for raising this point. The term near-native was used to indicate that the cells do not need to be dehydrated, cut or stained to be observed by CRYO-SXT. However, we understand that it can be misleading as, as the reviewer noted, the bacteria have been detached and are

observed aside of the topography. We have removed this term from the abstract and have reflected this limitation and its impact in the Discussion section, as mentioned in the response to a previous Reviewer's comment:

"Abstract

[…] simultaneously visualising mechanical damage and intracellular ionic changes under whole hydrated~~, near-native conditions~~ state at subcellular level. Nanotopographies […]"

"Discussion

(…)

Notably, FE-SEM images evidenced the presence of severely damaged bacteria on the nanostructured surfaces, and Synchrotron Cryo-SXT provided very relevant information in this respect, as it allows imaging whole bacteria in the hydrated state with minimal pre-processing beyond sonication and vitrification. Because images are acquired in transmission mode, detachment of bacteria from the substrate is required. While this represents a methodological limitation for biomaterial studies, the strong agreement between metabolic and Live/Dead assays on attached bacteria and the CFU counts of bacteria detached by sonication (Fig. 2) confirms that the sample preparation does not affect bacterial viability or mortality, ensuring the reliability of the observed effects. Furthermore, as shown in Fig. S4, Live/Dead confocal imaging of bacteria detached using the same protocol revealed no significant differences in mortality between adhered and detached bacteria across all tested topographies."

3.5 Cryo-SXT Observations

Figure 5a (L vi) shows cells without a visible cell wall, yet they maintain their shape. How is this possible? Please clarify.

We thank the reviewer for pointing this out. In Fig. 5a (L vi), the right-hand side of the bacterium shows a discontinuity where the envelope appears absent. We interpret this as the rupture site through which cytosol was released, resulting in a reduced absorption contrast. The remaining cell wall, however, is sufficiently intact to preserve the overall shape of the bacterium. A similar situation is observed in Fig. 5a (i) and Fig. 5b (xiii-xv). Therefore, these cells are not wall-less, but rather represent partially ruptured bacteria with cytosol loss, which affects the visibility of their envelope in Cryo-SXT images.

4. Additional Data Analysis Concerns

4.1 Data Interpretation

The authors claim that only small nanopillars cause vesicle formation, but do not provide robust quantitative evidence to support this claim. What evidence you can provide for other cells, that similar vesicles are not present? Even in Figure 5a-i ii, iii darker cells do not show vesicles. Can this be a limitation of the technique?

We thank the reviewer for this insightful comment. The vesicles are associated with cellular stress. Indeed, vesicle formation is observed only in a subset of cells, primarily those with compromised cell walls and reduced absorption. In contrast, the darker, more absorbing cells retain intact membranes

and cytoplasm in terms of morphology at the resolution of the technique. In Fig. 5a (iii), vesicles are visible in a less absorbing cell in the upper left corner, likely reflecting cellular stress. The lighter cells have either lost cytoplasmic content or contain chemically altered material, resulting in lower absorption; these represent the most damaged cells and are also the ones exhibiting vesiculation.

Not all bacteria are expected to exhibit vesicle formation consistently with Live/Dead assays, which showed that approximately 40% of the bacterial population remained viable after adhesion to the topography. These viable bacteria would not be expected to display vesicle formation. Regarding potential technique limitations, we acknowledge that Cryo-SXT may not capture all vesicles in every cell, particularly in those with intact cytoplasm, due to resolution constraints or the possibility that high cytoplasmic density could mask vesicle formation. In any case, due to the time-consuming and resource-intensive nature of the technique, only a small number of bacteria were examined, making it difficult to draw statistically significant data. What we can conclude is that vesiculation, which is associated with cellular stress, was observed only in bacteria on the S surface, generally coinciding with reduced cytosolic contrast. Considering the cell viability results, which are similar for the S and L substrates, we currently have no evidence to rule out the possibility that vesiculation may also occur on the L substrates.

The Discussion section has been expanded to reflect these arguments.

"(…) Cryo-SXT revealed bacteria with a low absorption contrast suggestive of cytosolic leakage and cell wall rupture on both nanostructured substrates (Fig. 5). Moreover, intracellular vesicles and multivesicular bodies, as well as endospore formation, were observed. In *B. subtilis*, the formation of vesicles and multivesicular bodies has been described as a pre-lytic remodelling response that can be triggered by cell envelope damage[39] or by autolytic mechanisms aimed at destroying severely compromised cells or serving as a decoy to protect neighbouring ones.[40] Nanopillared topographies have also been reported to induce both effects in *P. aeruginosa.*[22] Although cell envelope damage was evident in bacteria on both small and large pillars, vesiculation was detected exclusively in the S group, typically coinciding with reduced cytosolic contrast. Given the time- and resource-intensive nature of Cryo-SXT, only a limited number of cells (on the order of tens) could be analysed, precluding statistically robust quantification. Moreover, Cryo-SXT may not detect all vesicles, particularly in cells with intact or dense cytoplasm, due to resolution (~30 nm) and sensitivity limitations. Considering that bacterial viability was comparable on the S and L substrates, we cannot exclude the possibility that vesiculation also occurs on the latter. Notably, no correlation was found between vesiculation and elevated intracellular calcium levels, as a higher proportion of bacteria with increased calcium content was observed on the large-pillar and flat substrates."

Page 25, Lines 422–424: The claim that CDHA is porous after pressing, but not other substrates which are compressed. Please provide evidence, e.g. cross-section SEM images (currently unclear).

We thank the reviewer for this valuable comment. We agree that the statement indicating that flat CDHA is more porous than the nanostructured substrates may appear counterintuitive and merits further clarification. This observation is, however, clearly supported by the nitrogen adsorption data reported in Table S1 (Table S3 in the revised version). The pore sizes of the F, S, and L discs (8.5, 10.7, and 22.1 nm, respectively) are too small to be visualized by cross-sectional SEM.

The difference in porosity arises from the fabrication process: F samples were produced by compacting individual CDHA nanocrystals, generating a network of interconnected nanometric channels that increase the effective specific surface area. In contrast, the S and L samples were obtained by hydrolysis of pre-compacted α-TCP. During this process, α-TCP grains are progressively transformed into interlocked CDHA crystals, yielding a denser structure with fewer interconnected

channels and, consequently, a lower specific surface area. To improve clarity, we have revised the corresponding paragraph in the Discussion section and added the pore size values in Table S3.

Table S3. Specific Surface area, porosity (pore volume) and pore size of the substrates measured by nitrogen adsorption.

	Specific surface area (m^2/g)	Pore volume (cm^3/g)	Pore size (nm)
F	41.548	0.095	8.5
S	1.493	0.003	10.7
L	6.636	0.042	22.1

"(…) The higher reactivity of the F samples is coherent with the smaller crystallite size, higher porosity, and specific surface area, as determined by Nitrogen adsorption (Supplementary Information, Table S3). This is a consequence of the preparation protocol since F samples were fabricated by direct compaction of individual CDHA nanocrystals, generating numerous interconnected nanometric channels that increase the effective surface area. In contrast, S and L samples were produced by hydrolysis of pre-compacted α-TCP, where *in situ* crystal growth results in interlocked CDHA structures with lower interconnected porosity. Additionally, the higher Ca release from L samples is likely attributable to their β-TCP content, which is a more soluble phase than CDHA.[28,42] (…)"

Page 25, Lines 426–428: P in F is low; S, L, and Ctrl have slightly higher P (Line 328). Is this due to PBS treatment?

The Ionic exchange of CDHA with surrounding fluids is mediated by a hydrated surface layer that plays a key role in the exchange process. This mechanism does not follow the rules of congruent dissolution and is highly dependent on the composition of the solution and the exposure time. When immersed in PBS, which lacks calcium, all substrates exhibited calcium release accompanied by phosphate uptake. The latter can be attributed to the high surface charge of certain CDHA crystal faces, which promotes the adsorption of phosphate ions from the medium. A greater extent of ion exchange (both calcium release and phosphate uptake) was observed for the F substrate compared with S and L samples, resulting from its higher specific surface area and porosity, as discussed in the response to the previous question.

We have reorganized the following paragraph in the Discussion section to provide this information more clearly.

Discussion

(…)

"Given the bioactivity of CDHA, ionic interactions were further investigated. Ionic exchange in CDHA is mediated by a hydrated surface layer and does not follow the rules of congruent dissolution. Upon immersion in PBS, which lacks calcium, CDHA released Ca^{2+} while simultaneously uptaking phosphate, indicating concurrent dissolution and sorption processes[41]. The latter can be associated with the positive charge of Ca-rich CDHA crystal faces, which favour phosphate adsorption from the medium. A mild acidification was also observed, as previously described for this material[41]. Ionic exchange was most pronounced in the flat substrate, followed by the large and small topographies. The higher reactivity of the F samples is consistent with their higher porosity and specific surface area, as determined by nitrogen adsorption (Supplementary Information, Table S3)."

Figure 2 shows high Ca2+ for F and L, but no corresponding increase in bactericidal action is reported. This suggests calcium has no effect on bactericidal action—please address this contradiction.

We thank the reviewer for highlighting this point. We agree that higher Ca^{2+} release was observed for the Flat (F) and Large pillar (L) substrates (Fig. 3), yet this was not accompanied by a corresponding increase in bactericidal activity. This observation is central to our study, as it demonstrates that calcium ion exchange alone does not induce bacterial killing under our experimental conditions. Consequently, the mortality observed on the nanostructured substrates must be attributed to the mechanical stress imposed by the nanotopography, rather than to ionic exchange. This distinction is critical, as it separates the chemical and topographical contributions of CDHA surfaces and confirms that the bactericidal efficacy arises specifically from the nanotopography. This point was addressed in the final paragraph of the Discussion in the original manuscript.

"(…) While these results illustrate that ionic exchange between the substrate and surrounding fluids can influence the internal concentration of *B. subtilis*, our findings indicate that this process does not directly impact its mortality.(…) In fact, the substrate exhibiting the highest ionic exchange capacity, the flat substrate (F), did not result in increased mortality rates compared to the control (Fig. 2b), suggesting that ionic exchange alone is not a determining factor in bacterial viability under the conditions tested. This observation is consistent with the known halotolerance of *B. subtilis*,[44,45] which enables adaptation to environments with elevated salinity.[46]"

Calibration with known Ca2+ concentrations is not described. Are the confocal measurements relative or absolute?

We thank the reviewer for this question. The Fluo-4 AM confocal measurements in our study are relative, not absolute, as we did not calibrate the fluorescence signal against known Ca^{2+} concentrations. However, our interest lies in quantifying the proportion of the bacterial population that was stained under each condition, independently of their absolute intracellular Ca^{2+} concentration. This approach allowed us to compare relative differences across conditions while avoiding assumptions about absolute intracellular Ca^{2+} levels.

4.2 XANES Spectroscopy

Figure 6: Please provide a pre-edge image to establish background contrast. The scale and field of view in the L2 edge and Ca map differ—please explain. Also, dark particles in the Ca map appear larger; why? Remove disruptive labels within image and use consistent symbols.

We thank the reviewer for these constructive questions and suggestions. We have updated Figure 6 with the proposed changes, and we believe they significantly improve the clarity and consistency of the data presentation. The following changes have been made:

- A pre-edge image has now been included to establish background contrast.
- In the original version, the image shown at the Ca L_2 edge corresponded to a tomographic projection at 0°, while the Ca map was generated from the stack of images acquired for the XANES spectra, this originated the slight difference in field of view. We have revised the figure so

that all images are now taken from the XANES image stack, ensuring that the scale and field of view are preserved across panels.

- The apparent difference in particle size is likely related to the issue raised in point 2, since the images were originally obtained with different acquisition methods (tomography vs XANES). By how XANES spectra are acquired, the focal plane is recalibrated for each energy step; therefore, slight movements around the focal plane are possible, resulting in a less focused image. In addition, gold fiducial markers absorb strongly due to their high electron density and absorption edges within the same energy range (353 eV, 335 eV), which contributes to the larger dark contrast observed in the Ca maps.
- Disruptive labels have been removed and symbols have been made consistent throughout the figure.

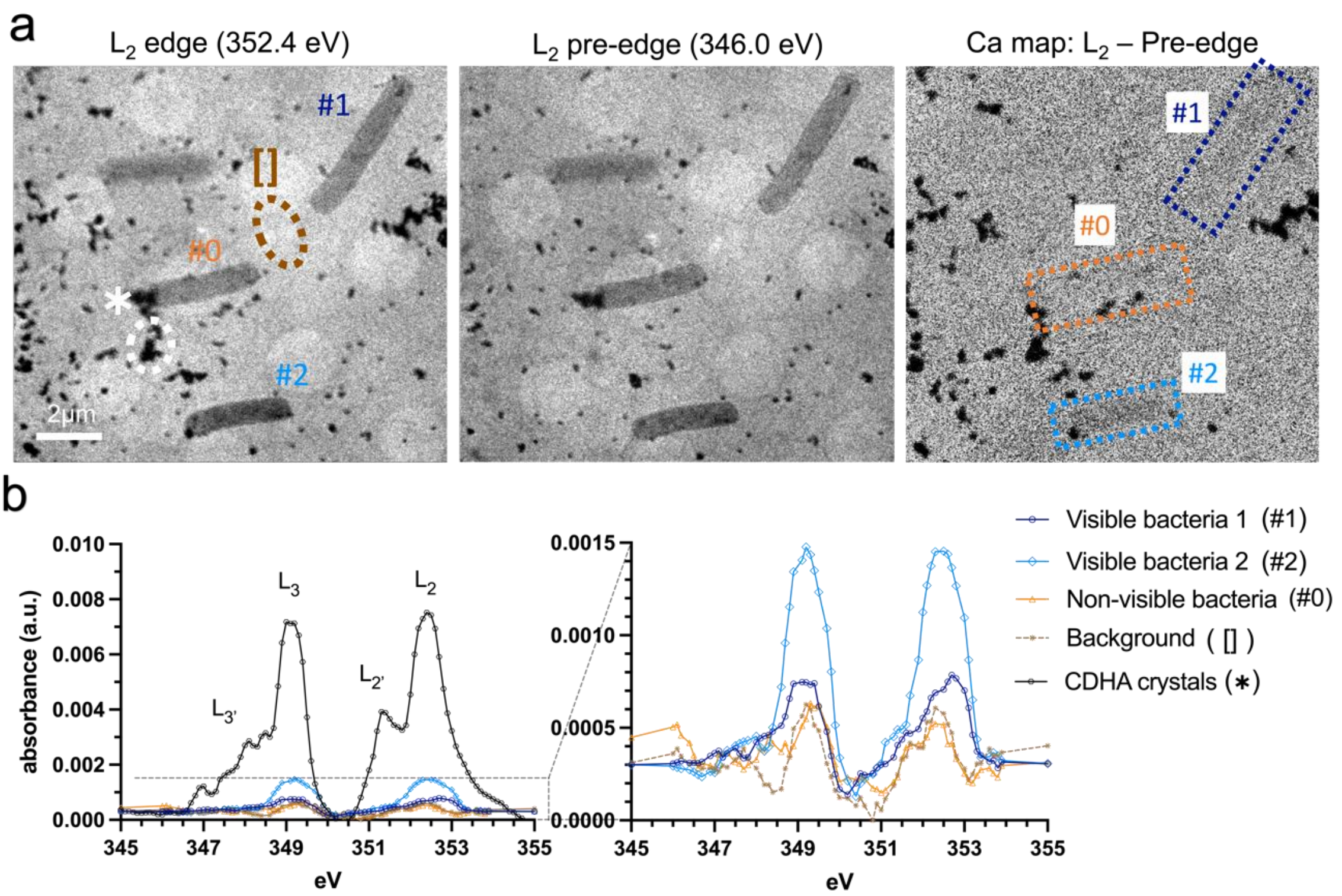

Figure 1. XANES spectromicroscopy at the at the Ca $L_{2,3}$-edges. *A) Normalized projection image of vitrified B. subtilis incubated on flat CDHA, obtained at the Ca L_2 pre-edge (346.0 eV), the L_2-edge (352.4 eV), and the corresponding calcium map obtained by subtracting the pre-edge (346.0 eV) from the edge image (352.4 eV); b) XANES spectra extracted from the different entities highlighted in* ***a,*** *including the background signal, CDHA crystals, two visible bacteria and a bacterium not visible in the Ca map. The spectra reveal distinct Ca $L_{2,3}$ absorption intensities in consonance with the contrast in the Ca map.*

Figure 7:

Line 281: How many bacterial cells were analyzed via XANES? Please provide representative data for multiple cells.

We thank the reviewer for this question. We agree that providing data from multiple cells strengthens the reliability of the results. Accordingly, we have included three spectra per condition,

each acquired from a different XANES image stack. The results show that bacteria incubated on the Large (L) and Flat (F) substrates displayed the characteristic Ca L_2 edge peaks. In the Small (S) condition, only one spectrum exhibited a defined Ca signal, while the other two displayed lower, less distinct peaks, with energy shifts at the L_3 edge that make Ca attribution uncertain. In the control condition, spectra generally showed lower peaks, with one spectrum failing to display the L_2 edge altogether.

XANES spectra for multiple cells are provided in Fig. S3 of the Supplementary Information.

Supplementary Information

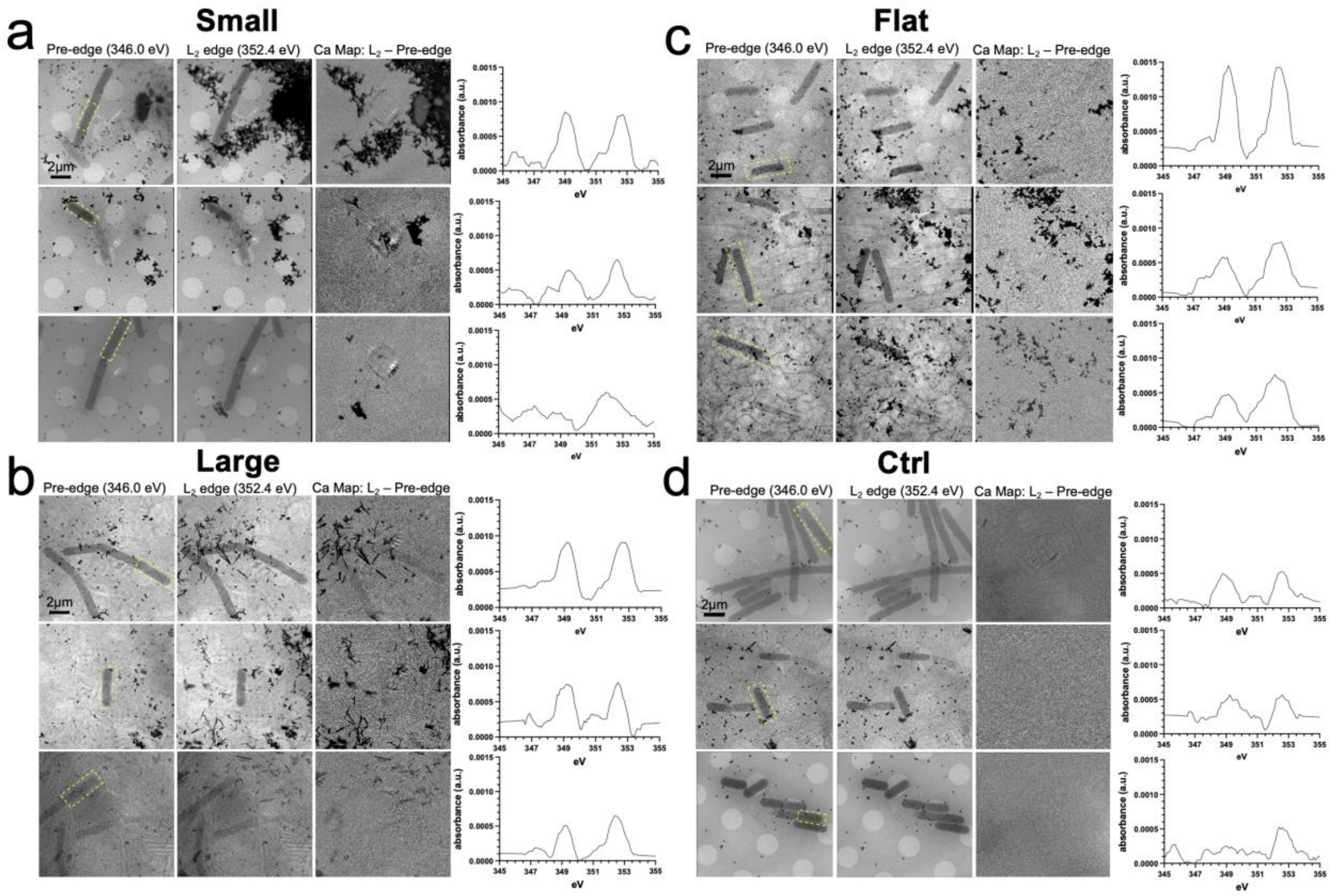

Figure S3. Calcium maps and XANES spectromicroscopy of bacteria incubated on CDHA substrates (S, L, F) or in the culture well (Ctrl). *Representative images and XANES spectra of B. subtilis incubated for 3 h on (a) Small, (b) Large, (c) Flat CDHA substrates, and (d) in the culture well (Ctrl). Normalized projection images of bacteria acquired at the Ca pre-edge (first column), L_2-edge (second column), and Ca maps generated by subtracting the pre-edge from the Ca L_2-edge image (third column). Corresponding XANES spectra at the Ca $L_{2,3}$-edges were extracted from the cytosol of the bacteria outlined with a yellow dashed rectangle in the Ca pre-edge images. For each condition, three spectra were acquired from different cells identified in the Ca maps. The plots illustrate variability among cells and confirm that spectra from Large and Flat conditions exhibit characteristic Ca $L_{2,3}$ edge peaks, while signals from Small and Control conditions are weaker or absent..*

XANES acquisition requires significantly longer exposure times to cover 20 eV in 0.1 eV steps than single-energy tomographic imaging, which limited the number of cells that could be analyzed. Consequently, XANES data are presented as proof of concept for Ca speciation, whereas LAC quantification was used to compare intracellular Ca concentrations across conditions and populations. This correlation between LAC and XANES can be assumed because, in samples with similar thickness, such as the bacterial cells in this case, the LAC value is directly related to the height of the Ca L_2 edge peak. While the energy position of this absorption edge is fixed for Ca, its intensity scales with

concentration. Thus, the modest increases in peak height observed in most XANES spectra (slightly above control values, ~0.0005 a.u.) are consistent with the small differences detected in LAC quantification (Fig. 8 of the manuscript).

The control images at 352.4 eV and 346.0 eV look identical. If there is no Ca, why is there absorption at the L2 edge in the control sample? Control samples should show lower absorbance than samples with Ca. Please explain this inconsistency in Lines 313-315.

We thank the reviewer for this question. We believe this may reflect a misunderstanding of the technique. In the energy range of the Ca $L_{3,2}$ edges, other elements besides Ca also absorb X-rays, which explains why absorption is observed even in the control samples.

The key distinction is that while many elements contribute to baseline absorption in this range, only Ca produces a characteristic edge feature of two peaks in absorbance around the $L_{3,2}$ edges. Thus, increases in absorbance at the $L_{2,3}$ energies reflect Ca content, while other elements of the baseline background remain largely unchanged. Hence, an absence of Ca results in no changes in the absorbance between the image acquired at the pre-edge and the image acquired at an edge.

This is precisely why Ca maps are generated by subtracting the pre-edge image from the edge image: this subtraction effectively removes the contribution of other elements and isolates the signal specific to Ca.

How many cells were analyzed for each treatment group?
As shown in Fig.7 b), the number of cells analyzed were 13 for ctrl, 55 for Small pillars, 44 for Large pillars, and 32 for the Flat surface.

Use consistent y-axis scales across Figures 6 and

We appreciate the reviewer's suggestion. The Y-axis scales in Figures 6 and 7 have been unified.

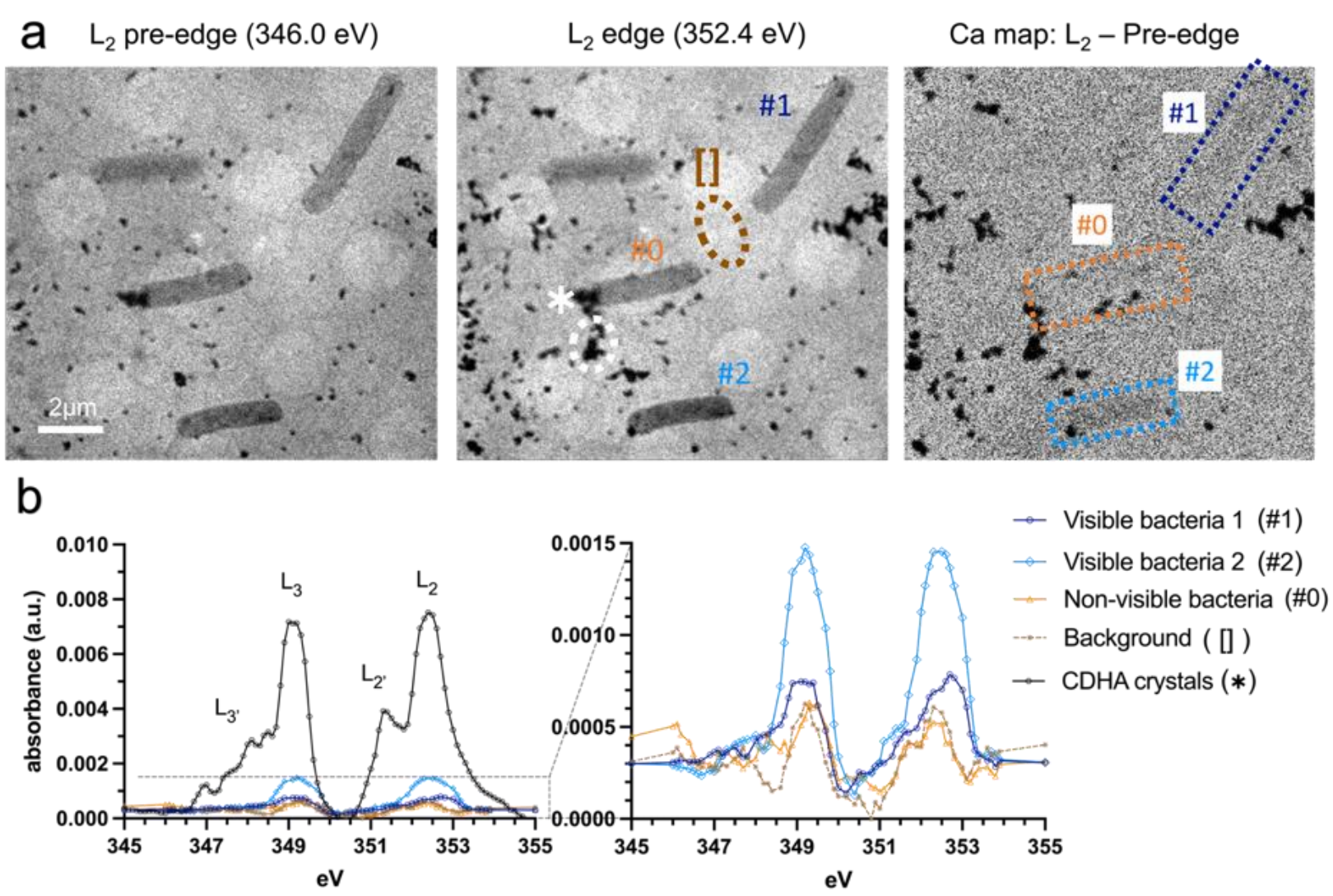

Figure 6

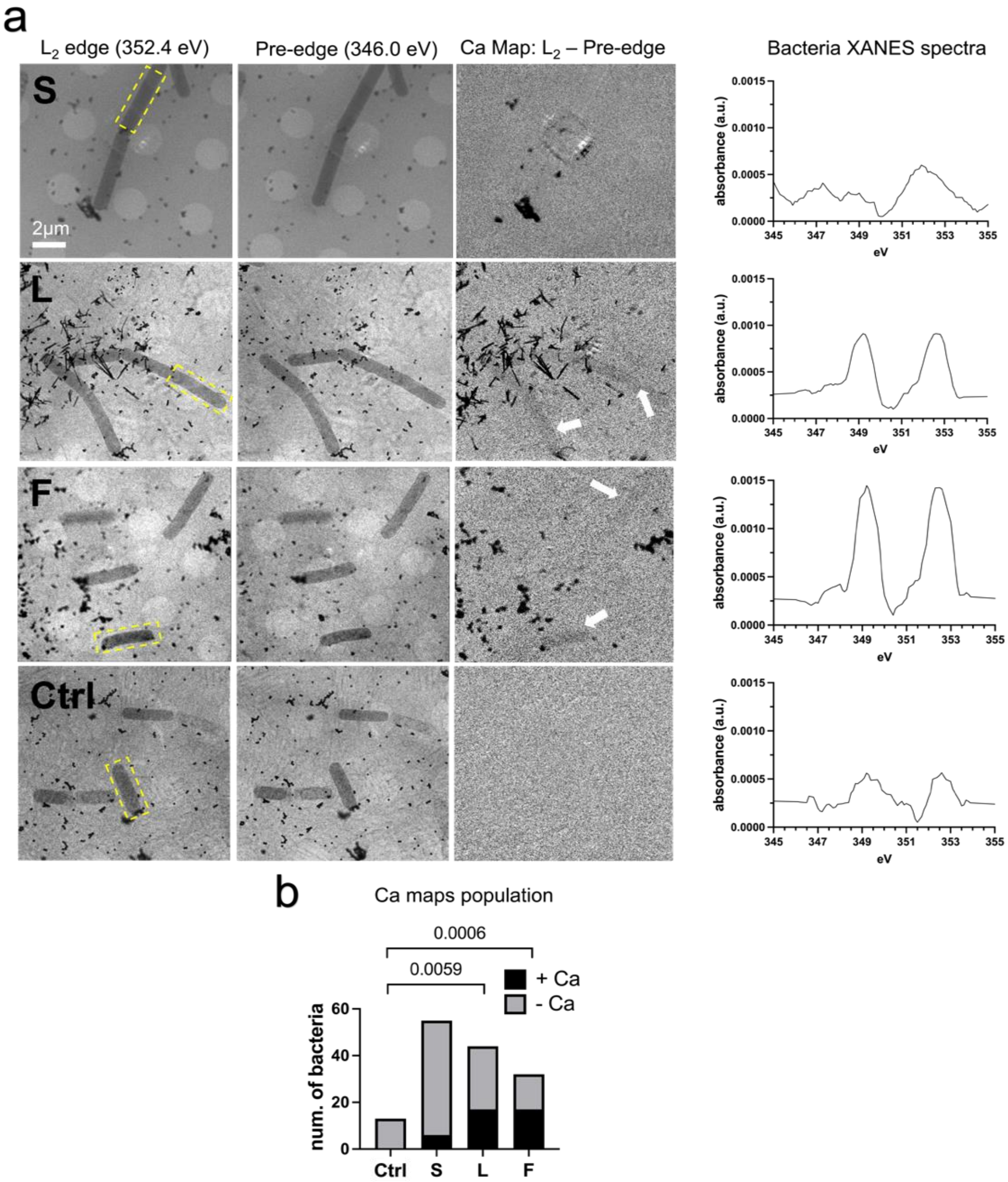

Figure 7

Figure 8a–b: Scales and field of view differ. Please explain.

We thank the reviewer for pointing out this inconsistency. The original image in Fig. 8a was mistakenly taken from the XANES image stack instead of the tomography. Although the XANES image stack corresponded to the Ca L_2 edge, the field of view slightly differed as a consequence of being from a different dataset.

We have updated Figure 8 with the appropriate tomography slice acquired at the Ca L_2 edge, ensuring that scale and field of view are now consistent across panels.

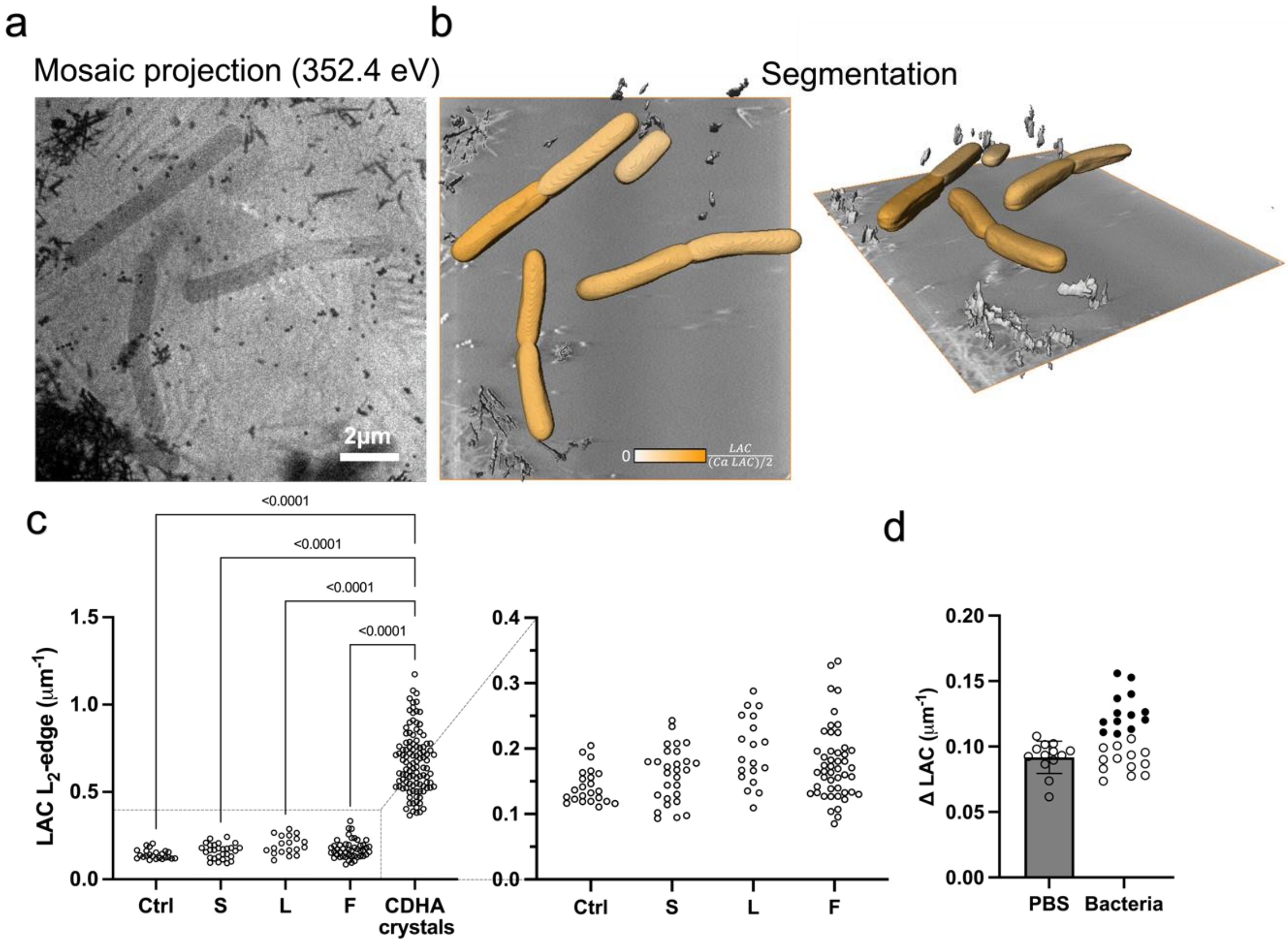

Figure 8. LAC analysis at the L_2-edge between substrates and ΔLACs from the pre-edge for Ca concentration estimation. *A) Cryo-SXT projection of bacteria exposed to large topography; b) Segmented 3D reconstructions of individual bacteria and CDHA crystals from the tomography, shown from top and tilted views. The color scale indicates relative LAC values, ranging from 0 (white) to the maximum value, set as half of the LAC of CDHA crystals (orange). CDHA crystals are shown in grey to differentiate them from bacteria. Segmentation enables the quantification of cytosolic LAC independently from the surrounding material. C) Scatter plot showing LACs of individual bacteria and CDHA crystals, corresponding to all bacteria identified in four independent tomographies per condition. Each data point represents the mean LAC value of a single bacterium, calculated as the average voxel intensity of the segmented volume. Statistical differences were determined by a Kruskal-Wallis test; d) LAC increment (ΔLAC) between the L_3-edge (349 eV) and the pre-edge (346 eV) for vitrified PBS and bacteria incubated on large pillars. The bar indicates the mean and standard deviation of ΔLACs for PBS. The scatter plot displays ΔLACs of individual bacteria. Black dots represent bacteria with ΔLAC significantly higher than PBS, assessed by 1-sample t Test (α=0.01).*

Line 347: Ca concentrations are reported for bacteria detached from the surface. How can this be considered " Ca concentrations of bacteria in contact with the nanotopography"?

We thank the reviewer for this observation and agree that the original wording can be misleading, as it suggests that Ca concentrations were measured directly in attached bacteria. Indeed, the analyses were performed on bacteria that were adhered to the nanotopographies during incubation and subsequently detached by sonication.

To clarify this point, the text before Figure 8 has been revised as follows.

"[…] Taking into account that the calcium concentration of PBS, determined by ICP (Fig. 3), is known (4.53 µM) and that, according to the Beer-Lambert law, there is a linear relationship between LAC values and concentration, it is possible to estimate the intracellular calcium concentration of the bacteria that had been in contact with the nanotopography. The highest measured ΔLAC was 0.156 μm^{-1}, corresponding to a intracellular Ca concentration of 7.68 µM.

4.3 Terminology and Consistency

There is inconsistency in referring to "CaP" and "CDHA" in text and in images. Are these terms interchangeable or distinct in your study? Please clarify throughout the manuscript.

We thank the reviewer for this observation. In our manuscript, the term CaP or CaPs was intended to be used to refer broadly to calcium phosphate biomaterials, whereas CDHA was intended to denote the specific form of calcium phosphate (calcium-deficient hydroxyapatite) employed in this study.

We agree with the reviewer that there were inconsistencies in the usage of these terms, which could cause confusion. We have carefully revised the manuscript to ensure consistent terminology throughout: "CDHA" is now used exclusively when referring to the material studied experimentally in this work, while "CaP" is used only in a general sense when discussing calcium phosphate biomaterials as a broader class.

The following changes have been implemented in the revised manuscript:

1st change: Results – Antibacterial activity

"The antimicrobial activity of the ~~CaP~~ CDHA surfaces against the Gram-positive bacterium *B. subtilis* was evaluated after 3h of incubation by a static adhesion assay, which is typically suited for evaluating contact-killing surfaces.[47] Fig. 2 shows […]"

2nd change: caption Figure 3

"Figure 3. Calcium, phosphate and pH levels in the medium surrounding the different ~~CaP~~ CDHA nanotopographies, in the presence or absence of *B. subtilis*. A) Ca and P concentrations in the supernatant after 3h incubation in PBS in absence (grey) or in presence of *B. subtilis* (orange) assessed by ICP (n=6, two independent experiments). B) pH registered in the supernatant in the same conditions (n=3). The controls were PBS (Ctrl) and PBS with bacteria (Ctrl+BS), without ~~CaP~~ CDHA substrates. […]"

3rd change: Cryo-soft X-ray Tomography

"Bacteria were imaged by cryo-soft X-ray tomography (Cryo-SXT). Tomograms of vitrified cells were acquired at 520 eV where carbon-rich elements like proteins, lipids, and nucleic acids exhibit a strong absorption contrast.[48] Imaged bacteria were detached from the nanostructured ~~CaP~~ CDHA surfaces, the flat ~~CaP~~ CDHA surface or the culture well after 3 hours of incubation."

5. Data Interpretation and Conclusions

The authors claim the substrate is suitable for bone integration. However, no experiments on osteointegration or relevant cell lines are presented. Please provide supporting data or temper the claims accordingly.

We thank the reviewer for this comment. No osteointegration experiments or assays with eukaryotic cell lines were performed in the present study. However, our group has demonstrated that tuning the nanostructure of CDHA was an effective method to control the response of eucaryotic cells involved in the bone regeneration process. In particular, nanoneedles promoted a more favorable osteogenic environment, influencing not only immune cells (*Sadowska et al., Biomaterials 182,381-32, 2018*), osteoclasts (*Ciapetti et al., Acta Biomater. 50, 102-13, 2017; Diez-Escudero et al. Tissue Eng Part C Methods. 23, 118-24, 2017*), mesenchymal stem cells (*Sadowska et al. Acta Biomater. 76,319-32, 2018, Sadowska et al. Tissue Eng Part A. 23, 1297-399, 2017*) and endothelial cells (*Sadowska et al., Adv Healthc Mater. 8,1801138, 2019*) *in vitro* but also supporting bone formation *in vivo (Barba et al. ACS Appl Mater Interfaces. 11,8818-30, 2019; Barba et al. Acta Biomater. 79,135-47,2018; Raymond et al. Acta Biomater. 135, 671–88,2021).*

We have included this information in the Discussion section, reorganizing some of the paragraphs as shown below.

Discussion

(…)

"The situation becomes more complex when these nanotopographies are developed in bioactive materials, such as calcium phosphates, which are intended to simultaneously promote bone regeneration and exert local antimicrobial action. The ease of tuning the shape and size of CDHA crystals by reaction parameters has been widely exploited for the promotion of osteogenesis. In our previous work, we showed that high-aspect-ratio, needle-like CDHA nanocrystals provide a more favorable osteogenic environment, influencing not only immune cells[24], osteoclasts [25,26], mesenchymal stem cells [27,28] and endothelial cells[29] *in vitro* but also promoting bone formation *in vivo.* [30-32] In this case, ionic exchange with the surrounding environment introduces additional variables that must be considered alongside mechanical stimuli.

(…)

High-aspect ratio topographies of CDHA nanopillars were synthesized by hydrolysing α-TCP under controlled temperature conditions. ~~The ease of tuning the shape and size of CDHA crystals by reaction parameters has been widely exploited for the promotion of osteogenesis.[29,31]~~ Increasing the hydrolysis temperature from 37 to 100°C accelerated the hydrolysis reaction,[36] yielding taller nanopillars with greater interpillar spacing. However, crystal thickness remained unaffected (Fig. 1a)."

The effects of Ca and P are not clearly contributed for bactericidal action as per results. However, authors claim synergistic effects of surface chemistry and nanotopography on bactericidal action. This statement is not justified with the data from the manuscript.

We thank the reviewer for this comment and the opportunity to clarify this issue. We did not intend to claim synergistic effects of surface chemistry and nanotopography, as the results of our study pointed otherwise, as we stated for instance in the Abstract: "XANES cryo-spectromicroscopy and linear absorption coefficient (LAC) analysis revealed a subpopulation with elevated intracellular calcium concentrations, which is correlated with calcium release from the substrate and confirmed by confocal microscopy. Even so, it had no effect on viability, consistent with the halotolerant nature of *B. subtilis*." and also in the Discussion section: "While these results illustrate that ionic exchange

between the substrate and surrounding fluids can influence the internal concentration of *B. subtilis*, our findings indicate that this process does not directly impact its mortality.(…) In fact, the substrate exhibiting the highest ionic exchange capacity, the flat substrate (F), did not result in increased mortality rates compared to the control (Fig. 2b), suggesting that ionic exchange alone is not a determining factor in bacterial viability under the conditions tested. This observation is consistent with the known halotolerance of *B. subtilis*,[43,44] which enables adaptation to environments with elevated salinity.[45]". However, we agree that the last sentence of the Abstract can be misleading. In the Abstract we originally stated: "This study opens new strategies for exploring the complex cross- or synergistic effects of surface chemistry and nanotopography on bacterial response." We have revised the last sentence in the Abstract to avoid implying a synergistic effect not supported by the data.

Abstract

(…)

"XANES cryo-spectromicroscopy and linear absorption coefficient (LAC) analysis revealed a subpopulation with elevated intracellular calcium concentrations, which is correlated with calcium release from the substrate and confirmed by confocal microscopy. Even so, it had no effect on viability, consistent with the halotolerant nature of *B. subtilis*. Moreover, the intracellular calcium concentration is estimated, suggesting active calcium uptake up to micromolar levels. This study opens new strategies for ~~exploring the complex cross- or synergistic effects~~ disentangling the role of surface chemistry and nanotopography on bacterial response."

6. Methodology

Please refer to Section 3 above for details. In its current form, the fluorescence microscopy and SEM sample preparation do not meet field standards and must be repeated using appropriate protocols.

We thank the reviewer for this remark. As suggested, we have repeated both the fluorescence microscopy and SEM experiments using the appropriate standard protocols. The revised results are now included in the updated figures, and the corresponding procedures have been described in detail in the Materials and methods section.

7. Reproducibility

The manuscript generally provides sufficient detail, except for the sonication procedure, which lacks clarity on immersion and power settings. Please expand this section and consider adding a schematic to aid reproducibility. Ideally provide a schematic of the sample preparation in a single figure, so that workflow is clear.

We thank the reviewer for this helpful suggestion. As detailed in our response to comment 3.3, we have expanded the description of the sonication procedure in the Materials and methods section, including immersion conditions and power settings. In addition, we prepared a schematic summarizing the sonication workflow and sample preparation steps, which is included as Figure S5 of the Supplementary Information to aid reproducibility. Please refer to the response to question 3.3 of this reviewer for the detailed description.

Major Revision

Before publication, the authors must:
Repeat key experiments (Live/Dead staining, SEM imaging) using correct sample preparation methodologies. Done
Provide quantitative viability data (CFU or alternative) to support bactericidal claims. Done
Address concerns about sample preparation and discuss limitations of detached cells in Cryo-SXT. Done
Clarify inconsistencies in XANES data interpretation. Done
Provide evidence or a more cautious interpretation of bone integration claims. Done

I suggest the authors can address these points with appropriate data and discussion, the manuscript would significantly improve in rigor and reliability, potentially making it suitable for publication.

Reviewer #3 (Remarks to the Author):

This manuscript presents a study of the interaction between Bacillus subtilis and nanostructured calcium phosphate (CDHA) substrates, using synchrotron cryo-soft X-ray microscopy and XANES spectroscopy to explore both mechanical and chemical effects at the subcellular level. Ca L-edge XANES spectromicroscopy is used to resolve intracellular calcium accumulation in B. subtilis exposed to nanostructured CDHA surfaces. The authors demonstrate that calcium uptake is substrate-dependent, with higher internalisation observed on flat and large-pillar CDHA surfaces. The Ca maps extracted from the XANES spectra distinguish between calcium-rich and calcium-poor bacteria populations, and the XANES spectra suggest the presence of amorphous or soluble calcium species rather than crystalline deposits. Whilst the work has relevance in biomaterials and microbiology the article focusses on the application on the cryo-SXT technique and the information that this, in combination with Ca XANES, can provide in terms of visualising the morphology of the bacteria cell and locating and quantifying the Ca uptake. In this aspect some lovely data is presented, the reconstructions and segmentations are well considered and the methodology is well described. The significance and impact of the results in themselves is however less clear, beyond how the cryo-SXT method can be used, for example how do these results inform the design of nanostructured antibacterial surfaces.
Minor revisions and discussion points are suggested.
Page2 line 12 "Nanotropographies" should be "Nanotopographies"
Page 4 line 23: Cryo-SXR should be Cryo-SXT

We thank the reviewer for these observations. We have revised the manuscript to correct these typographical errors and have thoroughly checked the entire text to ensure consistency and eliminate other potential mistakes.

Page 5 paragraph 1, fig 1: A representative plot of the Reitveld fit, and difference plot, should be presented, along with the refinement R-factor or goodness of fit. The CaP phase have multiple overlapping peaks therefore the quality of the fitting is important when considering the phase fractions. Errors should eb given on the phase fractions presented in Table 1. The authors state that the sharper peaks indicate increased crystallinity – have the peak widths been calculated?

The peak widths are also affected by crystallite size, so it would be expected to have broader peak for the S morphologies, how then is extent of crystallinity determined?

We thank the reviewer for this comment and agree that these points are very relevant for the analysis of the XRD data and the interpretation of phase quantification and crystallinity. To address this, we repeated the XRD analysis, examining three discs per condition. For Small, Large, and Flat topographies the XRD results were analyzed by Rietveld refinement and the crystallite size was estimated from the broadening of the (211) peak using Scherrer's equation. New versions of Figure 1g and Table 1 have been added to update the results obtained with the technical replicates. Moreover, representative plots of the Reitveld fit, and difference plot are presented in Figure S1 and information on the quality of the fitting is provided in Table S1 of the Supplementary Information.

The new results were consistent with our previous findings, which are now reinforced by this more detailed analysis. The Materials and Methods and Results sections have been updated.

Results

"X-ray diffraction (XRD) confirmed CDHA as the main phase in all samples (Fig. 1 g). Phase composition analysis by Rietveld (Table 1) confirmed that hydrolysis at 37 °C (small pillars and flat samples) resulted in CDHA with minor traces of unreacted α-TCP and β-TCP, the latter likely stabilized during the quenching process. The hydrolysis in boiling water (large pillars) resulted in a higher proportion of β-TCP, with similar levels of unreacted α-TCP. As expected, the crystallite size was larger for the L than for the S substrates, while F had the smallest crystalline domains. Complete data of the XRD and crystallite size analysis is displayed in Supplementary Information Table S1 and Figure S1."

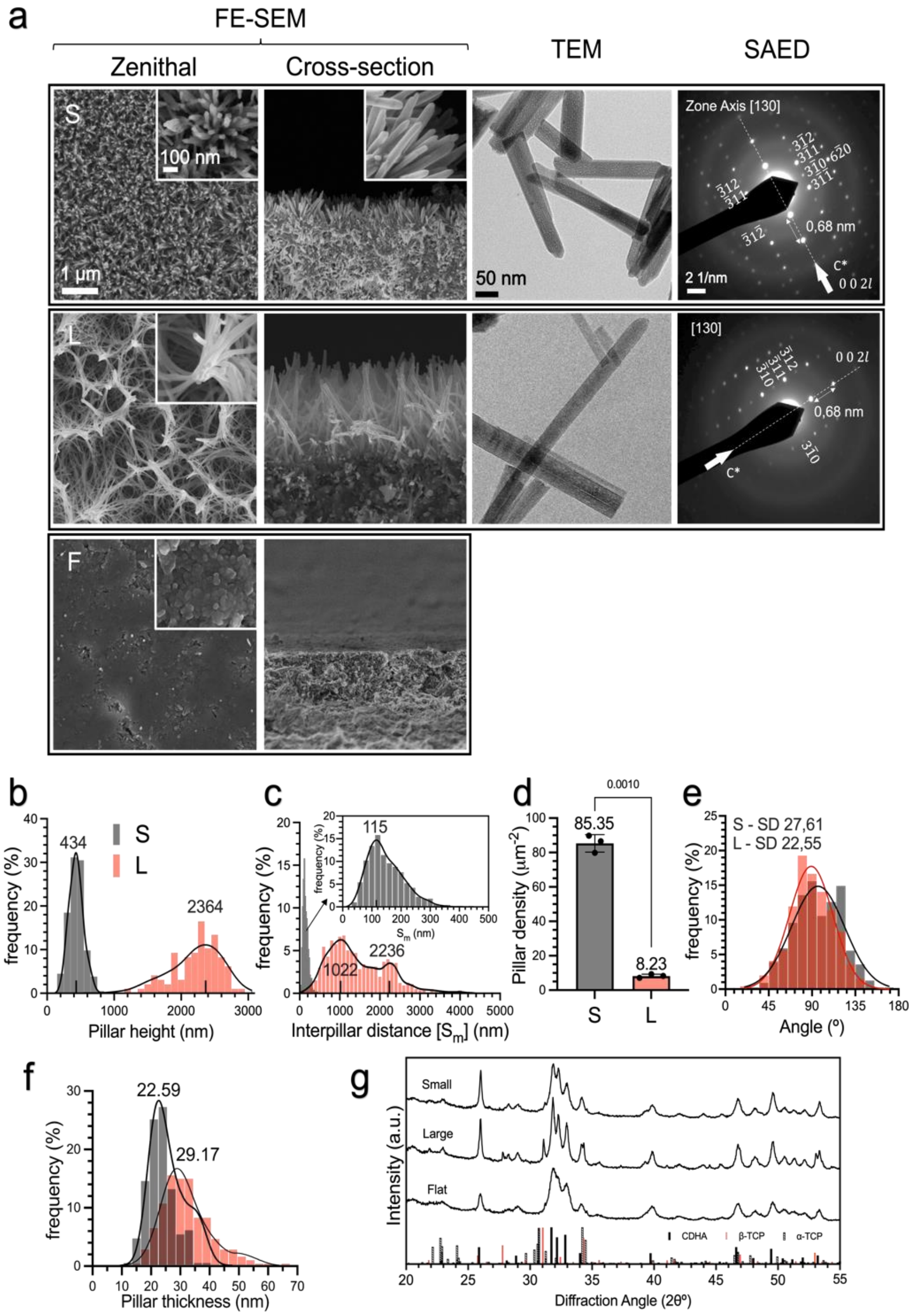

Figure 2. Physico-chemical characterization of the calcium phosphate substrates with different nanotopographies: small pillars (S), large pillars (L) and flat (F)*; a) First two columns: FE-SEM images of the surface and the cross-section of the discs, respectively; Third and fourth columns: TEM images and SAED pattern of a single pillar, respectively; b) pillar height; c) interpillar distance; d) pillar density; e) pillar orientation and f) pillar diameter, all of them quantified from the FE-SEM images. Numbers written on top of the histograms refer to the peak value. Values given for pillar density are mean ± standard deviation and differences were determined by a two-sided Welch's t-test; g) XRD of the different substrates. The bottom bars indicate the pattern of each phase according to JCPDS cards.*

Table 1. *Topographical parameters, together with phase quantification and crystallite size obtained from XRD of the different crystalline phases present on the disks. For surface nanoarchitecture, pillar density is given as the mean value ± standard deviation; all the other parameters correspond to the peak or peaks of the distribution. Average phase composition and estimated standard deviation (ESD) is given in wt.%, and crystallite size is estimated by the Scherrer formula (Eq. 1) using the (211) peak.*

		Small pillars (S)	Large pillars (L)	Flat (F)
**Surface nanoarchitecture**				
	**Pillar density [μm^{-2}]**	85.35 ± 5.08	8.23 ± 1.04	
	**Pillar interspacing [nm]**	115	1022 and 2236	
	**Pillar height [nm]**	434	2364	
	**Pillar thickness [nm]**	29.6	22.2	
**Phase composition (XRD)**				
**CDHA**	Phase ± ESD (wt%)	94.1 ± 0.4	81.8 ± 0.5	95.4 ± 0.4
**β-TCP**	Phase ± ESD (wt%)	3.7 ± 0.4	14.8 ± 0.4	2.5 ± 0.3
**α-TCP**	Phase ± ESD (wt%)	2.2 ± 0.3	3.5 ± 0.3	2.1 ± 0.4
	**χ^2**	1.2906	1.6017	1.2665
	**Crystallite size (nm)**	36.5 ± 1.7	48.6 ± 2.4	25.7 ± 1.7

Discussion

"[…] XRD analysis confirmed that hydrolysis at 37 °C (S and F samples) produced mostly CDHA (>94%), with minor unreacted α-TCP and traces of β-TCP, which is stabilized during the quenching process of α-TCP[36]. Conversely, hydrolysis at 100 °C yielded 14.8% of β-TCP), consistent with previous findings.[31,37]"

Supplementary Information

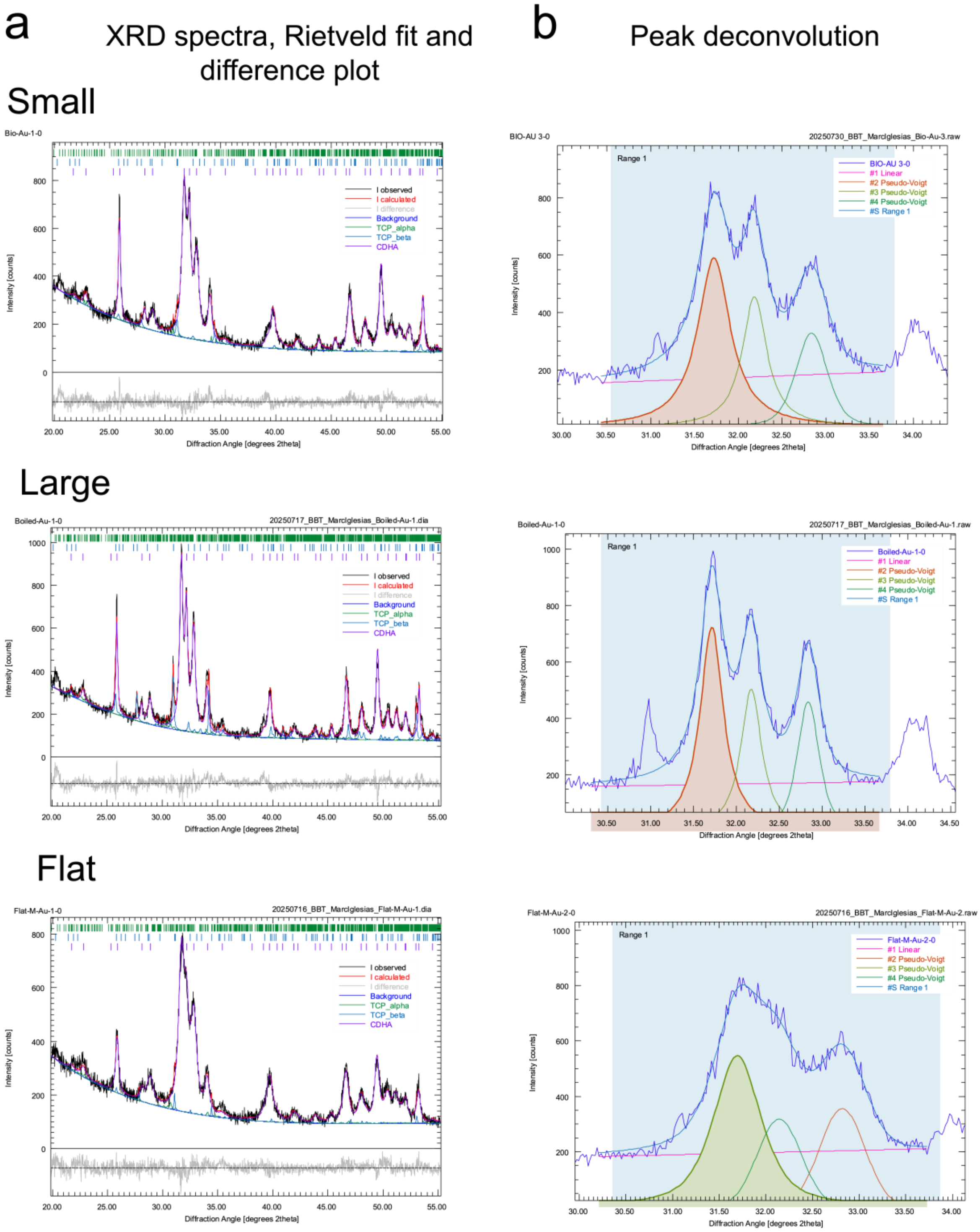

Figure S1. Representative plots of the Rietveld fit, difference plot and peak deconvolution. The shadowed peak was used for the estimation of crystallite size.

Table S2. Phase quantification and crystallite size of the Small, Large, and Flat topographies. *Reported parameters include phase wt.%, estimated standard deviation (ESD), Rietveld fitting quality indicators: weighted profile R-factor (R_{wp}) and expected R-factor (R_{exp}), from which χ^2 and the goodness of fit (GOF) are*

derived. Crystallite size was evaluated from the broadening of the (211) peak using the Scherrer equation. All data were above the limit of quantification (LOQ > 4·ESD) and the limit of detection (LOD > 2·ESD).

		Phase quantification		Rietveldt fitting				Crystallite size
**Topography**	Phase	Phase (wt. %)	ESD (%)	Rwp	Rexp	χ^2	GoF	X_{hkl} (nm)
**Small**	alpha	2.2	0.3	6.820	6.003	1.291	1.136	36.5 ± 1.7
	β-TCP	3.7	0.4					
	**CDHA**	94.1	0.4					
**Large**	alpha	3.5	0.3	7.313	5.783	1.602	1.265	48.6 ± 2.4
	beta	14.8	0.4					
	**CDHA**	81.8	0.5					
**Flat**	alpha	2.1	0.4	6.640	5.900	1.266	1.125	25.7 ± 1.7
	beta	2.5	0.3					
	**CDHA**	95.4	0.4					

Materials and methods.

"Phase composition and crystallite size analysis

The crystallographic phases were identified by X-ray diffraction (XRD) from three replicates analyzed per condition. ~~using a~~ The diffractometer was equipped with a Cu Kα X-ray tube and a primary monochromator. It operated at 40 kV and 40 mA, with data collected in 0.02° steps over 10–60° and a counting time of 2 s per step. Phase quantification was conducted via Rietveld refinement using the Profex software (Profex 4.3.5)[51]. Crystalline models for CDHA, α-TCP, and β-TCP were retrieved from the International Centre for Diffraction Data (ICDD), ICDD PDF 01-086-1201, ICDD PDF 00-029-0359 and ICDD PDF 00-003-0681, respectively. Crystallite size (X_{hkl}) was estimated from the (211) peak of HA using the Scherrer's equation (Eq. 1):

$$X_{hkl} = K * \frac{\lambda}{\beta_{1/2} * \cos(\theta)} \quad \text{Eq.1}$$

Where λ is the wavelength of monochromatic X-ray beam (0.15406 nm for Cu Kα radiation), $\beta_{1/2}$ is the full width at half maximum for the peak under study (rad), θ is the diffraction angle (°), and K is a constant varying with crystal habit and here set to the typical value of 0.89. Peak profiles were deconvoluted using a pseudo-Voigt function (Fig. S1)."

Page 13: sample preparation for cryo-SXT – all bacteria were detached from substrates after 3 h incubation, could this detachment (and any bacteria damage) have occurred during transfer onto the substrate for vitrification eg. During the sonication?

We thank the reviewer for raising this important concern, which was also raised by Reviewer #2 (question 3.3).

We agree that it is reasonable to consider that sonication could, in principle, compromise bacterial integrity and potentially induce artifacts. In this regard, the additional assays included in this revision, comprising: i) metabolic activity measurements (Bactiter-Glo) performed on attached bacteria, and ii) CFU counts of bacteria detached following the sonication protocol used for Cryo-SXT sample preparation, provide highly relevant information (Fig. 2). The viability trends observed in both assays were consistent with each other, as well as with the live/dead staining results obtained for bacteria

adhered to the substrates also shown in Fig. 2. Together, these findings confirm that the sonication procedure did not compromise bacterial viability or alter the integrity of the cells.

To further address this point, we performed an additional experiment to assess whether the sonication protocol affected bacterial viability. Live/dead confocal imaging (without glutaraldehyde fixation) was conducted on bacteria detached from the topographies using the same sonication procedure. Quantitative analysis showed no significant differences in mortality between bacteria adhered to the substrates or detached by sonication, across all tested topographies. These results confirm that the sonication step did not compromise bacterial integrity, supporting the validity of the Cryo-SXT observations.

We have included this information in the Discussion section and in Fig. S4 of the Supplementary Information:

"Discussion

(…)

Notably, FE-SEM images evidenced the presence of severely damaged bacteria on the nanostructured surfaces, and Synchrotron Cryo-SXT provided very relevant information in this respect, as it allows imaging whole bacteria in the hydrated state with minimal pre-processing beyond sonication and vitrification. Because images are acquired in transmission mode, detachment of bacteria from the substrate is required. While this represents a methodological limitation for biomaterial studies, the strong agreement between metabolic and Live/Dead assays on attached bacteria and the CFU counts of bacteria detached by sonication (Fig. 2) confirms that the sample preparation does not affect bacterial viability or mortality, ensuring the reliability of the observed effects. Furthermore, as shown in Fig. S4, Live/Dead confocal imaging of bacteria detached using the same protocol revealed no significant differences in mortality between adhered and detached bacteria across all tested topographies."

Supplementary Information

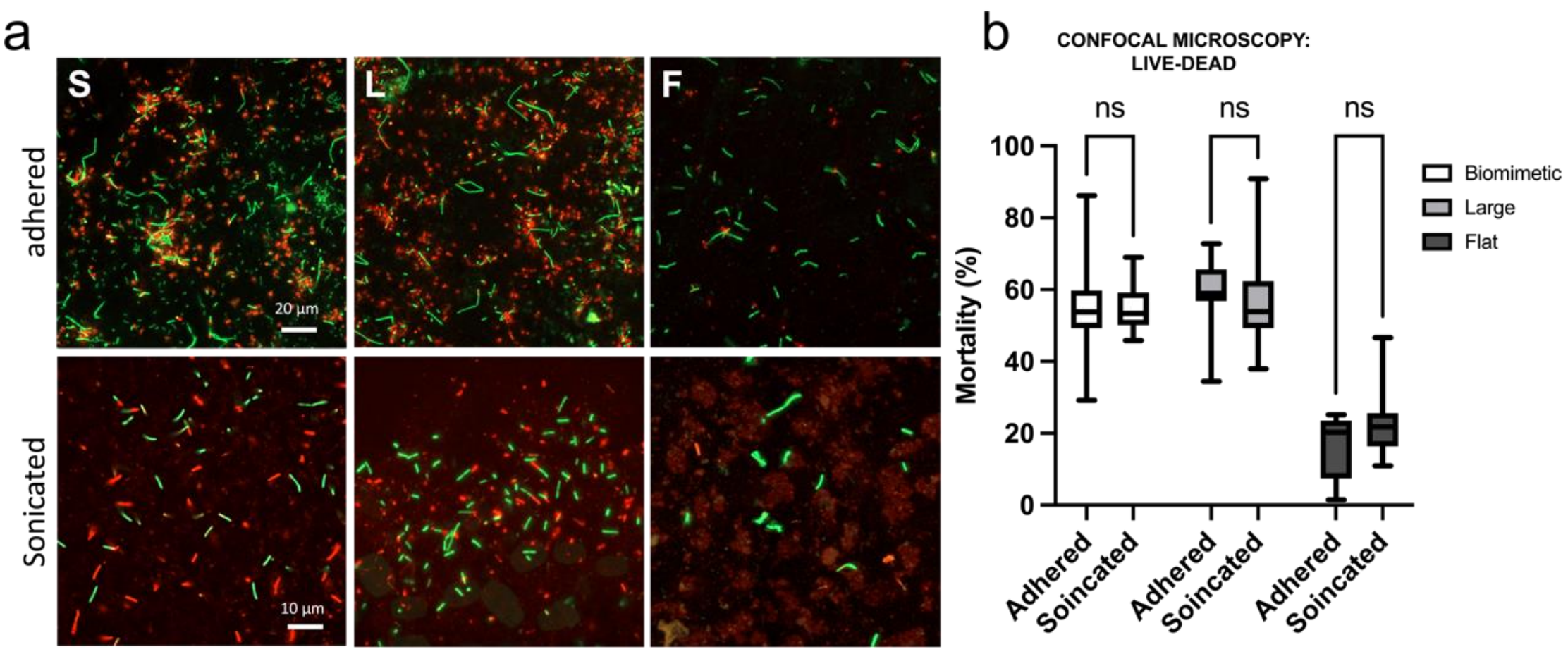

Figure S2. Assessment of the impact of the sonication protocol (30 s in 40 µL PBS) on bacterial viability and mortality. *To assess whether sonication affected bacteria mortality, we compared Live/Dead staining of bacteria adhered to the topographies with those detached following the sonication protocol. Live/Dead staining of detached bacteria was performed as follows: each PBS suspension of sonicated bacteria was placed on a confocal cover lid, and 0.5 µL of Live/Dead reagent was added directly to each suspension. Images were acquired after 7 min incubation and up to a maximum of 15 min, in*

accordance with the manufacturer's recommended incubation window. (a) Confocal images of adhered (top row) and sonicated (bottom row) bacteria from Small (S), Large (L), and Flat (F) topographies. Notably, the red background of sonicated bacteria corresponds to CDHA crystals. (b) Boxplots of mortality of sonicated and adhered bacteria after 3 h (n=12). Box plots indicate median (middle line), 25th, 75th percentile (box) and 5th and 95th percentile (whiskers). No outliers were observed.

How was the 3h incubation time chosen?

The 3 h timepoint was selected for a combination of scientific and practical reasons: Our aim was to assess the efficacy of the nanotopographies at short incubation times and capture early events in bacterial viability and ultrastructural changes. Additionally, the Cryo-SXT experiments were performed at the BL09 Mistral beamline at the ALBA Synchrotron, where each run allows for four sample slots. As we studied four different conditions in this work, the available capacity did not permit inclusion of multiple timepoints.

Ca – XANES:

How was the effect of beam damage considered and measured for the Ca XANES data collection, method section states no beam damage was observed?

We agree with the reviewer that it is important to address the possibility of beam damage. Cryogenic conditions help minimize but not fully prevent radiation-induced artifacts. Due to the tight beam time available, no optimization experiments were performed to reduce beam damage. However, only a few image stacks displayed radiation damage (e.g. local loss of contrast, sample deformation, or the appearance of bubble-like voids in the reconstructed volumes) and were excluded from the analysis. Moreover, calcium analysis is comparatively less susceptible to beam-induced damage than elements whose oxidation state can be altered by radiation, as calcium exists exclusively in the Ca^{2+} oxidation state.

In Materials and methods, we added a comment regarding beam damage.

"XANES spectra

Bacteria and CDHA crystals were chosen as regions of interest (ROIs) in the calcium maps. ROIs were manually selected within the scanned area of the aligned 2D image stacks. Absorbance spectra were obtained by plotting the average ROI signal on each image across all energy steps. Due to the tight beam time available, no optimization experiments were performed to reduce beam damage. However, only few image stacks displayed radiation damage and were excluded from the analysis. FIJI ImageJ v2.9.0/1.53t was used for data extraction, and spectra were processed and plotted using a custom Python 3 script, provided as Supplementary Information."

For the identification of the spectra, plots of standard Hap Ca XANES and amorphous CaP should be provided as reference data for comparison. Ca spectra in a bacterial environment may be different to standards, are there any literature comparisons for this?

We thank the reviewer for raising this point. We have included the XANES spectra of hydroxyapatite and of soluble calcium in solution (50 mM $CaCl_2$ solution) taken from the literature in Figure S2 of the Supplementary Information, as reference data for comparison.

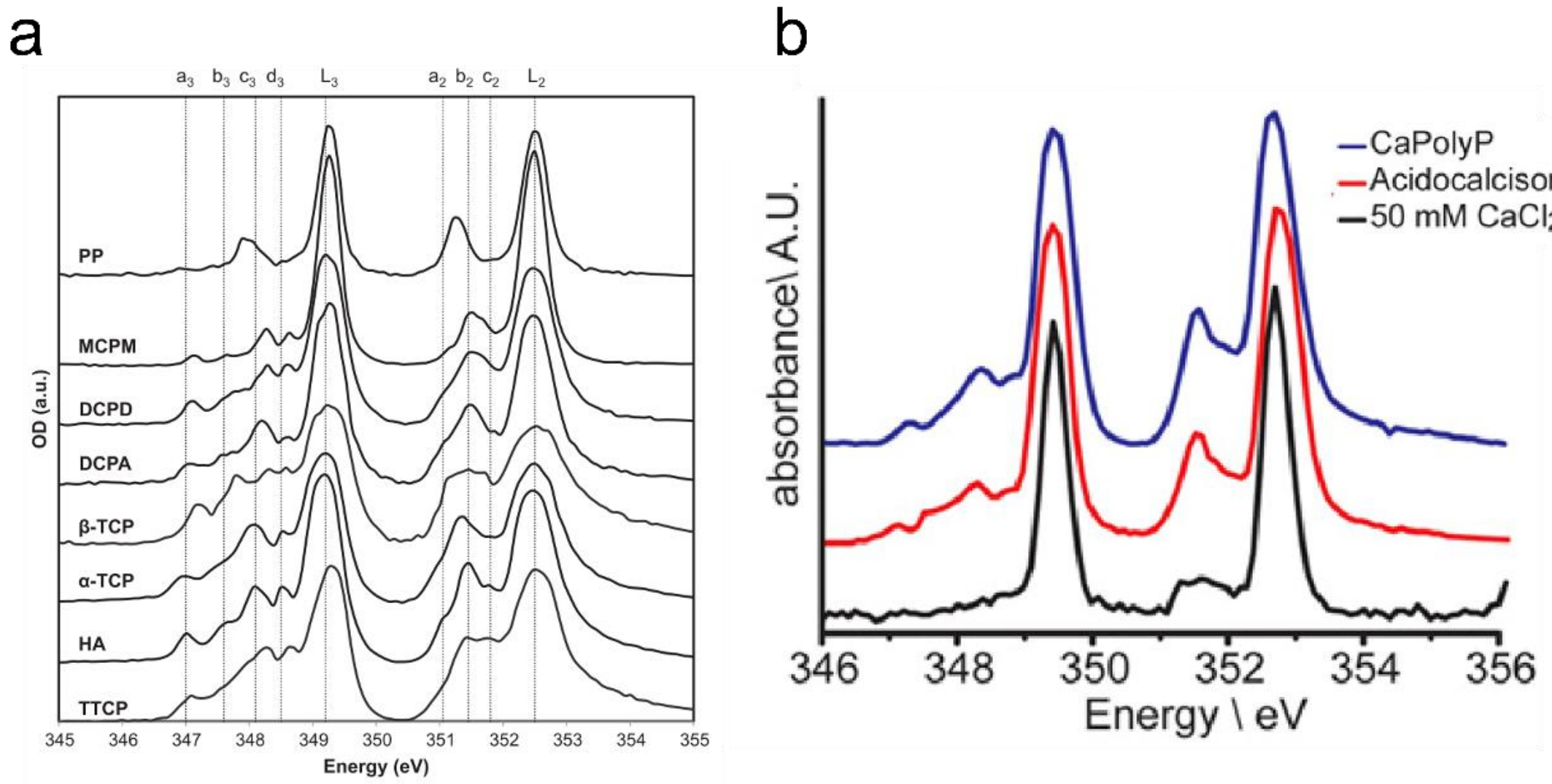

***Figure S2**. a) XANES spectra of the Ca $L_{2,3}$-edges of several Ca-phosphate reference compounds, including hydroxyapatite (HA), polyphosphates (PP), monocalcium phosphate monohydrate (MCPM), dicalcium phosphate dihydrate (DCPD), dicalcium phosphate anhydrous (DCPA), beta tricalcium phosphate (β-TCP), alpha tricalcium phosphate (α-TCP) and tetracalcium phosphate (TTCP). Vertical dotted lines correspond to the peak energies of HA. From J. Cosmidis, et a. Acta Biomater. 12, 260–269 (2015). b) Ca $L_{2,3}$ XANES spectra of calcium polyphosphates, acidocalciosimes of C. reinhardtii and a $CaCl_2$ 50 mM solution in water. From A. Gal, et al., Proc. Natl. Acad. Sci. U. S. A. 115, 11000–11005 (2018).*

In our study, Ca XANES spectra were acquired from the crystals detached from the substrates, which were identified as calcium-deficient hydroxyapatite by XRD (Fig. 1g, Table 1). As shown in Fig. 6b, these spectra closely match those of hydroxyapatite (HA) (Fig. S2 a), displaying the characteristic L_2 and L_3 absorption peaks as well as the distinctive pre-peaks. A mention to the corresponding reference patterns has been included in the text of the manuscript.

"Calcium distribution and XANES spectra

(…)

"The spectra were generated by averaging the absorbance values across the selected areas spanning the Ca $L_{2,3}$-edge. As expected, the CDHA spectrum showed the characteristic doublet structure of hydroxyapatite, including the L_2' and L_3' pre-peaks, consistent with previous reports[18], as shown in Fig.S2a of the Supplementary Information."

Regarding the XANES spectra of the cytosol of the Ca-rich bacteria, although the signal intensity was relatively low, close to the detection limit due to the micromolar intracellular concentrations, the Ca L_2 and L_3 peaks were clearly discernible, and no defined $L'_{2,3}$ pre-peaks were observed. The obtained spectra were consistent with calcium ions in solution, as reported for $CaCl_2$ solutions (*Gal, et al., PNAS 115, 11000–11005 (2018)*, Fig. S2b)) and seawater (*Kahil et al., PNAS 117, 30957–30965 (2020)*), exhibiting a low or absent L_2' peak. This, together with the observation that Ca maps revealed a homogeneous distribution throughout the bacterial cytosol support the interpretation that Ca was in soluble form. Any solid precipitate would produce a stronger and spatially heterogeneous signal in the bacteria. Moreover, as shown in Fig. 2a), XANES spectra of solid CaP

display a distinct L_2' peak which can reach up to half the height of the L_2 edge, which is not observed in our spectra. This information has been incorporated in the manuscript as follows.

"The XANES spectra of bacteria cytosol were compared across conditions. Similar spectra were obtained for ~~the~~ Ca-rich bacteria incubated on L and F CDHA substrates, showing well-defined Ca L_2 and L_3 peaks, while no defined $L'_{2,3}$ pre-peaks were observed ~~remained undefined~~. ~~This~~ The spectra profile is consistent with ~~amorphous hydrated Ca or soluble calcium forms~~ calcium ions in solution, as previously reported ~~in the literature.~~ ~~[14,18]~~ for instance in $CaCl_2$ solutions[19] (Fig. S2b) and in seawater,[14] both exhibiting a low or absent L_2' peak. This, together with the observation that Ca maps revealed a homogeneous distribution throughout the bacterial cytosol, support the interpretation that Ca was in soluble form. In contrast, bacteria incubated in the empty culture wells and most of those on small pillars, exhibited low, broad peaks. Additional XANES spectra of representative bacteria are shown in Fig. S3 of the Supplementary Information."

In order to identify the location of different spectra in the maps, were any PCA and cluster analysis performed, or were the spectra isolated manually only by comparison to the cryo-SXT locations?

We thank the reviewer for this suggestion. Indeed, PCA or clustering of an image can be useful to localize the regions of interest to extract spectra. In this study, Ca maps served as an equivalent to PCA to localize CDHA crystals and cells with intracellular Ca accumulation. Bacteria were then selected manually based on their position in the maps, which we considered sufficient since we did not expect additional Ca phases aside from CDHA crystals and intracellular Ca. For these reasons, PCA or clustering analysis was not applied.

The bacterial Ca is described as being in the cytosol, how is surface absorption to the outside of the bacteria ruled out?

We agree with the reviewer that 2D XANES images alone cannot rule out whether Ca is intracellular or bound at the cell surface. However, we believe that LAC quantification based on 3D tomograms acquired at the Ca L_2 edge can disclose this question. Computationally, the LAC is quantified as follows: bacteria are segmented in 3D tomographies acquired at the L2 edge. The LAC is quantified by averaging the color intensity of the voxels forming a segment, which, in the case of *B. subtilis* resulted in ≈ 550000 voxels per cell. If Ca were restricted to the bacterial envelope, the contribution from the relatively small number of voxels at the surface would be diluted by the much larger voxel population of the cytosolic volume. Reporting elevated LAC values therefore, indicates that Ca is distributed throughout the bacterial cytosol rather than confined to the cell surface.

REVIEWER COMMENTS

Reviewer #1 (Remarks to the Author):

The authors have addressed all of my comments. No further comments/questions from me. I recommend publishing the revised manuscript.

We thank the reviewer for their positive assessment of the revised manuscript and for recommending its publication. We are pleased that all comments have been satisfactorily addressed.

Reviewer #2 (Remarks to the Author):

I thank the authors for the revised version of the manuscript. The authors have carefully considered my previous comments and have repeated experiments as suggested. Overall, most of the issues I raised have been addressed. Some points have been incorporated into the manuscript, and others have been reasonably explained.
However, I would like to emphasize that explanations given only in the response-to-reviewers should also be reflected in the manuscript text itself, so that future readers will not raise the same questions again. This is particularly important for the issues discussed under Comment 3.4.
I believe that after incorporating these clarifications into the manuscript, the work will be clearer,

more informative, and at a quality suitable for Nature Communications.
I list few remaining points and clarifications below:

1. Metadata availability
As the data are not publicly available, I request that the authors provide the relevant metadata for the review process (e.g., acquisition parameters, processing steps, and any key intermediate files, as appropriate for the new data acquired on methodology).

We agree with the Reviewer on the importance of data availability. With the revised manuscript (R1), we provided an Excel file containing all raw numerical data underlying the figures presented in the manuscript, attached as Source Data file, that will be accessible to the readers. To this, we have now added another sheet with the raw data for the XANES spectra, used for running the script. The script is provided and explained in the Supplementary Information and is also available at Zenodo public repository through the following link https://doi.org/10.5281/zenodo.17297365.

Moreover, we have uploaded to the Zenodo repository the normalized tilt series, reconstructed tomographies, XANES image stacks and calcium maps related to Figures 5, 6, 7 and 8, together with a description of the file types and notes on the data processing steps. tral Beamline.

This information has been included in the Data availability section as follows:

DATA AVAILABILITY
Source Data are provided with this paper. Cryo-SXT raw data were generated at ALBA synchrotron BL09 Mistral Beamline. The normalized tilt series, reconstructed tomographies, XANES image stacks and calcium maps are deposited in the ZENODO repository at the following links: Fig. 5 (https://doi.org/10.5281/zenodo.18595868),
Fig. 6 (https://doi.org/10.5281/zenodo.18596129),
Fig. 7 & S3 (https://doi.org/10.5281/zenodo.18598093),
Fig. 8c (https://doi.org/10.5281/zenodo.18601058),
Fig. 8d (https://doi.org/10.5281/zenodo.18603081).

2. Clarification of "cell envelope damage" (line 452 and response to Comment 3.4)
In line 452, the authors refer to "cell envelope damage," and in their response to Comment 3.4 they state that the "goal was to assess the extent and nature of cellular damage". However, cell envelope damage is not clearly demonstrated in any of the provided images, or the proximity of nanopillar to a damaged wall. The method, as presented, shows differences in cytoplasm intensity, from which cell wall damage is inferred, rather than directly resolved. This is conceptually similar to the interpretation of Live/Dead staining, where membrane damage is inferred from staining colour, but the damage itself is not directly visualized. I recommend that the authors:
o Rephrase this part of the manuscript to make it explicit that cell envelope damage is inferred from intensity differences, rather than directly imaged; and/or
o Provide clearer supporting evidence, if available, that directly links the observed intensity changes to structural damage of the cell envelope.

We thank the reviewer for raising this point and for the opportunity to clarify the interpretation of the cryo-SXT images.

We would like to clarify that in some of the presented images, the cell envelope was distinguishable from the cytoplasm, and it was possible to identify regions where envelope continuity was disrupted, as indicated with white arrows in Fig. 5. That being said, we agree with the reviewer that, due to the intrinsic limitation of the technique, which does not allow simultaneous visualization of bacteria while physically attached to the nanostructured topographies, it is not possible to unambiguously attribute the observed envelope damage to direct mechanical piercing by the nanopillars.

To avoid overinterpretation, we have revised the manuscript to clarify this issue and have explicitly exposed this limitation in the Discussion section:

"Cryo-SXT revealed bacteria with a low absorption contrast suggestive of cytosolic leakage and cell wall rupture on both nanostructured substrates (Fig. 5a, white arrows). Nevertheless, due to the inability to visualize bacteria while physically attached to the nanostructured topographies, it is not possible to unambiguously attribute the observed envelope damage to direct mechanical piercing by individual nanopillars. Moreover, intracellular vesicles and multivesicular bodies, as well as endospore formation, were observed."

3. Cryo-SXT: alignment of manuscript text with the response to Comment 3.4

The response to Comment 3.4 regarding the capabilities and limitations of Cryo-SXT is helpful and improves the understanding of the approach and its goals. At present, this explanation is largely confined to the response letter. I request that these clarifications be incorporated into the main text—specifically in the Discussion and, to a more concise extent, in the Introduction—to make the goal and interpretative framework of the Cryo-SXT measurements clearer for the reader.

We thank the reviewer for this suggestion and agree that adding this information improves the understanding of the approach and the specific features of this technique. We have revised the manuscript to include this information in both the introduction and discussion, while minimizing any increase in manuscript length.

Introduction:

"This work aims to elucidate the interaction of a Gram-positive bacterium *B. subtilis,* with nanostructured calcium phosphate topographies, considering the effects of both the substrate topography and chemistry, by exploiting the versatility of synchrotron-based cryo-soft-X-rays (Cryo-SXR) transmission microscopy. Although a limitation of this technique is that it does not allow imaging cells directly attached to solid substrates and therefore the spatial relationship between bacteria and the nanopillars cannot be directly visualized, this approach enables evaluating how exposure to different nanotopographies influences bacterial morphology, subcellular organization, and calcium internalization. Cryo soft X-ray tomography (Cryo-SXT) enables three-dimensional visualization of whole hydrated, unstained cells at the nano-scale resolution[11]. Soft X-rays offer unique opportunities to study biological samples due to their specific interaction with matter in the so-called "water window" energy range (284–543 eV) between the carbon and the oxygen K absorption edges. In this range, X-rays have high penetration depth in water (up to ~10 µm)[12], while at 520 eV, the absorption by carbon-rich molecules -such as lipids and nucleic acids- is maximized."

Discussion:

"Notably, FE-SEM images evidenced the presence of severely damaged bacteria on the nanostructured surfaces, and synchrotron Cryo-SXT provided very relevant information in this respect, as it allows imaging whole bacteria in the hydrated state with minimal pre-processing beyond sonication and vitrification. Because images are acquired in transmission mode, detachment of bacteria from the substrate is required. While this represents a methodological limitation for biomaterial studies, as it does not allow explicit correlation between bacterial cell wall damage and their relative spatial position with respect to the nanopillars, the recovery of bacteria by sonication enabled us examining both intact and damaged cells that had been exposed to different conditions and compare their ultrastructure and chemical speciation. Importantly, the strong agreement between metabolic and Live/Dead assays on attached bacteria and the CFU counts of bacteria detached by sonication (Fig. 2) confirms that the sample preparation does not affect bacterial viability or mortality, ensuring the reliability of the observed effects. […]"

4. Interpretation of vesicles vs. puncture/rupture on bactericidal mechanism (Figures 7a and 5)
As presented, Figures 7a and 5 do not clearly show puncture or rupture of the cell envelope by nanopillars. Instead, the main consistent observation appears to be the presence of vesicles, which is more suggestive of a general stress response rather than direct mechanical puncturing. I suggest that the authors:
o Explicitly acknowledge in the Discussion that no unambiguous puncture/rupture events are observed in these figures; and
o Clarify that the vesicle formation is interpreted as a stress response, and discuss how this fits within the broader mechanistic picture of bacterial interaction with nanopillars.

We thank the reviewer for this comment. We agree that Figures 7a and 5 do not show unambiguous puncture or rupture of the bacterial cell envelope by individual nanopillars, due to the intrinsic limitation of the technique, which does not allow direct visualization of bacteria while in physical contact with the nanostructured topographies. We have addressed this point, which is in line with the reviewer comment n. 2, modifying the text in the Discussion section to explicitly acknowledge this limitation.

Discussion:

"Cryo-SXT revealed bacteria with a low absorption contrast suggestive of cytosolic leakage and cell wall rupture on both nanostructured substrates (Fig. 5a, white arrows). Nevertheless, due to the inability to visualize bacteria while physically attached to the nanostructured topographies, it is not possible to unambiguously attribute the observed envelope damage to direct mechanical piercing by individual nanopillars. Moreover, intracellular vesicles and multivesicular bodies, as well as endospore formation, were observed."

Regarding vesicle formation, we agree with the reviewer that this phenomenon is commonly associated with bacterial stress responses. We would like to clarify that our discussion of a potential link between cell envelope rupture and vesicle formation is based on mechanisms previously reported in the literature, and was not intended to imply that envelope damage is the cause of the vesicle formation observed in our experiments. Accordingly, we have revised the Discussion section of the manuscript to clarify that the observed vesicles are interpreted as a manifestation of cellular stress rather than as direct evidence of mechanical puncturing.

"Moreover, intracellular vesicles and multivesicular bodies, as well as endospore formation, were observed. In *B. subtilis*, the formation of vesicles and multivesicular bodies is commonly associated

with stress responses. For instance, this process has been described as a pre-lytic remodelling response that can be triggered by cell envelope damage[39] or by autolytic mechanisms aimed at destroying severely compromised cells or serving as a decoy to protect neighbouring ones.[40]"

5. Visibility of nanopillars in Cryo-SXT and identification of nanopillars (Figure 5 and 7L,F)
In the response to the question about the absence of clearly visible nanopillars in one set of Cryo-SXT images, the authors state that "identifying nanopillars in Cryo-SXT images is not straightforward due to their nanoscale dimensions and the contrast limitations of the technique." This is understandable; however, the structure indicated by the asterisk in the Figure 5 does not convincingly resemble a nanopillar. Is the dark spot next to cell in Figure5L a nanopillar? Figure 7L shows nanopillars, indeed.
I therefore ask the authors to:
o Reconsider and clarify the identification of the asterisk-marked structure in these panels;
o Provide a more cautious wording in the caption and main text, making it clear if the assignment as a nanopillar is tentative or inferred; and

We thank the reviewer for this careful observation and for the opportunity to clarify the identification of nanostructures in the Cryo-SXT images. We agree that CDHA nanopillars are more clearly visible in Figure 7 (at the Ca-edge) than in Figure 5 (at 520 eV).

Regarding Figure 5, a key criterion to distinguish calcium-deficient hydroxyapatite (CDHA) crystals from gold fiducial markers in Cryo-SXT is their relative contrast at 520 eV. At this energy, which is not optimal for calcium absorption, CDHA crystals are comparatively more transparent to soft X-rays and therefore appear in lighter grey tones, whereas gold fiducials exhibit strong absorption and appear as highly electron-dense, dark features. In addition, gold fiducials display a characteristic spherical morphology, in contrast to the elongated, needle- or rod-like morphology of CDHA crystals.

In Figure 5a, L, panel (v), the structure marked with an asterisk corresponds to CDHA crystals, identifiable by their lighter contrast and elongated morphology consistent with nano-needle or nano-rod features. By contrast, the dark spot located adjacent to the bacterium in Figures 5a (iv) and (vi) correspond to a gold fiducial, as indicated by its strong contrast and more spherical shape.

We acknowledge that identification of CDHA crystals is less straightforward in the flat CDHA samples (Figure 5a, F), as this topography does not contain nanopillars and detached crystals do not exhibit a needle-like morphology. In these cases, distinction from gold fiducials relies primarily on contrast differences and deviation from a spherical shape.

To address the reviewer's concern, we have revised the Results section to clarify the criteria used for structure identification in Figure 5, specifically explaining how CDHA crystals can be recognized in the images. With this explanation, we consider that the identification of representative features marked with asterisks in Figure 5 is clearer.

"Cryo-soft X-ray tomography sections are shown in Fig. 5. In addition to bacteria, other features can be identified: the holes of the C support film used for vitrification, some highly absorbing gold fiducials of 100 nm used for tomogram alignment and, in the samples incubated on CDHA substrates, detached CDHA crystals that were transferred during sample preparation. Notably, CDHA crystals can be distinguished from gold fiducial markers at 520 eV by their lower X-ray absorption contrast, far from the calcium absorption L-edges (~350 eV). In addition, gold fiducials display a characteristic spherical

morphology, in contrast to the elongated high-aspect ratio nanopillars observed for CDHA crystals in S and L topographies, or the clustered crystals irregular morphologies present on the flat surface (Fig. 5a). Tomographies revealed distinct features in the bacteria incubated on nanostructured topographies (Fig. 5), including damaged cell walls (white arrows), highly absorbing intracellular spots (+), and damaged bacteria showing a lighter absorbance, consistent with cytosol leakage. Bacterial ultrastructure in cells incubated on small pillars (Fig. 5 **i-iii**) revealed the formation of vesicles and multi-vesicular bodies (yellow arrows), indicative of a stress response. […]"

Subject to these relatively minor clarifications and textual adjustments—most of which aim at aligning the claims with what is directly supported by the data—I am satisfied with the scientific quality and clarity of the manuscript and consider it suitable for publication in Nature Communications.

We are grateful to the reviewer for their continued guidance in improving the clarity and precision of the manuscript, which has significantly contributed to improving its quality.

Reviewer #3 (Remarks to the Author):

The manuscript has been thoroughly revised to address the reviwer comment, adding significant new data to support their conclusion. Importantly I feel that the authors make a convincing argument for novelty regarding the use of cryoSXT and XANES in bacterial cells, and the study is indeed a good showcase for the applicability of the technique in this area.

The concerns regarding selection bias in studying only intact cells is clearly addressed
Further work has been added to address the concerns of reviewer 2, large amount of extra work to repeat SEM studies and live/dead assays with different protocols, undertake CFU and metabolic assays has been carried out and added to the manuscript. Th information added does add tot eh size of the paper, I whilst I am not convinced it was all required the improved SEM methodology and details on the sonication in particular do add weight to the studies conclusions by addressing the sample selection and preparation concerns highlighted by all reviewers. The question regarding aiming to investigate the cell wall damage relative to nanopillar location is not a claim that the authors are making in the paper so sonication does not undermine their results – the question regrading introduction of artefacts is more valid and is to me suitably addressed by the additional assays and comparison of results across methodologies.
Regarding the XANES spectroscopy and figure 6, the changes in images/projection angles used do now make the presentation clearer. The apparent difference in particle size is consistent with the explanation provided of the refocusing required for each XANES energy acquisition, and does not detract from the results or interpretation provided in the manuscript.
The clarification regarding the repeat results on a number of cells is an important point, however it is recognised as a challenge more generally with these techniques, especially when performing XANES analysis due to the time taken for the data collection – and limitations on access to SR beamtime required – this I feel should not detract from the result observed and described in this work, as long as the authors are open about the extent, and consistency of the data collected as they have been here. The explanation of the choice of the 3h time point also highlights the challenge around access to experiment time, and is fully justified.
The interpretation of the XANES spectra is consistent with the use of the technique – a background in the control no Ca case would be expected as consistent with the explanation the authors provide,

the Ca absorbance, and hence the Ca map, is obtained through the difference in the pre edge and edge images.
The addition of the Ca standard to the plots is helpful and adds clarity. The comments regarding the PCA analysis and selection of regions satisfactorily address the questions I had raised. Similarly, the question of 3D distribution using the LAC analysis is convincing
The authors have repeated the XRD analysis on different conditions and added the fit to the SI this is more convincing of the rigour of the analysis. The analysis of peak width also strengthens their comments around the crystallinity of the substrates.

We sincerely thank the reviewer for their thorough evaluation of the revised manuscript and for their positive and constructive comments. We are grateful for their recognition of the novelty of the work, the applicability of cryo-SXT and XANES to bacterial systems, and the additional experiments performed to strengthen the study. We are pleased that the revisions satisfactorily addressed the reviewer’s concerns and improved the overall clarity and robustness of the manuscript.

Synchrotron cryo-soft X-ray microscopy sheds light on the interaction of bacteria with nanostructured calcium phosphate substrates (NCOMMS-25-30132B)

Dear Editor,

We sincerely thank you and the reviewers for the continued careful evaluation of our manuscript and for the valuable comments and constructive suggestions provided during this third round of review. We have addressed all points raised, which have further improved the manuscript.

All modifications have been incorporated into the revised version and are highlighted in yellow. In this response letter, the reviewers' comments are presented in bold, our responses in blue, and the corresponding revised text excerpts are included to clearly indicate the changes made in the manuscript.

We trust that the revisions and additional clarifications fully address the remaining concerns and further strengthen the overall robustness and impact of our study. We are grateful for the opportunity to revise our work once again and appreciate your consideration of our resubmission.

REVIEWER COMMENTS

Reviewer #2 (Remarks to the Author):

I would like to thank the authors for their careful and constructive revision of the manuscript entitled "Synchrotron cryo-soft X-ray microscopy sheds light on the interaction of bacteria with nanostructured calcium phosphate substrates." The revisions demonstrate a clear effort to address the concerns raised in the previous review rounds. Well done!

Overall, the updates are satisfactory. The manuscript has been improved through the incorporation of clarifications regarding the experimental design, methodological details, limitations of the methods used and interpretation of the results. The Discussion section now appropriately reflects the points raised during review, and the scientific narrative is clearer and coherent as a result.

I also appreciate the authors' efforts toward increased data transparency, including the partial public deposition of SXT datasets. However, the live/dead confocal microscopy dataset—one of the key datasets requested in previous review—has not yet been made publicly available. Given its importance to the biological conclusions of the study, I strongly encourage the authors to deposit these data (at minimum the associated metadata and representative raw data) in an appropriate public repository to ensure reproducibility and transparency.

We thank the reviewer for this suggestion. In response, we have now deposited the live/dead confocal microscopy dataset - both Fig. 2 and Fig. S4 - in a public repository, including the confocal image files and their associated raw metadata. The repository information and accession details have been added to the Data Availability section of the manuscript. In addition, we have also deposited

the confocal microscopy dataset corresponding to the multiplexed Fluo-4 AM + CellBrite experiment (Fig. 4).

No intermediate processed files exist for these datasets, as the quantitative analysis was performed directly on the raw confocal images. The quantitative values derived from these analyses are already provided in the Source Data files associated with the relevant figures.

Now data availability has been modified as follows:

DATA AVAILABILITY

Source Data are provided with this paper. Cryo-SXT raw data were generated at ALBA synchrotron BL09 Mistral Beamline. The normalized tilt series, reconstructed tomographies, XANES image stacks and calcium maps, as well as the confocal microscopy datasets, are deposited in the ZENODO repository at the following links:

Fig. 2 (https://doi.org/10.5281/zenodo.19135539),

Fig. 4 (https://doi.org/10.5281/zenodo.19135331),

Fig. S4 (https://doi.org/10.5281/zenodo.19135718),

Fig. 5 (https://doi.org/10.5281/zenodo.18595868),

Fig. 6 (https://doi.org/10.5281/zenodo.18596129),

Fig. 7 & S3 (https://doi.org/10.5281/zenodo.18598093),

Fig. 8c (https://doi.org/10.5281/zenodo.18601058),

Fig. 8d (https://doi.org/10.5281/zenodo.18603081).

In addition, while the main text and Discussion have been appropriately revised, the Abstract appears largely unchanged from the original submission. It does not fully reflect the clarifications and refinements introduced during revision. I recommend a minor update to the Abstract to ensure consistency with the revised manuscript and to accurately represent the scope and conclusions of the study.

We thank the reviewer for this observation. We have revised the abstract to reflect the changes made throughout the manuscript which, as the reviewer correctly noted, had not been fully integrated. Specifically, we have rephrased several sentences to clarify that the bacteria were not imaged *in situ* on the substrate. The text is now more precise and emphasizes that the analysis was conducted on bacteria that had been in prior contact with the substrate.

Abstract

The rapid rise of antibiotic resistance demands for novel strategies to combat infection. This study presents synthetic bone grafts featuring nanopillared topographies designed to simultaneously promote bone regeneration while preventing bacterial colonisation. Calcium-deficient hydroxyapatite (CDHA) nanopillars, synthesized by hydrolysing α-tricalcium phosphate, showed contact-killing activity against Gram-positive B. subtilis. Unlike inert materials, CDHA bioactivity involves ionic exchange with surrounding fluids, introducing additional bacterial interaction pathways beyond

mechanical damage. Using synchrotron cryo-soft X-ray tomography and spectroscopy, we analysed bacteria that had been in contact with the nanotopographies. This enabled to simultaneously visualise mechanical damage and intracellular ionic changes under whole hydrated state at subcellular level. Following contact with the nanotopographies, some bacteria showed membrane rupture, cytosolic leakage, and multivesicular body formation, indicating stress responses. XANES cryo-spectromicroscopy and linear absorption coefficient (LAC) analysis revealed a subpopulation with elevated intracellular calcium concentrations, which is correlated with calcium release from the substrate and confirmed by confocal microscopy. Even so, it had no effect on viability, consistent with the halotolerant nature of B. subtilis. Moreover, the intracellular calcium concentration was estimated, suggesting active calcium uptake up to micromolar levels. This study opens new strategies for disentangling the role of surface chemistry and nanotopography on bacterial response.

Subject to these minor revisions, I am satisfied with the quality and presentation of the manuscript.

Reviewer #3 (Remarks to the Author):

The authors have addressed all my comment. the manuscript, SI and available data and processing routines appear to be clear and robust. I recommend publication

We thank the reviewer for their positive evaluation of our manuscript and for noting that the previous comments have been satisfactorily addressed. We are grateful for the reviewer’s recommendation for publication.
